# Body-mass index and all-cause mortality: individual-participant-data meta-analysis of 239 prospective studies in four continents

**DOI:** 10.1016/S0140-6736(16)30175-1

**Published:** 2016-08-20

**Authors:** The Global BMI Mortality Collaboration

## Abstract

**Background:**

Overweight and obesity are increasing worldwide. To help assess their relevance to mortality in different populations we conducted individual-participant data meta-analyses of prospective studies of body-mass index (BMI), limiting confounding and reverse causality by restricting analyses to never-smokers and excluding pre-existing disease and the first 5 years of follow-up.

**Methods:**

Of 10 625 411 participants in Asia, Australia and New Zealand, Europe, and North America from 239 prospective studies (median follow-up 13·7 years, IQR 11·4–14·7), 3 951 455 people in 189 studies were never-smokers without chronic diseases at recruitment who survived 5 years, of whom 385 879 died. The primary analyses are of these deaths, and study, age, and sex adjusted hazard ratios (HRs), relative to BMI 22·5–<25·0 kg/m^2^.

**Findings:**

All-cause mortality was minimal at 20·0–25·0 kg/m^2^ (HR 1·00, 95% CI 0·98–1·02 for BMI 20·0–<22·5 kg/m^2^; 1·00, 0·99–1·01 for BMI 22·5–<25·0 kg/m^2^), and increased significantly both just below this range (1·13, 1·09–1·17 for BMI 18·5–<20·0 kg/m^2^; 1·51, 1·43–1·59 for BMI 15·0–<18·5) and throughout the overweight range (1·07, 1·07–1·08 for BMI 25·0–<27·5 kg/m^2^; 1·20, 1·18–1·22 for BMI 27·5–<30·0 kg/m^2^). The HR for obesity grade 1 (BMI 30·0–<35·0 kg/m^2^) was 1·45, 95% CI 1·41–1·48; the HR for obesity grade 2 (35·0–<40·0 kg/m^2^) was 1·94, 1·87–2·01; and the HR for obesity grade 3 (40·0–<60·0 kg/m^2^) was 2·76, 2·60–2·92. For BMI over 25·0 kg/m^2^, mortality increased approximately log-linearly with BMI; the HR per 5 kg/m^2^ units higher BMI was 1·39 (1·34–1·43) in Europe, 1·29 (1·26–1·32) in North America, 1·39 (1·34–1·44) in east Asia, and 1·31 (1·27–1·35) in Australia and New Zealand. This HR per 5 kg/m^2^ units higher BMI (for BMI over 25 kg/m^2^) was greater in younger than older people (1·52, 95% CI 1·47–1·56, for BMI measured at 35–49 years *vs* 1·21, 1·17–1·25, for BMI measured at 70–89 years; p_heterogeneity_<0·0001), greater in men than women (1·51, 1·46–1·56, *vs* 1·30, 1·26–1·33; p_heterogeneity_<0·0001), but similar in studies with self-reported and measured BMI.

**Interpretation:**

The associations of both overweight and obesity with higher all-cause mortality were broadly consistent in four continents. This finding supports strategies to combat the entire spectrum of excess adiposity in many populations.

**Funding:**

UK Medical Research Council, British Heart Foundation, National Institute for Health Research, US National Institutes of Health.

## Introduction

The worldwide prevalence of overweight and obesity is high and is increasing.^1^, ^2^ WHO estimates that more than 1·3 billion adults worldwide are overweight, defined by WHO as a body-mass index (BMI) of 25–<30 kg/m^2^, and a further 600 million are obese (BMI ≥30 kg/m^2^).^3^ Appropriate analyses of large-scale prospective studies with prolonged follow-up generally indicate that both overweight and obesity are associated with increased mortality, as is underweight (defined conservatively by WHO as BMI <18·5 kg/m^2^). However, it is not known how such associations vary across major global regions, an uncertainty relevant to international strategies for overweight and obesity.^4^ Most previous analyses have focused on people living in one particular country or continent,^5^, ^6^, ^7^, ^8^, ^9^, ^10^, ^11^, ^12^ even though associations with overweight and underweight might differ from one population to another.

Estimation of the relationships between BMI and mortality in various populations can help to assess the adverse physiological effects of excessive adiposity (and the adverse physiological effects of various determinants of low BMI). However, reliable estimates of the causal relevance of BMI to mortality need to limit the effects of reverse causality, because chronic disease and smoking can themselves affect BMI. To help achieve more valid estimates, prospective studies of BMI and mortality should, when possible, exclude: smokers, participants who already have some chronic disease at recruitment that could affect BMI, and those dying within 5 years of recruitment.^13^, ^14^, ^15^, ^16^

The Global BMI Mortality Collaboration was established to provide a standardised comparison of associations of BMI with mortality across different populations. It includes individual-participant data for 10·6 million adults in 239 prospective cohort studies in 32 countries, mainly located in Asia, Australia and New Zealand, Europe, or North America, about 4 million of whom were never-smokers without reported chronic diseases (mainly cardiovascular disease, cancer, or chronic respiratory disease) at recruitment and who were still being followed up 5 years afterwards.

**Research in context**

**Evidence before this study**

A previous study has claimed that relative to normal weight (defined by WHO as a body-mass index [BMI] of 18·5–<25·0 kg/m^2^), overweight (BMI 25·0–<30·0 kg/m^2^) and grade 1 obesity (30·0–<35·0 kg/m^2^) are not associated with higher all-cause mortality. However, reliable estimates of the causal relevance of BMI to mortality should limit the effects of reverse causality, because chronic disease and smoking can themselves affect BMI. To help achieve such estimates, we established the Global BMI Mortality Collaboration, which involved analysis of individual-participant data from about 10·6 million adults in 239 prospective studies in 32 countries in Asia, Australia and New Zealand, Europe, or North America, about 4 million of whom were never-smokers without chronic disease at recruitment who were still being followed up at least 5 years afterwards.

**Added value of this study**

The Global BMI Mortality Collaboration has combined several features to help guide international public health policy. First, it involved a detailed and standardised comparison of the associations of BMI with mortality across prospective studies in four continents. Second, this analysis has been comprehensive, entailing data from 97% of eligible participants in relevant prospective cohort studies. Third, the study further subdivided the WHO's normal BMI range, which is excessively wide. Finally, the study's approach should have reduced the potentially distorting effects of smoking and ill health on BMI because the primary analyses were of never-smokers without previous disease who survived at least 5 years.

**Implications of all the available evidence**

This analysis has shown that both overweight and obesity (all grades) were associated with increased all-cause mortality. In the BMI range above 25 kg/m^2^ (the upper limit of the WHO's normal range), the relationship of BMI to mortality was strong and positive in every global region we studied (except perhaps south Asia, where numbers of deaths were small), lending support to strategies to combat the entire spectrum of excess adiposity worldwide. Our results challenge recent suggestions that overweight and moderate obesity are not associated with higher mortality, bypassing speculation about hypothetical protective metabolic effects of increased body fat in apparently healthy individuals.

## Methods

### Search strategy and selection criteria

In 2013, over 500 investigators (appendix pp 49, 50) from over 300 institutions in 32 countries agreed an analysis plan for combining individual-participant data from contributing studies. This prespecified analysis plan is provided in the appendix (pp 51–53). The goal was to produce reliable estimates of potentially causal associations of overweight and obesity with mortality using data from studies in several regions. The prespecified analysis methods were designed to maximise the internal validity by reducing the scope for bias. This Article follows PRISMA for Individual Patient Data reporting guidelines (appendix pp 54, 55).^17^

We sought data from large prospective studies (≥100 000 participants at baseline) or large multicohort consortia (total ≥100 000 participants at baseline). We identified studies published from January, 1970, to January, 2015, through systematic literature searches and discussion with investigators (appendix pp 56, 57). Electronic searches were done with MEDLINE, Embase, and Scopus, and with the terms ‘“body-mass index”, “mortality” or “death”, “cohort” or “prospective”, and combinations of the words “risk”, “relative”, “ratio”, “hazard”, or “rate”. Prospective cohort studies or consortia thereof were eligible if they: (1) had information about weight, height, age, and sex; (2) did not select participants on the basis of having any previous chronic disease; (3) recorded overall or cause-specific deaths; and (4) had accrued 5 years or more of median follow-up. We identified only two eligible studies that were unable to contribute (appendix p 37).^18^, ^19^ Details of the included studies are provided in the appendix (pp 3–14). The contributing studies classified deaths according to the primary cause (or, in its absence, the underlying cause), on the basis of coding from the International Classification of Diseases, revisions 8–10, to at least three digits (appendix p 15), or according to study-specific classification systems. Ascertainment of outcomes was generally based on death certificates, supplemented in some studies by additional data.

The appendix (p 37) describes the inclusion and exclusion criteria. We excluded participants with a BMI of less than 15 kg/m^2^ or 60 kg/m^2^ or more, or baseline age younger than 20 years or older than 90 years. To limit residual confounding by smoking and bias due to effects of pre-existing disease on baseline BMI (ie, reverse causality), the primary analysis was restricted to never-smokers without specific known chronic diseases at baseline (eg, cardiovascular disease, cancer, or respiratory diseases), and omitted the first 5 years of follow-up.

### Statistical analysis

Associations of all-cause mortality with BMI depend not only on the associations of specific causes of death with BMI in different regions (which might differ quantitatively), but also on how relatively common each specific cause of death is in the particular region (which can differ substantially by region and over time). Hence, the association of all-cause mortality with BMI might differ in regions with different underlying mortality patterns. Therefore, the prespecified primary analysis was stratified by five major geographical regions, three with extensive data (east Asia, Europe, and North America) and two with more limited data (Australia and New Zealand, and south Asia). Data from some or all regions are shown separately, in the main text or in the appendix.

Each study (or consortium of studies) analysed individual-participant data according to a common analytical plan with SAS version 9.3 (SAS Institute, Cary, NC, USA) or Stata version 12 (StataCorp, College Station, TX, USA) provided by the coordinating centres. These separate results were then meta-analysed at Cambridge University, UK. To facilitate standardised comparisons with other meta-analyses, we calculated hazard ratios (HRs) for mortality in the six WHO-defined baseline BMI categories: underweight (15·0–<18·5 kg/m^2^), normal (18·5–<25·0 kg/m^2^; the reference category for analyses of these six BMI groups), overweight (25–<30·0 kg/m^2^), and obesity grade 1 (30·0–<35·0 kg/m^2^), grade 2 (35·0–<40·0 kg/m^2^), and grade 3 (40·0–<60·0 kg/m^2^).^20^ Because, however, most people are of normal weight or overweight, these two categories were subdivided, yielding nine groups (15·0–<18·5 kg/m^2^; 18·5–<20·0 kg/m^2^; 20·0–<22·5 kg/m^2^; 22·5–<25·0 kg/m^2^, the reference category for analyses of nine BMI groups; 25·0–<27·5 kg/m^2^; 27·5–<30·0 kg/m^2^; 30·0–<35·0 kg/m^2^; 35·0–<40·0 kg/m^2^; and 40·0–<60·0 kg/m^2^). The BMI group with the largest number of participants was chosen as the reference group.

Study-specific log HRs in specific BMI categories were pooled by inverse-variance-weighted random-effects meta-analyses (an extension of the DerSimonian and Laird procedure) and plotted against the mean BMI value within each category. Sensitivity analyses used other statistical methods (eg, fixed-effect models). To enable comparisons across BMI groups irrespective of the choice of a reference group, a floating variance estimate (reflecting independent variability within each group, including the reference group) was attributed to each category using Plummer's method and used to calculate group-specific confidence intervals.^21^

To estimate the BMI levels at which mortality risk was lowest (ie, the nadir), weighted linear regression yielded the best-fitting second-degree fractional polynomial model relating pooled log HRs to pooled mean BMI levels (weighted by the inverse of the floating variance of the log HR), and the minimum of this polynomial was the nadir. We assessed all-cause mortality and its main components, coronary heart disease, stroke, other cardiovascular disease, cancer, and respiratory disease (appendix p 15). HRs were calculated separately within each study with Cox regression models stratified for baseline age and sex (appendix pp 51–53), with participants contributing from the baseline survey in crude analyses or from year 5 in the primary analyses. HRs in sex-specific and baseline-age-specific groups (and, when appropriate, by trial groups) were combined across studies.^22^ To avoid over-fitting of statistical models, studies with ten or fewer deaths from a particular cause were excluded from meta-analyses of that cause.^23^, ^24^

Because the associations of BMI with mortality were approximately log-linear above a BMI of 25 kg/m^2^, we calculated HRs per 5 kg/m^2^ higher BMI increase by inverse-variance-weighted regression of the pooled log HRs on mean BMI values in each category.^17^ For all-cause mortality, we estimated population-attributable fractions for underweight, overweight, and obesity by combining the proportional excess mortality (X_0_, X_1_, and X_2_, where X=HR-1) in these BMI categories with the corresponding prevalences (P_0_, P_1_, and P_2_, taken from Global Burden of Disease^25^ region-specific prevalences). The population-attributable fractions for overweight and obesity are then P_1_X_1_/k and P_2_X_2_/k, where k=1 + P_0_X_0 _+ P_1_X_1 _+ P_2_X_2_. Between-study heterogeneity was quantified by the *I*^2^ statistic.^26^ We used two-sided p values and 95% CIs.

### Role of the funding source

The funders of the study had no role in study design, data collection, data analysis, data interpretation, or writing of the report. SK, PG, EDA, and JD had access to all the data, and, together with SNB and FBH, were responsible for the decision to submit for publication.

## Results

Of 10 625 411 participants from 239 studies (median follow-up 13·7 years, IQR 11·4–14·7), 3 951 455 people in 189 studies were never-smokers without specific chronic diseases at recruitment who survived 5 years, of whom 385 879 died. To limit bias, the prespecified primary analyses involved this restricted population. To avoid merging importantly different risks, many of these primary analyses further subdivided the WHO-defined normal and overweight BMI categories, yielding nine BMI groups rather than six.

Table 1 shows the substantial relevance of successively stricter exclusions, going from crude analyses of about 10·6 million to prespecified analyses of about 4 million adults. With BMI in only six groups, the whole range from 18·5 kg/m^2^ to less than 25 kg/m^2^ is the reference group, and HRs were: underweight 1·47 (95% CI 1·39–1·55), overweight 1·11 (1·10–1·11), grade 1 obesity 1·44 (1·41–1·47), grade 2 obesity 1·92 (1·86–1·98), grade 3 obesity 2·71 (2·55–2·86), and any obesity 1·64 (1·61–1·67; appendix pp 16, 17, 25). With normal and overweight groups more finely subdivided, however, BMI 22·5 kg/m^2^ to less than 25·0 kg/m^2^ becomes the reference group, and with this more precise reference group, the HRs for grade 1, 2, and 3 obesity increased slightly (table 1, 2). Mortality was lowest in the BMI range from 20·0 kg/m^2^ to less than 25·0 kg/m^2^, and was significantly increased just below this BMI range and in the overweight range just above it (table 2).

**Figure 1:**
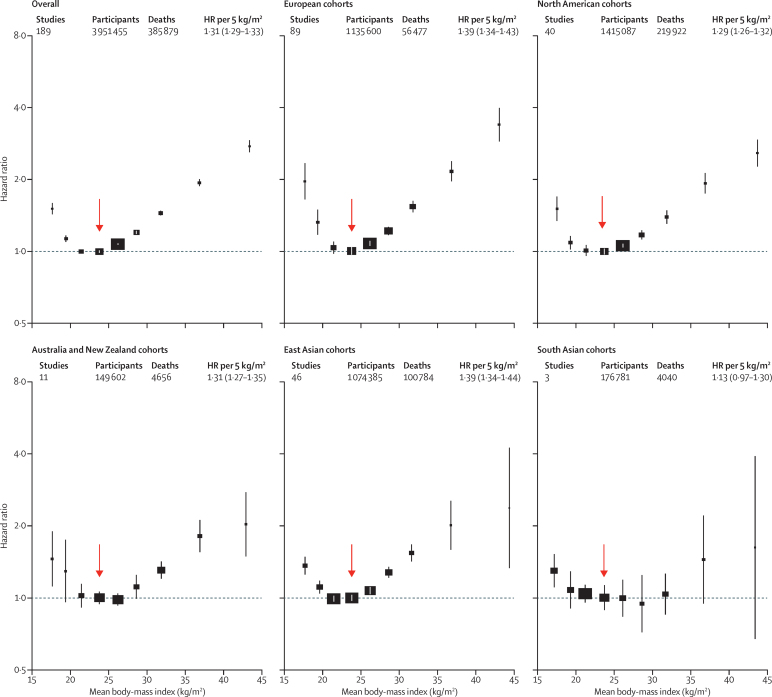
Association of body-mass index with all-cause mortality, by geographical region Boxes are plotted against the mean BMI in each group. The HR per 5 kg/m^2^ higher body-mass index (BMI) and its 95% CI are calculated only for BMI more than 25·0 kg/m^2^. Analyses restricted to never-smokers without pre-existing chronic disease, excluding the first 5 years of follow-up. The reference category is shown with the arrow and is 22·5–<25·0 kg/m^2^. CIs are from floating variance estimates (reflecting independent variability within each category, including reference). Areas of squares are proportional to the information content (ie, inverse of the floating variance). HR=hazard ratio.

In these prespecified analyses of almost 4 million adults, the HRs for overweight and for obesity grade 1 were broadly similar across different geographical regions (Europe, North America, east Asia, and Australia and New Zealand; numbers of deaths in south Asia were too small to be reliable), but the HRs for underweight and grade 3 obesity appeared somewhat higher in Europe than in east Asia (figure 1, table 3, appendix pp 16–21).

**Figure 2:**
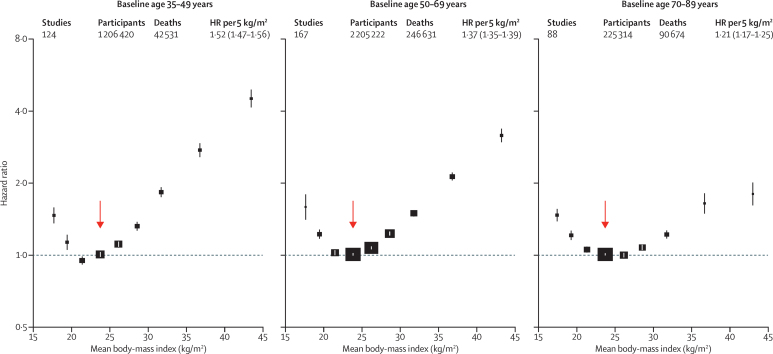
Association of body-mass index with all-cause mortality, by baseline age group The HR per 5 kg/m^2^ higher body-mass index (BMI) and its 95% CI are calculated only for BMI more than 25·0 kg/m^2^. Analyses restricted to never-smokers without pre-existing chronic disease, and excluding the first 5 years of follow-up, and include data from all geographical regions. The reference category is shown with the arrow and is 22·5–<25·0 kg/m^2^. CIs are from floating variance estimates (reflecting independent variability within each category, including the reference category). Areas of squares are proportional to the information content. Analyses by baseline age and the three main geographical regions are in the appendix (p 38). HR=hazard ratio.

**Figure 3:**
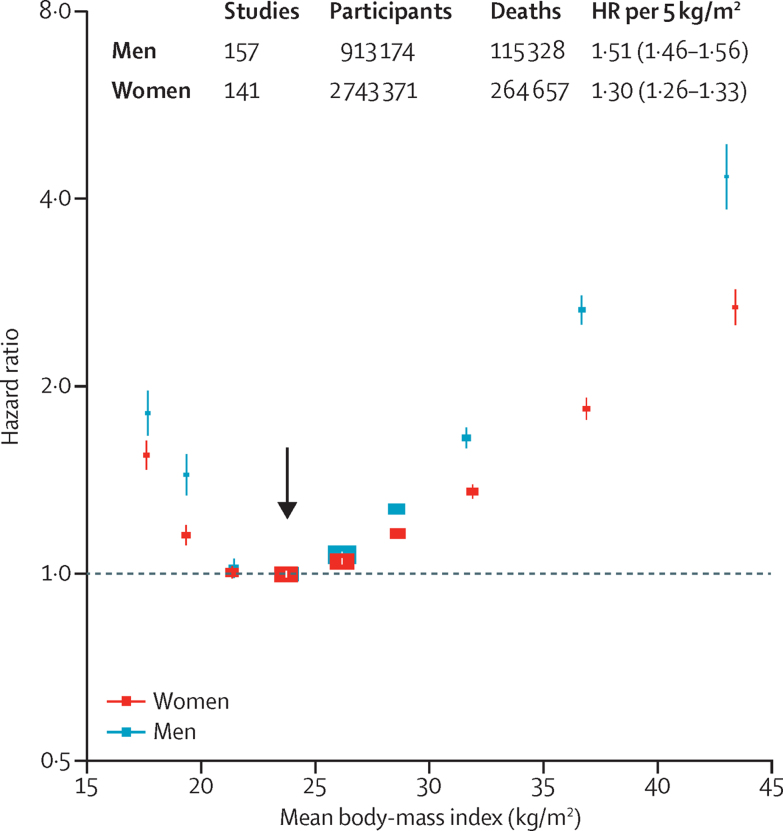
Association of body-mass index with all-cause mortality, by sex The HR per 5 kg/m^2^ higher body-mass index (BMI) and its 95% CI are calculated only for BMI more than 25·0 kg/m^2^. Analyses restricted to never-smokers without pre-existing chronic disease, excluding the first 5 years of follow-up, and include data from all geographical regions. The reference category is shown with the arrow and is 22·5–<25·0 kg/m^2^. CIs are from floating variance estimates (reflecting independent variability within each category, including reference). Areas of squares are proportional to the information content. Analyses by sex and the three main geographical regions (east Asia, Europe, and North America) are in the appendix (p 39). HR=hazard ratio.

Combining all regions, the HRs for overweight and obesity were higher at younger ages than older ages, and in men than women (Figure 2, Figure 3); this finding held in each major geographical region (appendix pp 22–24 38–40). In each region, BMI was non-linearly associated with all-cause mortality, with nadir at BMI 20·0 kg/m^2^ to less than 25·0 kg/m^2^ and excess mortality in underweight, overweight, and at BMI 18·5 kg/m^2^ to less than 20·0 kg/m^2^, at the lower end of the WHO-defined normal range. The nadir depended on age, and was BMI=22 kg/m^2^ for baseline age 35–49 years, BMI=23 kg/m^2^ for baseline age 50–69 years, and BMI=24 kg/m^2^ for baseline age 70–89 years.

Population-attributable fractions for all-cause mortality due to overweight or obesity were 19% in North America, 16% in Australia and New Zealand, and 14% in Europe, but only 5% in east Asia (appendix p 25). For BMI 25 kg/m^2^ or more, the association of BMI with all-cause mortality was approximately log-linear, and of similar strength in each region (except perhaps south Asia, where numbers of deaths were small), with HR per 5 kg/m^2^ units higher BMI 1·31 (95% CI 1·29–1·33) overall, 1·39 (1·34–1·44) in east Asia, 1·39 (1·34–1·43) in Europe, 1·29 (1·26–1·32) in North America, and 1·31 (1·27–1·35) in Australia and New Zealand. The HR decreased with age from 1·52 (1·47–1·56) for ages 35–49 years at baseline to 1·21 (1·17–1·25) for ages 70–89 years at baseline (trend p<0·0001; appendix p 26). The HR was 1·51 (1·46–1·56) for men versus 1·30 (1·26–1·33) for women (heterogeneity p<0·0001; figure 3). Hence, a given increase in BMI is associated with a far greater absolute mortality increase in men than in women (appendix p 45). As there were far more women than men, particularly among obese people, the HR among all participants was similar to the HR just among women.

**Figure 4:**
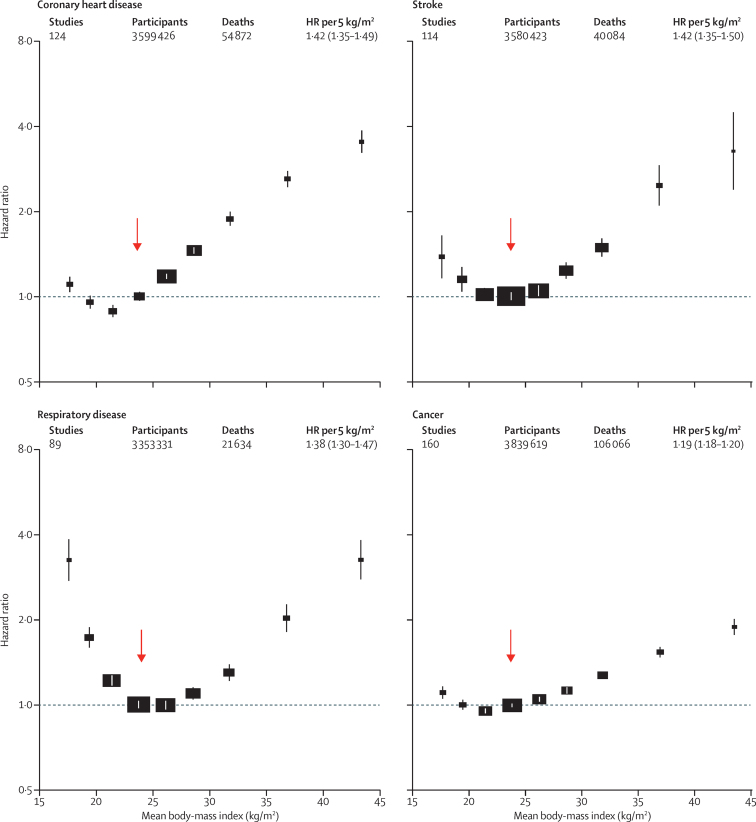
Association of body-mass index with mortality, by major underlying cause The HR per 5 kg/m^2^ higher body-mass index (BMI) and its 95% CI are calculated only for BMI more than 25·0 kg/m^2^. Analyses restricted to never-smokers without pre-existing chronic disease, excluding the first 5 years of follow-up, and include data from all geographical regions. The reference category is shown with the arrow and is 22·5–<25·0 kg/m^2^. CIs are from floating variance estimates (reflecting independent variability within each category, including reference). Areas of squares are proportional to the information content. Analyses of cause-specific mortality by three geographical regions (east Asia, Europe, and North America) are in the appendix (pp 41, 42).

For each major cause of death, BMI was non-linearly associated with mortality in each major region we studied (appendix pp 27–29, 41, 42). Above 25 kg/m^2^, BMI was strongly positively related to coronary heart disease, stroke, and respiratory disease mortality, and moderately positively related to cancer mortality (figure 4); these findings were broadly similar in Europe, North America, and east Asia (appendix pp 28, 29). Within WHO's wide normal BMI range (18·5–<25·0 kg/m^2^) the main geographical difference was that, in east Asia, mortality from coronary heart disease had its nadir at 18·5–<20·0 kg/m^2^, lower than in other regions (appendix p 28). In all regions, underweight was associated with substantially higher respiratory disease mortality and somewhat higher mortality from coronary heart disease, stroke, and cancer (figure 4). HRs comparing underweight versus normal-weight cardiovascular disease mortality were more extreme in Europe than elsewhere (appendix pp 28, 29).

Compared with the strict primary analyses described above, crude analyses that ignored smoking and any effects of prior disease at baseline, and did not exclude the first 5 years of follow-up, yielded different (presumably substantially biased) results, with exaggerated HRs for underweight, inverted HRs for overweight, and less than half of the excess risk for grade 1 obesity suggested by the strict primary analyses (Table 1, Table 2, appendix p 43).

In sensitivity analyses (appendix pp 30–36, 46–48), HRs were little changed in analyses that used fixed effect models or restricted follow-up to years 5–15; considered age at risk rather than age at baseline; adjusted additionally for race or excluded participants with diabetes at baseline; used only studies that included both sexes; used only studies with baseline data for heart disease, stroke, and cancer; or subdivided studies by mean baseline BMI or median recruitment year (HRs were somewhat higher in studies starting before 1990 than those after 1990, but meta-regression of HRs on year of recruitment was not significant). HRs did not vary substantially between larger and smaller studies, between studies with measured and self-reported BMI, or between occupational and other studies.

## Discussion

Associations between BMI and mortality can help to estimate the public health impact of excess adiposity only if the estimated relationships are not substantially distorted by the effects of smoking or ill health on BMI. Hence, our primary analyses were of never-smokers without previous disease who survived at least 5 years. Both overweight and obesity were associated with increased all-cause mortality. In the BMI range above 25 kg/m^2^ (ie, above the upper limit of the WHO's normal range) the relationship to mortality was steep in every global region we studied, except perhaps south Asia where numbers of deaths were small.^27^

Our primary analyses challenge previous suggestions that overweight (25–<30 kg/m^2^) and grade 1 obesity (30–<35 kg/m^2^) are not associated with higher mortality,^28^ bypassing speculation about hypothetical protective metabolic effects of increased body fat in apparently healthy individuals.^29^ In particular, the findings here contrast with those of a 2013 review that claimed that, relative to normal weight, grade 1 obesity was not associated with excess all-cause mortality and that overweight was associated with lower all-cause mortality.^28^ That review could not, however, control for the biases controlled for in our analysis. Indeed, the results of the current analysis (eg, table 1, table 2, and appendix pp 16, 17) show how the limited ability of that literature-based review to control for bias could have accounted for its misleading findings. Our study was able to reproduce such findings when conducting crude analyses with inadequate control of reverse causality, but not when we conducted appropriately strict analyses.

Despite broadly similar overall findings across different continents, we found some differences. HRs per 5 kg/m^2^ higher BMI above 25 kg/m^2^ appeared to be somewhat greater in Europe than in North America. In each major region we studied, HRs were substantially higher at younger than at older ages, although the absolute excess mortality was higher in older people. HRs were substantially higher in men than in women, consistent with previous observations that, at equivalent BMI levels, men have greater insulin resistance, ectopic (eg, liver) fat levels, and type 2 diabetes prevalence.^30^ Our primary analyses of never-smokers included, however, far more women than men, particularly at higher BMI levels. Hence, our HRs for obesity (and, above 25·0 kg/m^2^, the excess HR per 5 kg/m^2^ increase in BMI) mainly describe effects in women, despite the substantially larger HRs in men. Our HRs for grade 1 obesity (male 1·70, female 1·37; appendix p 22) suggest that men have almost double the proportional excess mortality of women— but, as age-specific death rates are typically more than 50% higher in men, the absolute excess death rate associated with grade 1 obesity is about three times as great in men (appendix p 45).

Because the prevalence of obesity differs by region, for all-cause mortality there was wide variation across regions in the approximate population-attributable fraction due to overweight and obesity. These findings suggest that if the overweight and obese population had WHO-defined normal levels of BMI, the proportion of premature deaths that could be avoided would be about one in five in North America, one in six in Australia and New Zealand, one in seven in Europe, and one in 20 in east Asia, assuming that the associations of overweight and obesity with mortality in our primary analyses largely reflect causal effects. Moreover, BMI is increasing in many populations, so the pattern of high mortality from adiposity in North America might become typical elsewhere.^31^ At the opposite extreme, there was a substantially higher mortality not only among those in WHO's underweight category, but also in those with BMI 18·5 kg/m^2^ to <20 kg/m^2^, suggesting that in excessively lean adult populations underweight remains a cause for concern. We have no information about whether the BMI in underweight individuals was always low.

Our primary analyses used three main approaches to help avoid bias. First, we restricted analysis to never-smokers to avoid residual confounding by smoking as far as possible because merely adjusting for smoking habits would be unlikely to eliminate important residual biases due to the effect on BMI of different intensities of smoking.^13^ Second, we sought to exclude people known to have specific pre-existing chronic diseases (although full information about this variable was often unavailable). Finally, we omitted the initial 5 years of follow-up from the analysis because diseases at baseline that might cause death over the next 5 years could result in reverse causation (where lower BMI at recruitment is the result, rather than the cause, of the underlying pathology).^14^, ^15^, ^16^

Our findings are consistent with other (albeit less precise) studies that have used effective methods to reduce potential bias in evaluations of a causal relationship between excess BMI and mortality, such as Mendelian randomisation analyses,^32^, ^33^ other instrumental variable analyses,^34^ and a meta-analysis of randomised trials.^35^ Our findings are also broadly consistent with the stricter analyses done in a 2015 study^36^ of 12 million Korean adults and with a 2016 review that attempted to limit the effects of reverse causality.^37^

The most important limitation is that our only measure of adiposity was BMI, so we could not directly address aspects of body composition such as visceral fat or fat distribution,^38^, ^39^ nor could we consider modification of HRs by metabolic factors.^40^ Such factors might have different effects in different populations because, at the same BMI, people of Asian ancestry might have higher amounts of body fat and greater risk of developing metabolic diseases than people of European ancestry.^41^ Moreover, south Asia, Africa, and Latin America were either unrepresented or poorly represented, and large studies in those areas might yield different findings. The study-specific results were in general not adjusted for ethnicity or for socioeconomic status. We did not adjust for regression dilution because previous surveys have reported high levels of concordance in replicate BMI measures taken from the same adults some years apart.^42^

There are, however, particular strengths. Compared with single-country studies, we enhanced generalisability by combining findings from 239 studies across four continents. We had access to data for about 97% of the participants in the studies eligible for this analysis (giving large numbers and negligible bias from unavailability of particular studies), we used a prespecified analysis plan, we analysed individual-participant data to avoid the potentially important limitations of literature-based reviews,^43^ and we analysed clinically relevant subpopulations reliably, exploiting the considerable statistical power of the study. We avoided potential over-adjustment by not adjusting for variables (eg, diabetes status and physical activity) that could mediate associations between BMI and mortality.^44^ Finally, our results were robust to a variety of sensitivity analyses.

We conclude that wherever overweight and obesity are common their associations with higher all-cause mortality are broadly similar in different populations, supporting strategies to combat the entire spectrum of excessive adiposity worldwide.

Correspondence to: Prof John Danesh, Department of Public Health and Primary Care, University of Cambridge, Cambridge CB1 8RN, England, UK **gbmc@phpc.cam.ac.uk**

## Figures and Tables

**Table 1:** Effects of successively stricter precautions against bias on analyses of six WHO defined groups of BMI versus all-cause mortality

	**Underweight (15·0– <18·5 kg/m^2^)**	**Normal weight (18·5– <25·0 kg/m^2^)**	**Overweight (25·0– <30·0 kg/m^2^)**	**Obesity grade 1 (30·0– <35·0 kg/m^2^)**	**Obesity grade 2 (35·0– <40·0 kg/m^2^)**	**Obesity grade 3 (40·0– <60·0 kg/m^2^)**
Crude analysis with no exclusions*						
Participants/deaths	292 003/68 455	5 586 892/810 838	3 467 617/526 098	946 257/144 871	237 223/36 113	92 458/15 399
HR (95% CI)	1·82 (1·74–1·91)	1·00 (0·98–1·02)	0·95 (0·94–0·97)	1·17 (1·16–1·18)	1·49 (1·47–1·51)	1·95 (1·90–2·01)
**Participants without known disease at baseline**†						
Participants/deaths	255 000/52 789	4 922 817/631 488	2 916 978/388 781	756 075/102 315	183 689/24 556	696 88/10 321
HR (95% CI)	1·81 (1·72–1·91)	1·00 (0·98–1·02)	0·95 (0·95–0·96)	1·18 (1·16–1·20)	1·52 (1·48–1·55)	2·05 (1·98–2·13)
**Participants without known chronic disease at baseline, adjusted for smoking status**‡						
Participants/deaths	245 080/51 170	4 751 019/618 881	2 826 687/381 617	733 108/100 113	178 130/23 945	67 593/10 002
HR (95% CI)	1·70 (1·61–1·80)	1·00 (0·98–1·02)	0·99 (0·98–1·00)	1·25 (1·23–1·27)	1·63 (1·59–1·66)	2·24 (2·15–2·33)
**Participants without known chronic disease at baseline, adjusted for smoking status, and excluding the first 5 years of follow-up**§						
Participants/deaths	208 044/33 817	4 234 052/496 310	2 513 128/312 450	641 237/80 037	152 741/18 737	56 232/7 659
HR (95% CI)	1·60 (1·51–1·70)	1·00 (0·98–1·02)	1·03 (1·01–1·04)	1·31 (1·29–1·33)	1·70 (1·67–1·74)	2·36 (2·27–2·45)
**The primary prespecified analysis: never-smokers without known chronic disease at baseline—excluding the first 5 years of follow-up**¶						
Participants/deaths	114 091/12 726	2 145 550/192 523;	1 250 103/130 293;	330 840/37 318	80 827/9 179	30 044/3 840
HR (95% CI)	1·47 (1·39–1·55)	1·00 (0·98–1·02)	1·11 (1·10, 1·11)	1·44 (1·41–1·47)	1·92 (1·86–1·98)	2·71 (2·55–2·86)

CIs were calculated with floating variance estimates (reflecting independent variability within each group, including the reference group). Reference group is normal weight (18·5–<25·0 kg/m^2^). All analyses are adjusted for age and sex. Baseline BMI categories were defined by WHO. BMI=body-mass index. HR=hazard ratio.

* 237 studies; 10 622 450 participants; 1 601 774 deaths.

† 236 studies; 9 104 247 participants; 1 210 250 deaths.

‡ 234 studies; 8 801 617 participants; 1 185 728 deaths.

§ 213 studies; 7 805 434 participants; 949 010 deaths.

¶ 189 studies; 3 951 455 participants; 385 879 deaths.

**Table 2:** Nine groups of BMI versus all-cause mortality, with use of the primary prespecified analysis

	**15·0–<18·5 kg/m^2^**	**18·5–<20·0 kg/m^2^**	**20·0– <22·5 kg/m^2^**	**22·5–<25·0 kg/m^2^**	**25·0–<27·5 kg/m^2^**	**27·5–<30·0 kg/m^2^**	**30·0–<35·0 kg/m^2^**	**35·0–<40·0 kg/m^2^**	**40·0–<60·0 kg/m^2^**
Participants/deaths	114 091/12 726	230 749/20 989	838 907/72 701	1075 894/98 833	821 303/84 952	428 800/45 341	330 840/37 318	80 827/9 179	30 044/3 840
HR (95% CI)	1·51 (1·43–1·59)	1·13 (1·09–1·17)	1·00 (0.98–1.02)	1·00 (0·99–1·01)	1·07 (1·07–1·08)	1·20 (1·18–1·22)	1·45 (1·41–1·48)	1·94 (1·87–2·01)	2·76 (2·60–2·92)

189 studies; 3 951 455 participants; 385 879 deaths. The primary prespecified analysis in never-smokers without known chronic disease at baseline, excluding the first 5 years of follow-up (with normal weight and overweight categories further subdivided into: 18·5–<20·0 kg/m^2^, 20·0–<22·5 kg/m^2^, 22·5–<25·0 kg/m^2^, 25·0–<27·5 kg/m^2^, and 27·5–<30·0 kg/m^2^). CIs were calculated using floating variance estimates (reflecting independent variability within each group, including the reference group). Reference group is 22·5–<25·0 kg/m^2^. All analyses are adjusted for age and sex. Baseline BMI categories were defined by WHO. BMI=body-mass index. HR=hazard ratio.

**Table 3:** Nine BMI groups versus all-cause mortality in never-smokers, excluding chronic disease at baseline and 5 years of follow-up in geographical regions with more than 1 million participants

		**15·0–<18·5 kg/m^2^**	**18·5–<20·0 kg/m^2^**	**20·0–<22·5 kg/m^2^**	**22·5–<25·0 kg/m^2^**	**25·0–<27·5 kg/m^2^**	**27·5–<30·0 kg/m^2^**	**30·0–<35·0 kg/m^2^**	**35·0–<40·0 kg/m^2^**	**40·0–<60·0 kg/m^2^**
Europe*										
	Participants/deaths	13 398/675	42 584/1508	199 369/7449	306 566/13278	249 929/12 850	153 147/8935	127 536/8386	32 749/2424	10 322/972
	HR (95% CI)	1·79 (1·63–1·97)	1·25 (1·14–1·38)	1·02 (0·97–1·07)	1·00 (0·97–1·03)	1·07 (1·06–1·09)	1·21 (1·18–1·25)	1·52 (1·45–1·58)	1·99 (1·87–2·12)	3·04 (2·84–3·27)
North America†										
	Participants/deaths	22 028/3846	67 114/8597	274 883/36 200	359 022/54 995	317 721/53 464	168 183/28 471	149 807/25 348	39 379/6299	16 950/2702
	HR (95% CI)	1·51 (1·34–1·70)	1·09 (1·02–1·16)	1·01 (0·96–1·06)	1·00 (0·97–1·03)	1·06 (1·04–1·07)	1·17 (1·12–1·22)	1·39 (1·30–1·49)	1·93 (1·74–2·13)	2·58 (2·26–2·93)
East Asia‡										
	Participants/deaths	46 979/7178	94 409/10 206	301 242/27 537	336 758/28 755	194 857/17 070	72 133/6950	25 658/2753	1941/231	408/104
	HR (95% CI)	1·36 (1·25–1·49)	1·11 (1·04–1·18)	0·99 (0·97–1·02)	1·00 (0·97–1·03)	1·07 (1·04–1·11)	1·28 (1·21–1·35)	1·54 (1·42–1·67)	2·01 (1·59–2·54)	2·38 (1·33–4·24)
p value for heterogeneityl¶		0·0045	0·28	0·42	..	0·89	0·46	0·20	0·48	<0·0001

Normal weight and overweight are subdivided, and the reference category is BMI 22·5 kg/m^2^ to less than 25·0 kg/m^2^. Numbers of studies, participants, and deaths are shown after exclusions from these prespecified principal analyses. CIs were calculated using floating variance estimates (reflecting independent variability within each group, including the reference group). Results from studies in south Asia and Australia and New Zealand are in figure 1, with details in the appendix (p 20).

* 89 studies; 1 135 600 participants; 56 477 deaths.

† 40 studies; 1 415 087 participants; 219 922 deaths.

‡ 46 studies; 1 074 385 participants; 100 784 deaths.

¶ p value for heterogenity is for all three regions.
