# Supplementary material for: Body-mass index and all-cause mortality: individual-participant-data meta-analysis of 239 prospective studies in four continents
Source: Lancet. 2016 Aug 20;388(10046):776–86. doi: 10.1016/S0140-6736(16)30175-1 (PMC4995441; doi:10.1016/S0140-6736(16)30175-1)
Supplement: Supplementary appendix [file mmc1.pdf]

THE LANCET

## Supplementary appendix

This appendix formed part of the original submission and has been peer reviewed. We post it as supplied by the authors.

Supplement to:The Global BMI Mortality Collaboration. Body-mass index and all-cause mortality: individual-participant-data meta-analysis of 239 prospective studies in four continents. *Lancet* 2016; published online July 13. http://dx.doi.org/10.1016/S0140-6736(16)30175-1

**The Global BMI Mortality Collaboration: Body-mass index and all-cause mortality Individual-participant-data meta-analysis of 239 cohorts across four continents**

## Contents list of supplementary material: CLICK on any item to jump to it

Note: WHO defines **6** BMI groups (underweight, normal, overweight and grades I, II and III obesity).
But, finer subdivision of the WHO normal and overweight groups yields the **9** finer groups used here.

### Supplementary Tables


| Pages | eTable no. | |
|---|---|---|
| 3-10 | 1 | - Baseline characteristics of the 239 prospective studies |
| 11-14 | 2 | - Chronic diseases recorded at baseline in each study |
| 15 | 3 | - Mortality outcomes and their ICD codes |
| 16 | 4 | **6** BMI groups vs all mortality, by successively stricter inclusion criteria, in all 237 analysed studies |
| 17 | 5 | **6** BMI groups vs all mortality, by successively stricter inclusion criteria, in 189 fully included studies |
| 18 | 6 | **6** BMI groups vs all mortality, by smoking status |
| 19 | 7 | **6** BMI groups vs all mortality, using standard CIs (rather than floating absolute risks) |
| 20 | 8 | **9** BMI groups vs all mortality, by geographic region |
| 21 | 9 | **6** BMI groups vs all mortality, by geographic region |
| 22 | 10 | **6** BMI groups vs all mortality, by geographic region and sex |
| 23-24 | 11 | **6** BMI groups vs all mortality, by geographic region and baseline age |
| 25 | 12 | - Population-attributable fractions for overweight and obesity |
| 26 | 13 | - Steepness of BMI vs all mortality for BMI>25 kg/m$^2$, by age and sex |
| 27 | 14 | - Steepness of BMI vs CAUSE-SPECIFIC mortality for BMI>25 kg/m$^2$, by geographic region |
| 28 | 15 | **9** BMI groups vs CAUSE-SPECIFIC mortality, by geographic region |
| 29 | 16 | **6** BMI groups vs CAUSE-SPECIFIC mortality, by geographic region |
| 30 | 17 | **9** BMI groups vs all mortality, (a) by fixed-effect methods and (b) in years 5-15 only |
| 31 | 18 | **6** BMI groups vs all mortality, (a) by fixed-effect methods and (b) in years 5-15 only |
| 32 | 19 | **6** BMI groups vs all mortality, effects of adjustment for race, or exclusion of baseline diabetes |
| 33 | 20 | **6** BMI groups vs all mortality, by completeness of data on chronic diseases at baseline |
| 34 | 21 | **6** BMI groups vs all mortality, by year of baseline survey |
| 35 | 22 | **6** BMI groups vs all mortality, by method of assessing BMI |
| 36 | 23 | **6** BMI groups vs all mortality, by occupational/other population source |


### Supplementary Figures


| Pages | eFigure no. | |
|---|---|---|
| 37 | 1 | - Flow diagram of literature search |
| 38 | 2 | **9** BMI groups vs all mortality, by baseline age and geographic region |
| 39 | 3 | **9** BMI groups vs all mortality, by sex and geographic region |
| 40 | 4 | **9** BMI groups vs all mortality, by sex and geographic region in studies with both men and women |
| 41-42 | 5 | **9** BMI groups vs CAUSE-SPECIFIC mortality, by geographic region |
| 43 | 6 | **9** BMI groups vs all mortality, by successively stricter inclusion criteria |
| 44 | 7 | **9** BMI groups vs all mortality, by age at risk |
| 45 | 8 | **9** BMI groups vs all mortality, absolute risks corresponding to the overall relative risks |
| 46 | 9 | - Steepness of BMI vs all mortality for BMI>25 kg/m$^2$ in each study, plotted vs mean BMI in that study |
| 47 | 10 | - Steepness of BMI vs all mortality for BMI>25 kg/m$^2$ in each study, plotted vs year of baseline survey |
| 48 | 11 | - Steepness of BMI vs all mortality for BMI>25 kg/m$^2$ in each study, plotted vs size of that study |


### Supplementary Appendices


| Pages | eAppendix no. | |
|---|---|---|
| 49-50 | 1 | - Investigators in each study |
| 51-53 | 2 | - Pre-specified analysis plan |
| 54-55 | 3 | - PRISMA-IPD checklist |
| 56-57 | 4 | - Systematic literature search procedures |
| 58-63 | 5 | - References |
| 64-67 | 6 | - Text-figures 1-4 |

# eTable 1: Baseline characteristics of participating studies

| Study | Ref | Country | Population source | Weight & height assessment | Total participants | Mean (SD) BMI | Mean (SD) age at survey | Mean (SD) age at death | No (%) males | No (%) current smokers | Median baseline survey year | Median (IQR) follow-up time | Death ascertainment | Number of deaths | | | | | |
|---|---|---|---|---|---|---|---|---|---|---|---|---|---|---|---|---|---|---|---|
| | | | | | | | | | | | | | | All-cause mortality | CVD | CHD | Stroke | Cancer | Respiratory disease |
| **Europe** | | | | | | | | | | | | | | | | | | | |
| Vorarlberg Health Monitoring and Promotion Programme | 1 | Austria | Health check-up | measured | 120981 | 25.4 (4.2) | 47.9 (13.6) | 70.0 (8.0) | 55291 (46) | 22621 (19) | 1988 | 13.2 (8.9, 15.6) | + | 7126 | 3338 | 1706 | 796 | 2387 | 297 |
| Belgian Inter-university Research on Nutrition and Health | 2 | Belgium | Electoral roll | measured | 11053 | 26.0 (4.1) | 48.8 (13.3) | 68.0 (10.0) | 5820 (53) | 3672 (33) | 1982 | 10.5 (10.5, 10.5) | ++ | 1051 | 422 | 164 | 82 | 326 | 54 |
| Flemish Study on Environment, Genes and Health (FLEMENGHO) | 3 | Belgium | General popln. | measured | 1342 | 25.7 (4.3) | 47.9 (15.9) | 73.0 (12.0) | 656 (49) | 490 (37) | 1987 | 7.5 (6.5, 9.5) | + | 112 | 50 | 14 | 14 | 38 | 8 |
| Seven Countries Croatia | 4 | Croatia | General popln. | measured | 1349 | 23.1 (3.3) | 50.3 (5.1) | 67.0 (8.0) | 1349 (100) | 792 (59) | 1958 | 23.5 (14.5, 24.5) | + | 744 | 324 | 96 | 138 | 143 | 116 |
| Copenhagen City Heart Study | 5 | Denmark | Popln. register | measured | 14166 | 25.2 (4.2) | 52.6 (12.0) | 70.0 (10.0) | 6485 (46) | 8374 (59) | 1976 | 16.5 (14.5, 17.5) | ++ | 4719 | 1084 | 658 | 184 | 934 | 150 |
| Research Centre for Prevention and Health (Glostrup Population Studies) | 6 | Denmark | Popln. register | measured | 10135 | 24.9 (4.0) | 48.4 (13.6) | 69.0 (13.0) | 5076 (50) | 5244 (52) | 1983 | 10.5 (5.5, 10.5) | ++ | 1070 | 446 | 268 | 76 | 351 | 59 |
| Finnish Mobile Clinic Survey | 7 | Finland | General popln. | measured | 44798 | 25.3 (4.0) | 42.1 (15.0) | 70.0 (13.0) | 24034 (54) | 14928 (33) | 1970 | 22.5 (20.5, 23.5) | + | 12183 | 6792 | 4167 | 1475 | 2636 | 221 |
| Finrisk Cohort 1977 | 8 | Finland | Popln. register | measured | 12080 | 26.1(4.2) | 43.7(10.9) | 62.0 (10.0) | 5919 (49) | 3624 (30) | 1977 | 17.0 (17.0, 17.0) | ++ | 1889 | 1049 | 725 | 201 | 427 | 75 |
| Finrisk Cohort 1982 | 8 | Finland | Popln. register | measured | 9340 | 26.1(4.3) | 43.9(11.3) | 60.0 (9.0) | 4577 (49) | 2895 (31) | 1982 | 12.0 (12.0, 12.0) | ++ | 825 | 400 | 283 | 64 | 249 | 23 |
| Finrisk Cohort 1987 | 8 | Finland | Popln. register | measured | 6232 | 26.5(4.4) | 44.3(11.3) | 58.0 (9.0) | 2991 (48) | 1745 (28) | 1987 | 7.0 (7.0, 7.0) | ++ | 249 | 134 | 97 | 20 | 56 | 5 |
| Finrisk Cohort 1992 | 8 | Finland | Popln. register | measured | 6011 | 26.3 (4.5) | 44.9 (11.4) | 62.0 (8.0) | 2837 (47) | 1841 (31) | 1992 | 16.9 (16.8, 16.9) | ++ | 376 | 139 | 81 | 23 | 104 | 31 |
| Finrisk Cohort 1997 | 8 | Finland | Popln. register | measured | 7653 | 26.8 (4.5) | 49.8 (12.3) | 68.0 (9.0) | 3849 (50) | 1976 (26) | 1997 | 11.8 (11.8, 11.9) | ++ | 340 | 131 | 71 | 13 | 90 | 50 |
| Helsinki Aging Study | 9 | Finland | Popln. register | measured | 501 | 25.4 (4.2) | 78.8 (4.1) | 85.0 (5.0) | 131 (26) | 37 (8) | 1989 | 9.1 (4.7, 11.0) | ++ | 276 | 130 | 59 | 44 | 43 | 56 |
| Helsinki Businessmen Study | 10 | Finland | Health check-up | measured | 1367 | 26.0 (3.0) | 59.8 (4.1) | 76.0 (7.0) | 1367 (100) | 236 (17) | 1986 | 24.5 (18.0, 24.5) | + | 579 | 150 | 100 | 32 | 166 | 0 |
| Kuopio Ischaemic Heart Disease Study | 11 | Finland | Popln. register | measured | 2655 | 26.9 (3.6) | 53.1 (5.2) | 67.0 (7.0) | 2655 (100) | 848 (32) | 1986 | 21.6 (18.1, 23.6) | ++ | 883 | 417 | 292 | 68 | 236 | 29 |
| North Karelia Project | 12 | Finland | Popln. register | measured | 11157 | 25.9 (4.1) | 40.8 (9.9) | 61(10) | 5453 (49) | 3927 (35) | 1972 | 22.0 (22.0, 22.0) | ++ | 2188 | 1171 | 811 | 203 | 510 | 77 |
| Seven Countries Finland | 4 | Finland | General popln. | measured | 1633 | 23.7 (3.2) | 49.3 (5.6) | 65.0 (8.0) | 1633 (100) | 1022 (63) | 1959 | 23.5 (14.5, 25.5) | + | 888 | 515 | 360 | 79 | 208 | 59 |
| Centre d'Investigations Preventives et Cliniques (IPC), Paris | 13 | France | Health check-up | measured | 220101 | 23.7 (3.5) | 40.7 (11.7) | 61.0 (14.0) | 124983 (57) | 65381 (30) | 1982 | 11.5 (9.5, 14.5) | ++ | 7170 | 1461 | 633 | 356 | 3057 | 200 |
| Data from an Epidemiological Study on the Insulin Resistance Syndrome | 14 | France | Health check-up | measured | 5107 | 24.7 (3.8) | 47.4 (10.0) | 64.0 (9.0) | 2525 (49) | 1127 (22) | 1995 | 14.4 (14.1, 14.7) | + | 199 | 33 | 13 | 10 | 100 | 3 |
| Paris Prospective Study I | 15 | France | Occupational | measured | 7594 | 25.9 (3.3) | 47.2 (2.0) | 62.0 (7.0) | 7594 (100) | 5610 (74) | 1969 | 22.5 (21.5, 24.5) | ++ | 2177 | 620 | 203 | 105 | 917 | 47 |
| Prospective Epidemiological Study of Myocardial Infarction | 16 | France / NI | General popln. | measured | 10598 | 26.6 (3.5) | 54.9 (2.9) | 59.0 (3.0) | 10598 (100) | 2846 (27) | 1992 | 5.2 (5.1, 6.1) | ++ | 242 | 65 | 31 | 9 | 116 | 5 |
| Epidemiologische Studie zu Chancen der Verhütung | 17 | Germany | Health check-up | measured | 9596 | 27.7 (4.3) | 62.1 (6.6) | 68.0 (6.0) | 4324 (45) | 1562 (17) | 2001 | 5.1 (4.9, 5.2) | + | 495 | 162 | 85 | 35 | 220 | 15 |
| Göttingen Risk Incidence and Prevalence Study | 18 | Germany | Occupational | measured | 5996 | 26.3 (3.0) | 47.9 (5.1) | 56.0 (6.0) | 5996 (100) | 2238 (37) | 1982 | 9.8 (9.7, 9.9) | ++ | 352 | 163 | 86 | 23 | 111 | 6 |
| MONICA/KORA Augsburg Survey 1 | 19 | Germany | Popln. register | measured | 918 | 27.6 (3.3) | 54.2 (5.8) | 65.0 (7.0) | 918 (100) | 307 (33) | 1985 | 13.0 (12.8, 13.2) | ++ | 175 | 89 | 52 | 8 | 53 | 13 |
| MONICA/KORA Augsburg Survey 2 | 19 | Germany | Popln. register | measured | 4134 | 27.1 (4.2) | 53.3 (11.8) | 68.0 (8.0) | 2076 (50) | 1001 (24) | 1990 | 7.9 (7.7, 8.1) | ++ | 328 | 145 | 93 | 22 | 105 | 22 |
| MONICA/KORA Augsburg Survey 3 | 19 | Germany | Popln. register | measured | 4689 | 27.1 (4.5) | 50.4 (13.7) | 72.0 (10.0) | 2316 (49) | 1171 (25) | 1995 | 14.0 (13.3, 14.3) | ++ | 666 | 265 | 119 | 64 | 220 | 52 |
| Prospective Cardiovascular Münster Study | 20 | Germany | Occupational | measured | 20391 | 25.6 (3.5) | 43.9 (9.7) | 62.0 (9.0) | 14804 (73) | 7937 (39) | 1983 | 10.0 (7.2, 17.8) | ++ | 1069 | 350 | 151 | 36 | 453 | 57 |
| Study of Health in Pomerania | 21 | Germany | Popln. register | measured | 3760 | 27.7 (4.7) | 53.4 (14.2) | 75.0 (10.0) | 1856 (49) | 1090 (29) | 1999 | 11.3 (10.6, 11.8) | ++ | 555 | 189 | 82 | 51 | 200 | 36 |
| Württemberg Construction Workers Cohort | 22 | Germany | Occupational | measured | 21171 | 26.5 (3.6) | 42.8 (10.8) | 61.0 (6.0) | 21171 (100) | 9432 (53) | 1989 | 17.0 (15.0, 18.6) | + | 2335 | 553 | 309 | 65 | 859 | 124 |
| ATTICA Study | 23 | Greece | General popln. | measured | 1531 | 27.0 (4.4) | 51.2 (10.9) | 72.0 (13.0) | 784 (51) | 603 (39) | 2001 | 5.0 (5.0, 5.0) | + | 50 | 30 | 0 | 0 | 13 | 0 |
| Seven Countries Greece | 4 | Greece | General popln. | measured | 1203 | 23.1 (3.3) | 49.3 (5.6) | 68.0 (8.0) | 1203 (100) | 720 (60) | 1960 | 24.5 (21.5, 24.5) | + | 421 | 182 | 45 | 84 | 111 | 37 |

Notes are at the end of eTable 1, on page 10.

## eTable 1 (continued)

| Study | Ref | Country | Population source | Weight & height assessment | Total participants | Mean (SD) BMI | Mean (SD) age at survey | Mean (SD) age at death | No (%) males | No (%) current smokers | Median baseline survey year | Median (IQR) follow-up time | Death ascertainment | Number of deaths | | | | | |
|---|---|---|---|---|---|---|---|---|---|---|---|---|---|---|---|---|---|---|---|
| | | | | | | | | | | | | | | All-cause mortality | CVD | CHD | Stroke | Cancer | Respiratory disease |
| Reykjavik Study | 24 | Iceland | Popln. register | measured | 18787 | 25.5 (3.9) | 52.8 (8.8) | 76.0 (10.0) | 9091 (48) | 8874 (47) | 1975 | 25.9 (18.7, 33.2) | ++ | 10412 | 4669 | 2799 | 1024 | 3165 | 876 |
| Bezafibrate Infarction Prevention Registry Study | 25 | Israel | GP list | measured | 6355 | 26.7 (3.4) | 60.1 (7.0) | 67.0 (7.0) | 5625 (89) | 730 (11) | 1991 | 3.4 (0.2, 7.5) | ++ | 938 | 422 | 313 | 29 | 130 | 13 |
| Israeli Ischaemic Heart Disease Study | 26 | Israel | Occupational | measured | 10033 | 25.6 (3.3) | 49.3 (6.9) | 68.0 (9.0) | 10033 (100) | 5061 (50) | 1963 | 23.5 (19.5, 23.5) | ++ | 3465 | 1692 | 1025 | 362 | 707 | 149 |
| Glucose Intolerance, Obesity and Hypertension Study | 27 | Israel | Popln. register | measured | 5561 | 25.2 (4.2) | 43.5 (8.5) | 70.0 (10.0) | 2699 (49) | 1735 (31) | 1970 | 29.0 (25.9, 36.0) | ++ | 1945 | 595 | 278 | 134 | 335 | 74 |
| Bruneck Study | 28 | Italy | Popln. register | measured | 919 | 24.9 (3.8) | 58.9 (11.5) | 78.0 (10.0) | 467 (51) | 224 (24) | 1990 | 20.2 (13.7, 20.5) | ++ | 347 | 136 | 65 | 39 | 101 | 51 |
| Cardiovascular Study in the Elderly | 29 | Italy | Popln. register | measured | 3195 | 26.4 (4.3) | 73.6 (5.1) | 82.0 (6.0) | 1257 (39) | 416 (13) | 1984 | 11.1 (6.8, 12.7) | ++ | 1560 | 808 | 149 | 162 | 352 | 57 |
| Occupational Groups (OG), Rome | 30 | Italy | Occupational | measured | 3344 | 26.5 (3.3) | 55.2 (5.3) | 64.0 (6.0) | 3344 (100) | 1674 (50) | 1980 | 10.5 (9.5, 10.5) | + | 400 | 164 | 126 | 30 | 170 | 8 |
| Progetto CUORE (ATENA) | 31 | Italy | Electoral roll | measured | 4957 | 27.2 (4.4) | 50.0 (7.4) | 59.0 (9.0) | 0 (0) | 1977 (40) | 1995 | 6.8 (6.0, 7.5) | ++ | 45 | 12 | 2 | 2 | 22 | 0 |
| Progetto CUORE (EMOFRI) | 31 | Italy | Electoral roll | measured | 400 | 26.6 (4.1) | 54.7 (5.7) | 59.0 (8.0) | 200 (50) | 106 (27) | 1996 | 6.7 (6.6, 7.0) | ++ | 13 | 8 | 0 | 3 | 2 | 0 |
| Progetto CUORE (MATISS83) | 31 | Italy | Electoral roll | measured | 2678 | 28.2 (4.5) | 51.8 (9.6) | 70.0 (9.0) | 1260 (47) | 785 (29) | 1984 | 18.7 (18.5, 18.9) | ++ | 539 | 285 | 50 | 34 | 97 | 15 |
| Progetto CUORE (MATISS87) | 31 | Italy | Electoral roll | measured | 2208 | 28.7 (4.5) | 52.6 (9.5) | 68.0 (8.0) | 982 (44) | 471 (22) | 1987 | 15.6 (15.2, 15.9) | ++ | 286 | 146 | 32 | 11 | 48 | 7 |
| Progetto CUORE (MATISS93) | 31 | Italy | Electoral roll | measured | 1310 | 28.2 (4.6) | 49.8 (9.3) | 62.0 (8.0) | 631 (48) | 341 (26) | 1994 | 8.3 (7.8, 9.2) | ++ | 47 | 23 | 6 | 3 | 7 | 0 |
| Progetto CUORE (MONFRI86) | 31 | Italy | Electoral roll | measured | 1441 | 26.6 (4.1) | 49.3 (8.7) | 65.0 (8.0) | 711 (49) | 459 (32) | 1986 | 16.7 (16.6, 16.8) | ++ | 194 | 79 | 10 | 2 | 42 | 3 |
| Progetto CUORE (MONFRI89) | 31 | Italy | Electoral roll | measured | 1388 | 26.5 (4.3) | 48.7 (8.3) | 62.0 (9.0) | 691 (50) | 392 (28) | 1989 | 13.6 (13.5, 13.6) | ++ | 116 | 52 | 6 | 5 | 17 | 8 |
| Progetto CUORE (MONFRI94) | 31 | Italy | Electoral roll | measured | 1370 | 26.4 (4.3) | 49.1 (8.1) | 59.0 (6.0) | 680 (50) | 365 (27) | 1994 | 8.5 (8.3, 8.6) | ++ | 57 | 22 | 0 | 5 | 7 | 1 |
| Risk Factors and Life Expectancy Pooling Project (ATS_SAR) | 32 | Italy | Electoral roll | measured | 4343 | 26.7 (4.5) | 46.3 (7.8) | 58.0 (6.0) | 2100 (48) | 1220 (32) | 1983 | 8.7 (7.7, 8.7) | + | 128 | 38 | 23 | 8 | 57 | 6 |
| Risk Factors and Life Expectancy Pooling Project (DISCO) | 32 | Italy | Electoral roll | measured | 2062 | 28.2 (4.6) | 50.6 (11.2) | 66.0 (7.0) | 911 (44) | 539 (28) | 1987 | 5.5 (5.5, 8.5) | + | 36 | 17 | 12 | 5 | 12 | 0 |
| Risk Factors and Life Expectancy Pooling Project (GREPCO) | 32 | Italy | Electoral roll | measured | 796 | 24.6 (3.9) | 43.9 (8.3) | 52.0 (9.0) | 0 (0) | 319 (41) | 1980 | 7.9 (7.8, 8.1) | + | 5 | 0 | 0 | 0 | 4 | 0 |
| Risk Factors and Life Expectancy Pooling Project (GUBBIO) | 32 | Italy | Electoral roll | measured | 3408 | 27.1 (4.2) | 55.0 (13.2) | 73.0 (10.0) | 1515 (44) | 1161 (34) | 1984 | 8.4 (7.4, 9.4) | + | 239 | 107 | 69 | 29 | 95 | 9 |
| Risk Factors and Life Expectancy Pooling Project (MICOL) | 32 | Italy | Electoral roll | measured | 19911 | 26.4 (4.0) | 50.8 (9.7) | 62.0 (8.0) | 11244 (56) | 6271 (32) | 1986 | 5.9 (5.5, 6.8) | + | 561 | 180 | 133 | 35 | 256 | 18 |
| Risk Factors and Life Expectancy Pooling Project (MONICA) | 32 | Italy | Electoral roll | measured | 3661 | 26.5 (4.2) | 49.3 (9.0) | 59.0 (8.0) | 1830 (50) | 1242 (34) | 1986 | 6.5 (2.3, 8.5) | + | 100 | 38 | 28 | 8 | 45 | 4 |
| Risk Factors and Life Expectancy Pooling Project (NFR) | 32 | Italy | Electoral roll | measured | 3339 | 26.5 (3.3) | 55.2 (5.3) | 64.0 (6.0) | 3339 (100) | 1672 (50) | 1980 | 10.2 (9.8, 10.4) | + | 400 | 163 | 126 | 29 | 171 | 7 |
| Risk Factors and Life Expectancy Pooling Project (OB43) | 32 | Italy | Electoral roll | measured | 3704 | 26.6 (4.4) | 47.0 (7.8) | 57.0 (6.0) | 1793 (48) | 1290 (36) | 1984 | 7.5 (5.4, 8.1) | + | 81 | 28 | 19 | 8 | 36 | 4 |
| Risk Factors and Life Expectancy Pooling Project (RF2) | 32 | Italy | Electoral roll | measured | 5538 | 26.1 (4.3) | 44.2 (9.3) | 59.0 (7.0) | 2603 (47) | 2006 (36) | 1978 | 13.7 (12.8, 13.8) | + | 340 | 105 | 76 | 21 | 151 | 8 |
| Seven Countries Italy | 4 | Italy | Occupational | measured | 2460 | 25.6 (3.8) | 48.7 (5.2) | 66.0 (8.0) | 2460 (100) | 1531 (62) | 1959 | 24.5 (16.5, 24.5) | + | 1081 | 481 | 220 | 135 | 334 | 67 |
| Vicenza Thrombophilia and Athrosclerosis Project | 33 | Italy | Census list | measured | 9366 | 25.3 (4.0) | 51.4 (8.2) | 73.0 (12.0) | 4277 (46) | 2452 (26) | 1995 | 3.3 (2.3, 4.4) | ++ | 106 | 31 | 14 | 5 | 52 | 3 |
| Consultation Bureau Project | 34 | Netherlands | General popln. | measured | 48891 | 24.6 (3.4) | 38.8 (4.3) | 50.0 (6.0) | 23330 (48) | 25407 (52) | 1977 | 12.5 (10.5, 13.5) | + | 1288 | 393 | 232 | 72 | 556 | 25 |
| Hoorn Study | 35 | Netherlands | Popln. register | measured | 2462 | 26.5 (3.6) | 61.7 (7.3) | 74.0 (8.0) | 1130 (46) | 767 (31) | 1991 | 13.6 (11.4, 14.1) | + | 610 | 229 | 36 | 40 | 173 | 33 |
| Longitudinal Aging Study Amsterdam | 36 | Netherlands | Popln. register | measured | 2607 | 26.8 (4.1) | 70.4 (8.7) | 81.0 (7.0) | 1277 (49) | 657 (25) | 1993 | 9.8 (6.8, 10.2) | + | 976 | 0 | 0 | 0 | 0 | 0 |
| Monitoring of CVD Risk Factors Project | 37 | Netherlands | General popln. | measured | 24831 | 25.2 (4.0) | 42.4 (10.3) | 62.0 (8.0) | 11640 (47) | 10920 (44) | 1989 | 16.7 (15.4, 18.0) | + | 2099 | 594 | 272 | 113 | 952 | 116 |
| Prevention of Renal and Vascular End Stage Disease Study | 38 | Netherlands | General popln. | measured | 8488 | 26.1 (4.2) | 49.3 (12.7) | 71.0 (10.0) | 4248 (50) | 2901 (34) | 1998 | 12.7 (12.4, 12.9) | ++ | 833 | 239 | 104 | 44 | 387 | 47 |

Notes are at the end of eTable 1, on page 10.

## eTable 1 (continued)

| Study | Ref | Country | Population source | Weight & height assessment | Total participants | Mean (SD) BMI | Mean (SD) age at survey | Mean (SD) age at death | No (%) males | No (%) current smokers | Median baseline survey year | Median (IQR) follow-up time | Death ascertainment | Number of deaths | | | | | |
|---|---|---|---|---|---|---|---|---|---|---|---|---|---|---|---|---|---|---|---|
| | | | | | | | | | | | | | | All-cause mortality | CVD | CHD | Stroke | Cancer | Respiratory disease |
| Seven Countries Netherlands | 4 | Netherlands | General popln. | measured | 876 | 24.0 (2.7) | 49.4 (5.5) | 67.0 (8.0) | 876 (100) | 652 (74) | 1959 | 24.5 (17.5, 24.5) | + | 425 | 221 | 153 | 31 | 155 | 20 |
| The Rotterdam Study | 39 | Netherlands | Popln. register | measured | 6315 | 26.3 (3.7) | 68.7 (8.4) | 81.0 (8.0) | 2610 (41) | 1162 (20) | 1992 | 11.9 (8.9, 12.9) | ++ | 2423 | 937 | 146 | 245 | 653 | 146 |
| The Rotterdam Study II | 39 | Netherlands | Popln. register | measured | 2659 | 27.2 (4.1) | 64.6 (7.7) | 77.0 (9.0) | 1200 (45) | 608 (23) | 2000 | 10.0 (9.2, 10.5) | ++ | 440 | 145 | 19 | 40 | 161 | 31 |
| The Rotterdam Study III | 39 | Netherlands | Popln. register | measured | 3566 | 27.8 (4.6) | 56.9 (6.8) | 65.0 (10.0) | 1544 (43) | 960 (27) | 2007 | 3.6 (2.8, 4.2) | ++ | 63 | 20 | 2 | 4 | 34 | 3 |
| Cohort of Norway (FINNMARK) | 40 | Norway | Popln. register | measured | 6231 | 27.7 (4.5) | 59.9 (9.9) | 72.0 (8.0) | 2971 (48) | 2243 (36) | 2002 | 7.5 (7.5, 7.5) | + | 514 | 182 | 90 | 46 | 182 | 42 |
| Cohort of Norway (HUBRO) | 40 | Norway | Popln. register | measured | 16796 | 25.7 (4.1) | 52.0 (14.1) | 75.0 (10.0) | 7484 (45) | 4661 (28) | 2001 | 8.5 (8.5, 9.5) | + | 1178 | 333 | 130 | 99 | 463 | 88 |
| Cohort of Norway (OPPHED) | 40 | Norway | Popln. register | measured | 10239 | 26.9 (4.2) | 53.1 (12.7) | 74.0 (11.0) | 4739 (46) | 3132 (31) | 2001 | 8.5 (8.5, 8.5) | + | 763 | 251 | 111 | 69 | 281 | 66 |
| Cohort of Norway (OSLO2) | 40 | Norway | General popln. | measured | 6383 | 26.4 (3.4) | 69.2 (6.3) | 77.0 (5.0) | 6383 (100) | 1355 (21) | 2000 | 9.5 (9.5, 9.5) | + | 1540 | 484 | 217 | 114 | 612 | 125 |
| Cohort of Norway (TROMS) | 40 | Norway | Popln. register | measured | 2133 | 27.3 (4.2) | 52.1 (12.4) | 67.0 (7.0) | 950 (45) | 725 (34) | 2002 | 7.5 (7.5, 7.5) | + | 95 | 38 | 23 | 4 | 40 | 4 |
| Norwegian Counties Study (NCS1) | 41 | Norway | Popln. register | measured | 24630 | 24.9 (3.5) | 42.4 (4.3) | 54.0 (6.0) | 12194 (50) | 10650 (43) | 1976 | 16.1 (15.7, 16.4) | + | 1519 | 604 | 423 | 70 | 566 | 50 |
| Norwegian Counties Study (NCS2) | 41 | Norway | Popln. register | measured | 13306 | 24.7 (3.4) | 42.3 (4.4) | 55.0 (6.0) | 6819 (51) | 5167 (39) | 1975 | 17.2 (17.0, 17.6) | + | 859 | 319 | 221 | 34 | 334 | 22 |
| Norwegian Counties Study (NCS3) | 41 | Norway | Popln. register | measured | 10345 | 25.2 (3.8) | 42.2 (4.4) | 55.0 (6.0) | 5411 (52) | 5681 (56) | 1974 | 18.1 (18.0, 18.4) | + | 1092 | 547 | 347 | 94 | 295 | 34 |
| Oslo Study | 42 | Norway | Popln. register | measured | 17786 | 24.6 (2.9) | 43.9 (5.6) | 66.0 (8.0) | 17786 (100) | 9937 (56) | 1973 | 29.5 (25.1, 30.0) | + | 6203 | 2819 | 1772 | 393 | 2076 | 346 |
| Tromsø Study | 43 | Norway | Household listings | measured | 26120 | 24.4 (3.7) | 42.9 (15.6) | 74.0 (10.0) | 12400 (47) | 10607 (48) | 1987 | 21.9 (13.9, 22.2) | + | 3461 | 1280 | 584 | 287 | 993 | 254 |
| Edinburgh Artery Study | 44 | Scotland | GP list | measured | 1591 | 25.6 (3.9) | 64.9 (5.7) | 78.0 (7.0) | 808 (51) | 403 (25) | 1988 | 18.2 (10.5, 20.8) | ++ | 916 | 350 | 184 | 96 | 274 | 106 |
| Prospective Study of Pravastatin in the Elderly at Risk | 45 | Scotland/Ireland/ Netherland | Health check-up | measured | 5802 | 26.8 (4.2) | 75.3 (3.3) | 78.0 (4.0) | 2804 (48) | 1557 (27) | 1998 | 3.3 (3.0, 3.5) | ++ | 604 | 292 | 216 | 36 | 206 | 0 |
| Seven Countries Serbia | 4 | Serbia | Occupational | measured | 1562 | 24.5 (3.6) | 48.4 (5.9) | 66.0 (8.0) | 1562 (100) | 804 (51) | 1963 | 24.5 (17.5, 25.5) | + | 713 | 369 | 135 | 130 | 166 | 83 |
| Diet and Risk of Cardiovascular Disease in Spain | 46 | Spain | General popln. | measured | 3100 | 26.1 (4.4) | 39.5 (11.5) | 62.0 (9.0) | 1502 (48) | 1228 (41) | 1991 | 19.4 (18.9, 19.5) | + | 208 | 46 | 23 | 10 | 99 | 16 |
| Zaragoza study | 47 | Spain | Popln. register | measured | 2837 | 28.8 (5.0) | 58.9 (11.7) | 61.0 (5.0) | 1175 (41) | 470 (18) | 1994 | 5.1 (5.1, 5.1) | ++ | 24 | 24 | 15 | 9 | 0 | 0 |
| Apolipoprotein Related Mortality Risk Study | 48 | Sweden | Health check-up | measured | 58235 | 24.7 (3.7) | 45.9 (10.4) | 66.0 (12.0) | 33496 (58) | NA | 1989 | 13.3 (10.8, 15.3) | + | 3794 | 1380 | 784 | 262 | 1517 | 155 |
| Cohort of Swedish Men | 49 | Sweden | Popln. register | self-reported | 43157 | 25.8 (3.4) | 60.0 (9.7) | 73.7 (8.0) | 43157 (100) | 10494 (25) | 1998 | 10.0 (10.0, 10.0) | + | 6017 | 2325 | 1291 | 438 | 1559 | 269 |
| Göteborg 1913 Study | 50 | Sweden | Popln. register | measured | 789 | 25.0 (3.2) | 54.1 (1.8) | 74.0 (8.0) | 789 (100) | 423 (54) | 1967 | 25.4 (17.4, 30.5) | ++ | 556 | 293 | 170 | 58 | 141 | 27 |
| Göteborg 1933 Study | 50 | Sweden | Popln. register | measured | 753 | 25.9 (3.4) | 50.6 (0.2) | 58.0 (4.0) | 753 (100) | 269 (36) | 1984 | 12.8 (12.7, 13.0) | ++ | 87 | 28 | 19 | 0 | 27 | 4 |
| Göteborg 1943 Study | 50 | Sweden | Popln. register | measured | 793 | 26.2 (3.4) | 50.0 (0.0) | 56.0 (3.0) | 793 (100) | 242 (31) | 1993 | 10.0 (9.8, 10.6) | ++ | 28 | 6 | 3 | 1 | 16 | 0 |
| MONICA Göteborg Study | 19 | Sweden | Popln. register | measured | 4278 | 25.0 (3.9) | 47.0 (10.6) | 65.0 (9.0) | 2060 (48) | 1259 (30) | 1990 | 13.0 (8.9, 17.7) | ++ | 315 | 108 | 70 | 14 | 134 | 18 |
| Malmö Prevention Project | 51 | Sweden | Popln. screening | measured | 32683 | 24.6 (3.6) | 45.7 (7.4) | 69.0 (9.0) | 21929 (67) | 14540 (45) | 1980 | 29.2 (23.9, 31.4) | ++ | 10156 | 3677 | 2224 | 605 | 3778 | 615 |
| Prospective Study of Women in Gothenburg | 52 | Sweden | Popln. register | measured | 1460 | 24.1 (3.8) | 46.9 (6.3) | 77.0 (10.0) | 0 (0) | 593 (41) | 1969 | 37.3 (27.2, 40.7) | + | 908 | 375 | 151 | 111 | 234 | 24 |
| Swedish Mammography Cohort | 53 | Sweden | Popln. register | self-reported | 36213 | 25.1 (4.0) | 61.7 (9.2) | 74.3 (8.0) | 0 (0) | 8280 (23) | 1998 | 10.0 (10.0, 10.0) | + | 3182 | 988 | 412 | 269 | 1024 | 123 |
| Uppsala Longitudinal Study of Adult Men | 54 | Sweden | General popln. | measured | 2322 | 25.0 (3.2) | 49.6 (0.6) | 76.0 (12.0) | 2322 (100) | 1177 (68) | 1972 | 32.0 (22.2, 36.2) | ++ | 1582 | 716 | 422 | 116 | 534 | 85 |
| Women's Lifestyle and Health Study | 55 | Sweden | Popln. register | self-reported | 47177 | 23.5 (3.6) | 40.4 (5.8) | 52.1 (6.6) | 0 (0) | 10101 (21) | 1991 | 15.0 (14.0, 15.0) | + | 1080 | 147 | 61 | 49 | 653 | 15 |
| Turkish Adult Risk Factor Study | 56 | Turkey | Household listings | measured | 3810 | 26.7 (5.1) | 44.5 (13.7) | 70.0 (12.0) | 1887 (50) | 1366 (36) | 1990 | 19.5 (12.4, 20.0) | + | 564 | 309 | 199 | 62 | 44 | 6 |
| British Regional Heart Study | 57 | UK | GP list | measured | 7729 | 25.5 (3.2) | 50.2 (5.8) | 68.0 (8.0) | 7729 (100) | 4150 (54) | 1979 | 24.5 (19.1, 24.9) | + | 2954 | 1391 | 997 | 207 | 926 | 234 |
| British Union Provident Association Study | 58 | UK | GP list | measured | 21501 | 25.1 (2.8) | 47.5 (7.7) | 68.0 (9.0) | 21501 (100) | 8487 (39) | 1978 | 23.7 (21.1, 25.4) | + | 3817 | 1693 | 1157 | 272 | 1441 | 189 |
| British Women's Heart and Health Study | 59 | UK | Popln. register | measured | 3957 | 27.6 (5.0) | 68.8 (5.5) | 78.0 (6.0) | 0 (0) | 454 (11) | 2000 | 11.0 (10.4, 11.5) | ++ | 814 | 273 | 128 | 83 | 284 | 97 |
| Caerphilly Prospective Study | 60 | UK | Electoral roll | measured | 2472 | 26.2 (3.6) | 52.2 (4.5) | 62.0 (5.0) | 2472 (100) | 1370 (55) | 1981 | 13.0 (13.0, 13.0) | ++ | 490 | 266 | 213 | 26 | 150 | 33 |
| Hertfordshire Cohort Study | 61 | UK | General popln. | measured | 2987 | 27.4 (4.4) | 66.1 (2.9) | 72.0 (4.0) | 1571 (53) | 378 (13) | 2001 | 8.9 (7.8, 9.9) | + | 308 | 79 | 40 | 10 | 168 | 26 |

Notes are at the end of eTable 1, on page 10.

# eTable 1 (continued)

| Study | Ref | Country | Population source | Weight & height assessment | Total participants | Mean (SD) BMI | Mean (SD) age at survey | Mean (SD) age at death | No (%) males | No (%) current smokers | Median baseline survey year | Median (IQR) follow-up time | Death ascertainment | Number of deaths: All-cause mortality | CVD | CHD | Stroke | Cancer | Respiratory disease |
|---|---|---|---|---|---|---|---|---|---|---|---|---|---|---|---|---|---|---|---|
| Lower Extremity Arterial Disease Event Reduction Trial | 62 | UK | Popln. register | measured | 1544 | 26.2 (3.8) | 68.2 (8.7) | 74.0 (8.0) | 1544 (100) | 587 (38) | 1997 | 4.4 (3.5, 4.9) | ++ | 393 | 210 | 142 | 30 | 95 | 55 |
| Midspan Family Study | 63 | UK | General popln. | measured | 2317 | 26.2 (4.6) | 45.1 (6.2) | 58.0 (7.0) | 1039 (45) | 584 (25) | 1996 | 15.4 (15.2, 15.6) | + | 135 | 26 | 17 | 3 | 62 | 9 |
| The Million Women Study | 64 | UK | Popln. screening | self-reported | 1165552 | 26.3 (4.7) | 56.8 (4.6) | 67.4 (6.1) | 0 (0) | 226637 (21) | 1998 | 13.9 (13.1, 14.8) | ++ | 101340 | 21748 | 9759 | 5744 | 54692 | 8818 |
| MRC Study of Older People | 65 | UK | GP list | measured | 13026 | 26.2 (4.3) | 80.2 (3.7) | 86.0 (5.0) | 5194 (40) | 1547 (12) | 1996 | 8.1 (4.3, 10.5) | + | 8533 | 3878 | 1853 | 1169 | 1689 | 1290 |
| Midspan Collaborative Study | 66 | UK | Occupational | measured | 7000 | 25.1 (3.2) | 47.5 (7.2) | 65.0 (8.0) | 5998 (86) | 3889 (56) | 1971 | 22.5 (20.5, 23.5) | + | 2069 | 1044 | 752 | 181 | 665 | 149 |
| Northwick Park Heart Study I | 67 | UK | Occupational | measured | 1508 | 25.5 (3.0) | 52.5 (6.7) | 72.0 (8.0) | 1508 (100) | 771 (51) | 1976 | 25.1 (18.8, 26.8) | ++ | 673 | 315 | 228 | 0 | 214 | 0 |
| Northwick Park Heart Study II | 67 | UK | GP list | measured | 3013 | 26.4 (3.5) | 56.6 (3.5) | 66.0 (5.0) | 3013 (100) | 1122 (37) | 1991 | 8.4 (7.2, 9.0) | ++ | 473 | 144 | 65 | 27 | 252 | 29 |
| Renfew and Paisley Study | 68 | UK | General popln. | measured | 15389 | 25.8 (4.0) | 54.3 (5.6) | 66.0 (7.0) | 7054 (46) | 7881 (51) | 1974 | 16.5 (15.5, 17.5) | + | 4440 | 2291 | 1567 | 476 | 1402 | 317 |
| Scottish Heart Health Study | 60 | UK | GP list | measured | 17365 | 26.0 (4.4) | 50.1 (8.4) | 62.0 (8.0) | 8500 (49) | 7781 (45) | 1986 | 10.0 (10.0, 10.0) | ++ | 1530 | 640 | 442 | 101 | 592 | 103 |
| Speedwell Study | 69 | UK | GP list | measured | 2270 | 25.7 (3.3) | 54.9 (4.4) | 66.0 (6.0) | 2270 (100) | 1062 (47) | 1980 | 16.8 (15.2, 17.7) | ++ | 662 | 343 | 249 | 55 | 228 | 47 |
| Thrombosis Prevention Trial | 70 | UK | GP list | measured | 22712 | 26.6 (3.5) | 56.0 (6.8) | 67.0 (7.0) | 22712 (100) | 7189 (32) | 1990 | 11.4 (10.2, 12.8) | ++ | 3098 | 1267 | 895 | 183 | 1413 | 190 |
| UK Biobank | 71 | UK | General popln. | measured | 499443 | 27.4(4.8) | 57.8(8.0) | 64.5 (6.9) | 227517(46) | 52528(11) | 2009 | 4.8 (4.1, 5.5) | + | 7916 | 1699 | 973 | 296 | 4755 | 387 |
| United Kingdom Heart Disease Prevention Project | 72 | UK | Occupational | measured | 13846 | 25.3 (3.1) | 50.7 (5.7) | 66.0 (8.0) | 13846 (100) | 6568 (47) | 1972 | 20.5 (17.5, 23.5) | + | 4022 | 2008 | 1499 | 256 | 1323 | 286 |
| West of Scotland Coronary Prevention Study | 73 | UK | Popln. screening | measured | 6595 | 26.0 (3.2) | 55.2 (5.5) | 67.0 (10.0) | 6595 (100) | 2907 (44) | 1990 | 4.8 (4.3, 5.3) | ++ | 241 | 123 | 102 | 10 | 0 | 0 |
| Whitehall I Study | 74 | UK | Occupational | measured | 18863 | 24.7 (3.0) | 51.6 (6.7) | 65.0 (7.0) | 18863 (100) | 7855 (42) | 1968 | 15.5 (15.5, 15.5) | + | 3491 | 1846 | 1357 | 221 | 1070 | 284 |
| Whitehall II Study | 75 | UK | Occupational | measured | 10279 | 24.6 (3.5) | 45.0 (6.1) | 83.0 (5.0) | 6880 (67) | 2647 (26) | 1987 | 17.5 (16.9, 18.3) | + | 572 | 119 | 90 | 9 | 155 | 12 |
| European Prospective Investigation into Cancer and Nutrition | 76 | Europe | General popln.** | measured | 367731 | 25.9 (4.3) | 51.7 (10.4) | 64.1 (10.5) | 129349 (35) | 96296 (27) | 1995 | 12.6 (11.5, 14.0) | + | 25347 | 6516 | 3042 | 1498 | 9857 | 1108 |
| **Subtotal (115 studies)** | | | | | **3390101** | **26.0 (3.9)** | **52.1 (8.1)** | **67.1 (8.0)** | **1136198 (33.5)** | **812698 (23.8)** | **1987*** | **12.6 (11.2, 13.7)** | | **305606** | **100287** | **54263** | **21430** | **122606** | **19976** |
| **North America** | | | | | | | | | | | | | | | | | | | |
| Nova Scotia Health Survey | 77 | Canada | GP list | measured | 1814 | 27.5 (5.3) | 55.1 (15.5) | 78.0 (10.0) | 889 (49) | 492 (27) | 1995 | 9.8 (9.5, 9.9) | ++ | 268 | 71 | 41 | 11 | 0 | 0 |
| Quebec Cardiovascular Study | 78 | Canada | Popln. register | measured | 4080 | 25.8 (3.8) | 49.5 (8.3) | 68.0 (9.0) | 4080 (100) | 2400 (59) | 1974 | 21.8 (19.0, 27.8) | ++ | 1143 | 218 | 82 | 28 | 0 | 0 |
| Agricultural Health Study | 79 | USA | Occupational | self-reported | 69448 | 26.9 (4.5) | 47.3 (12.5) | 67.6 (11.0) | 40246 (58) | 8681 (13) | 1995 | 10.0 (9.0, 11.0) | + | 3556 | 1117 | 661 | 183 | 1370 | 218 |
| Air Force/Texas Coronary Atherosclerosis Prevention Study | 80 | USA | Popln. screening | measured | 6605 | 26.9 (3.1) | 58.2 (7.2) | 66.0 (7.0) | 5608 (85) | 652 (12) | 1992 | 5.2 (4.8, 5.9) | ++ | 157 | 42 | 7 | 1 | 82 | 0 |
| NIH-AARP Diet and Health Study | 81 | USA | Popln. register | self-reported | 548453 | 27.1 (4.8) | 62.1 (5.4) | 69.4 (5.4) | 330669 (60) | 65318 (12) | 1996 | 10.0 (9.0, 10.0) | + | 63876 | 19576 | 11608 | 2508 | 25033 | 5277 |
| Atherosclerosis Risk in Communities Study | 82 | USA | Household listings | measured | 15726 | 27.7 (5.4) | 54.7 (5.8) | 66.0 (7.0) | 7055 (45) | 4506 (29) | 1988 | 14.1 (13.3, 14.9) | ++ | 2360 | 908 | 465 | 119 | 863 | 166 |
| Breast Cancer Detection Demonstration Project | 83 | USA | Popln. screening | self-reported | 46427 | 25.1 (4.6) | 62.1 (8.0) | 74.3 (7.2) | 0 (0) | 5811 (13) | 1988 | 17.0 (14.0, 18.0) | ++ | 8495 | 2710 | 1236 | 631 | 3131 | 665 |
| CLUE II | 84 | USA | Popln. register | self-reported | 21417 | 26.3 (4.8) | 51.3 (14.9) | 71.7 (10.3) | 8935 (42) | 3599 (17) | 1989 | 14.0 (10.0, 17.0) | ++ | 4578 | 1250 | 742 | 192 | 1161 | 321 |
| California Teachers Study | 85 | USA | Occupational | self-reported | 124334 | 24.9 (5.1) | 52.7 (13.8) | 71.7 (10.4) | 0 (0) | 6321 (5) | 1995 | 9.0 (9.0, 9.0) | + | 5580 | 1607 | 733 | 420 | 2564 | 426 |
| Cancer Prevention Study I | 86 | USA | General popln. | self-reported | 808662 | 24.7 (3.8) | 52.7 (10.5) | 69.6 (11.4) | 275049 (34) | 290611 (36) | 1959 | 12.9 (11.9, 12.9) | ++ | 127417 | 75396 | 25490 | 14888 | 29666 | 3674 |
| Cancer Prevention Study II | 86 | USA | General popln. | self-reported | 957598 | 25.1 (4.1) | 56.4 (10.6) | 79.0 (10.2) | 364792 (38) | 225837 (24) | 1982 | 28.2 (17.4, 28.3) | ++ | 464055 | 187302 | 95853 | 33830 | 128649 | 43577 |
| Cardiovascular Health Study (CHS1) | 87 | USA | Health insurance list | measured | 5155 | 26.4 (4.5) | 72.7 (5.3) | 83.0 (6.0) | 2215 (43) | 598 (12) | 1989 | 13.3 (8.0, 15.4) | ++ | 3086 | 1232 | 804 | 265 | 726 | 328 |
| Cardiovascular Health Study (CHS2) | 87 | USA | Health insurance list | measured | 679 | 28.9 (5.6) | 72.8 (5.5) | 81.0 (6.0) | 252 (37) | 99 (15) | 1993 | 12.1 (7.7, 12.2) | ++ | 327 | 132 | 85 | 34 | 80 | 31 |
| Charleston Heart Study | 88 | USA | Household listings | measured | 2245 | 25.4 (4.9) | 50.2 (11.1) | 74.0 (12.0) | 1065 (47) | 1226 (55) | 1961 | 26.3 (14.6, 37.9) | ++ | 1697 | 904 | 491 | 183 | 348 | 117 |
| Chicago Heart Association Detection Project In Industry | 89 | USA | Occupational | measured | 34244 | 25.6 (4.1) | 40.7 (12.8) | 72.0 (12.0) | 19892 (58) | 14241 (42) | 1971 | 32.0 (28.2, 33.2) | ++ | 11612 | 4820 | 3000 | 786 | 3630 | 904 |
| Established Populations for the Epidemiologic Study of the Elderly - East Boston | 90 | USA | Popln. register | self-reported | 1174 | 27.0 (4.8) | 77.4 (4.4) | 81.0 (5.0) | 432 (37) | 125 (11) | 1988 | 4.9 (4.6, 5.3) | + | 251 | 115 | 32 | 7 | 38 | 34 |

Notes are at the end of eTable 1, on page 10.

# eTable 1 (continued)

| Study | Ref | Country | Population source | Weight & height assessment | Total participants | Mean (SD) BMI | Mean (SD) age at survey | Mean (SD) age at death | No (%) males | No (%) current smokers | Median baseline survey year | Median (IQR) follow-up time | Death ascertainment | Number of deaths: All-cause mortality | CVD | CHD | Stroke | Cancer | Respiratory disease |
|---|---|---|---|---|---|---|---|---|---|---|---|---|---|---|---|---|---|---|---|
| Established Populations for the Epidemiologic Study of the Elderly - Iowa | 90 | USA | Popln. register | measured | 1784 | 26.7 (4.6) | 78.2 (4.8) | 88.0 (6.0) | 623 (35) | 88 (5) | 1988 | 9.9 (5.5, 15.1) | + | 1625 | 751 | 351 | 152 | 186 | 212 |
| Established Populations for the Epidemiologic Study of the Elderly - New Haven | 90 | USA | Popln. register | self-reported | 878 | 25.5 (4.6) | 77.9 (4.8) | 82.0 (5.0) | 364 (41) | 118 (13) | 1988 | 4.3 (4.0, 4.5) | + | 213 | 124 | 11 | 10 | 12 | 17 |
| Established Populations for the Epidemiologic Study of the Elderly - North Carolina | 90 | USA | Popln. register | self-reported | 1671 | 26.9 (5.3) | 77.4 (4.7) | 85.0 (6.0) | 594 (36) | 206 (12) | 1992 | 7.3 (6.1, 11.4) | + | 1075 | 481 | 204 | 109 | 173 | 113 |
| Framingham Offspring Study | 91 | USA | | measured | 3398 | 27.4 (5.0) | 54.2 (9.7) | 70.0 (9.0) | 1547 (46) | 2 (18) | 1992 | 12.1 (11.1, 13.2) | ++ | 322 | 45 | 20 | 14 | 148 | 0 |
| Health Professionals Follow-up Study | 92 | USA | Occupational | self-reported | 50321 | 25.5 (3.2) | 54.8 (9.9) | 77.0 (10.0) | 50321 (100) | 4799 (10) | 1986 | 20.2 (17.4, 20.3) | ++ | 14596 | 5588 | 3577 | 848 | 4745 | 960 |
| Honolulu Heart Program | 93 | USA | Military service registration list | measured | 7997 | 23.9 (3.1) | 54.4 (5.6) | 73.0 (9.0) | 7997 (100) | 3497 (44) | 1966 | 27.5 (19.5, 28.5) | ++ | 3433 | 1084 | 558 | 362 | 1177 | 263 |
| Iowa Women's Health Study | 94 | USA | Driver's license register | self-reported | 41597 | 26.1 (4.9) | 62.2 (4.2) | 74.5 (6.1) | 0 (0) | 6153 (15) | 1986 | 19.0 (16.0, 19.0) | + | 11906 | 4298 | 2236 | 886 | 4088 | 1207 |
| Lipid Research Clinics Follow-up Study | 95 | USA | Health check-up | measured | 8789 | 26.0 (4.3) | 47.0 (12.1) | 69.0 (12.0) | 4691 (53) | 3117 (35) | 1973 | 18.5 (17.5, 19.5) | ++ | 1461 | 715 | 465 | 117 | 343 | 44 |
| Minnesota Heart Health Project | 96 | USA | General popln. | measured | 17970 | 26.0 (4.8) | 45.4 (14.5) | 67.0 (11.0) | 8272 (46) | 5252 (29) | 1984 | 6.5 (3.5, 8.5) | + | 586 | 250 | 153 | 34 | 188 | 42 |
| Minnesota Heart Survey | 97 | USA | General popln. | measured | 9930 | 26.3 (4.9) | 44.6 (13.2) | 65.0 (10.0) | 4662 (47) | 2859 (29) | 1985 | 4.5 (3.5, 8.5) | ++ | 327 | 118 | 72 | 18 | 130 | 15 |
| Multi-Ethnic Study of Atherosclerosis | 98 | USA | General popln. | measured | 6792 | 28.3 (5.5) | 62.2 (10.2) | 75.0 (10.0) | 3205 (47) | 1015 (15) | 2001 | 8.6 (8.4, 8.6) | ++ | 517 | 120 | 74 | 21 | 0 | 0 |
| Multiple Risk Factor Intervention Trial | 99 | USA | Popln. screening | measured | 3878 | 27.8 (3.4) | 46.6 (6.0) | 56.0 (6.0) | 3878 (100) | 2387 (62) | 1974 | 11.3 (10.8, 11.7) | ++ | 290 | 162 | 114 | 11 | 76 | 12 |
| NHLBI Framingham Heart Study | 100 | USA | General popln. | measured | 5198 | 25.6 (4.2) | 44.0 (8.6) | 73.0 (11.0) | 2332 (45) | 2980 (57) | 1950 | 33.5 (21.5, 39.5) | ++ | 3257 | 1398 | 844 | 249 | 820 | 0 |
| NYU Women's Health Study | 101 | USA | Occupational | self-reported | 14205 | 24.9 (4.6) | 50.4 (8.7) | 68.1 (9.4) | 0 (0) | 2288 (18) | 1986 | 19.0 (17.0, 20.0) | + | 1548 | 297 | 176 | 38 | 646 | 52 |
| National Health and Nutrition Examination Survey I | 102 | USA | Census list | measured | 14383 | 25.6 (5.1) | 49.0 (15.6) | 74.0 (11.0) | 5805 (40) | 5345 (37) | 1972 | 18.5 (13.5, 19.5) | + | 4589 | 2293 | 1295 | 406 | 1035 | 384 |
| National Health and Nutrition Examination Survey III | 103 | USA | Census list | measured | 16423 | 26.8 (5.4) | 52.3 (18.3) | 78.0 (12.0) | 7721 (47) | 4374 (27) | 1990 | 14.2 (12.3, 16.2) | + | 4853 | 2195 | 1266 | 370 | 1091 | 462 |
| National Health Interview Survey | 104 | USA | Census list | self-reported | 316945 | 27(5.7) | 47.5(17.5) | 74.6 (14.4) | 159950 (44) | 80302 (22) | 2002 | 2(3, 8) | + | 35632 | 9213 | 7173 | 2040 | 8552 | 2069 |
| Nurses' Health Study I | 105 | USA | Occupational | self-reported | 119116 | 23.8 (4.2) | 42.6 (7.2) | 69.0 (10.0) | 0 (0) | 39070 (33) | 1976 | 28.6 (28.5, 28.9) | ++ | 23703 | 5394 | 2400 | 1361 | 10412 | 1718 |
| Nurses' Health Study II | 106 | USA | Occupational | self-reported | 78095 | 24.5 (5.3) | 36.0 (4.1) | 48.7 (6.8) | 0 (0) | 11490 (0) | 1989 | 10.0 (6.0, 16.0) | ++ | 1793 | 181 | 71 | 44 | 813 | 45 |
| Prostate, Lung, Colorectal and Ovarian (PLCO) Cancer Screening Trial | 107 | USA | Occupational | self-reported | 147070 | 27.3 (4.9) | 62.6 (5.4) | 71.7 (6.2) | 72195 (49) | 15632 (11) | 1997 | 9.0 (7.0, 10.0) | ++ | 14682 | 4697 | 2178 | 691 | 5616 | 1291 |
| Physicians' Health Study I & II | 108 | USA | Occupational | self-reported | 28860 | 25.1 (3.0) | 54.8 (9.8) | 72.5 (8.6) | 28860 (100) | 2623 (9) | 1982 | 21.0 (7.0, 23.0) | ++ | 4894 | 1488 | 651 | 296 | 1850 | 242 |
| Puerto Rico Heart Health Program | 109 | USA | Census list | measured | 9799 | 25.1 (4.1) | 54.4 (6.6) | 65.0 (7.0) | 9799 (100) | 5175 (53) | 1967 | 12.0 (12.0, 12.0) | ++ | 1728 | 840 | 376 | 186 | 382 | 56 |
| Rancho Bernardo Study | 110 | USA | Household listings | measured | 5439 | 24.5 (3.4) | 57.2 (15.0) | 79.0 (9.0) | 2490 (46) | 1327 (24) | 1972 | 18.5 (13.5, 19.5) | + | 2172 | 1106 | 617 | 216 | 558 | 208 |
| Seven Countries US | 4 | USA | Occupational | measured | 2556 | 25.5 (3.2) | 49.4 (5.8) | 77.0 (7.0) | 2556 (100) | 1507 (59) | 1958 | 25.0 (16.0, 25.0) | + | 2128 | 698 | 420 | 95 | 298 | 55 |
| Seventh-day Adventist Cohort Study | 111 | USA | Census list | self-reported | 29961 | 24.6 (4.3) | 52.4 (15.5) | 72.8 (9.9) | 12251 (41) | 1112 (4) | 1976 | 12.0 (10.0, 12.0) | ++ | 3850 | 1863 | 944 | 305 | 1054 | 256 |
| Strong Heart Study | 112 | USA | Tribal rolls | measured | 4313 | 30.8 (6.1) | 56.4 (8.1) | 66.0 (9.0) | 1739 (40) | 1418 (33) | 1990 | 12.6 (10.0, 13.6) | ++ | 1454 | 439 | 211 | 70 | 260 | 109 |
| Tecumseh Study | 113 | USA | General popln. | measured | 4426 | 25.8 (4.7) | 47.1 (12.1) | 71.0 (12.0) | 2151 (49) | 1968 (44) | 1960 | 22.5 (17.5, 26.5) | ++ | 1617 | 898 | 489 | 167 | 375 | 114 |
| U.S. Radiologic Technologists (URST) Cohort | 114 | USA | Occupational | self-reported | 87144 | 25.7 (5.0) | 48.9 (9.5) | 63.7 (11.8) | 20129 (23) | 11722 (14) | 1995 | 6.0 (4.0, 6.0) | + | 1556 | 447 | 231 | 66 | 661 | 111 |
| Vitamins and Lifestyle (VITAL) Study | 115 | USA | Popln. register | self-reported | 72885 | 27.4 (5.2) | 61.4 (7.4) | 70.0 (7.2) | 35710 (49) | 6051 (8) | 2001 | 6.0 (5.0, 6.0) | ++ | 3729 | 934 | 525 | 157 | 1754 | 324 |
| Women's Health Initiative (Observational Study) | 116 | USA | General popln. | measured | 91933 | 27.2 (5.7) | 63.6 (7.4) | 61.0 (8.0) | 0 (0) | 5664 (6) | 1996 | 12.0 (8.4, 13.8) | ++ | 12121 | 3342 | 1494 | 878 | 4528 | 0 |
| Women's Health Study | 117 | USA | Occupational | self-reported | 38980 | 26.0 (5.1) | 54.6 (7.0) | 67.1 (8.6) | 0 (0) | 5091 (13) | 1993 | 13.0 (12.0, 14.0) | ++ | 2061 | 355 | 61 | 85 | 802 | 99 |
| Antihypertensive and Lipid-Lowering Treatment to Prevent Heart Attack Trial | 118 | USA/Canada/Puerto Rico/US Virgin Islands | Popln. register | measured | 41944 | 29.7 (5.9) | 66.8 (7.6) | 74.0 (8.0) | 22395 (53) | 9186 (22) | 1994 | 4.7 (3.9, 5.6) | ++ | 5713 | 2688 | 1044 | 467 | 1308 | 0 |
| **Subtotal (48 studies)** | | | | | **3932741** | **26.3 (4.6)** | **55.7 (9.3)** | **71.9 (8.9)** | **1533416 (39.0)** | **874335 (22.2)** | **1988*** | **15.6 (11.9, 16.5)** | | **867889** | **351902** | **171631** | **64855** | **251422** | **66218** |

Notes are at the end of eTable 1, on page 10.

## eTable 1 (continued)

| Study | Ref | Country | Population source | Weight & height assessment | Total participants | Mean (SD) BMI | Mean (SD) age at survey | Mean (SD) age at death | No (%) males | No (%) current smokers | Median baseline survey year | Median (IQR) follow-up time | Death ascertain ment | Number of deaths | | | | | |
|---|---|---|---|---|---|---|---|---|---|---|---|---|---|---|---|---|---|---|---|
| | | | | | | | | | | | | | | All-cause mortality | CVD | CHD | Stroke | Cancer | Respiratory disease |
| **Australia/New Zealand** | | | | | | | | | | | | | | | | | | | |
| 45 and Up Study | 119 | Australia | General popln. | self-reported | 244643 | 27 (4.9) | 62 (11) | 68.5 (10.7) | 115004 (47) | 17745 (7.3) | 2008 | 5.9 (5.7, 6.3) | + | 16719 | 4427 | 2163 | 1039 | 6385 | 1173 |
| Abdominal Aortic Aneurysm Screening Program | 120 | Australia | Electoral roll | measured | 12190 | 26.9 (3.7) | 72.2 (4.4) | 76.0 (5.0) | 12190 (100) | 1330 (11) | 1997 | 3.2 (2.7, 3.6) | + | 973 | 359 | 239 | 60 | 399 | 68 |
| Australian Diabetes, Obesity and Lifestyle Study | 121 | Australia | Census list | measured | 10823 | 27.0 (5.0) | 52.3 (13.9) | 78.0 (11.0) | 4868 (45) | 1752 (17) | 2000 | 12.6 (12.2, 13.1) | + | 1217 | 266 | 138 | 77 | 314 | 64 |
| Australian Longitudinal Study of Ageing | 122 | Australia | Electoral roll | measured | 1487 | 26.1 (4.1) | 77.3 (5.7) | 84.0 (6.0) | 772 (52) | 115 (8) | 1992 | 5.0 (2.0, 7.9) | + | 403 | 176 | 72 | 38 | 62 | 74 |
| Busselton Health Study | 123 | Australia | Electoral roll | measured | 6808 | 24.8 (3.8) | 46.6 (16.8) | 78.0 (11.0) | 3227 (47) | 2116 (31) | 1969 | 24.4 (18.4, 32.7) | + | 2526 | 1297 | 735 | 303 | 644 | 201 |
| Dubbo Study of the Elderly | 124 | Australia | Electoral roll | measured | 2785 | 26.0 (4.2) | 69.1 (6.9) | 80.0 (8.0) | 1225 (44) | 426 (15) | 1989 | 14.1 (8.3, 14.5) | ++ | 1274 | 657 | 419 | 122 | 260 | 129 |
| Health in Men Study | 125 | Australia | Electoral roll | measured | 12190 | 26.9 (3.7) | 72.1 (4.4) | 81.0 (5.0) | 12190 (100) | 1313 (11) | 1997 | 12.6 (8.3, 13.3) | + | 5662 | 1930 | 1149 | 387 | 2047 | 478 |
| National Heart Foundation Risk Factor Prevalence Survey 1989 | 126 | Australia | Electoral roll | measured | 9258 | 25.4 (4.3) | 43.4 (13.5) | 62.0 (12.0) | 4542 (49) | 2230 (24) | 1989 | 8.3 (8.2, 8.5) | + | 372 | 114 | 76 | 17 | 154 | 21 |
| Newcastle Study | 127 | Australia | Electoral roll | measured | 5924 | 26.7 (4.5) | 51.7 (10.5) | 65.0 (8.0) | 2948 (50) | 1361 (23) | 1988 | 8.9 (3.5, 14.3) | + | 514 | 203 | 137 | 33 | 215 | 31 |
| Perth Risk Factors Survey | 128 | Australia | Electoral roll | measured | 10218 | 25.2 (3.9) | 45.2 (12.9) | 69.0 (12.0) | 5288 (52) | 2603 (25) | 1982 | 14.5 (8.5, 19.5) | + | 818 | 266 | 166 | 51 | 277 | 35 |
| Melbourne Collaborative Cohort Study | 129 | Australia/New Zealand/Europe | Electoral roll | measured | 41374 | 26.9 (4.4) | 55.4 (8.7) | 71.2 (8.1) | 16961 (41) | 4663 (11) | 1993 | 15.0 (14.0, 16.0) | + | 4771 | 900 | 514 | 200 | 1737 | 174 |
| Fletcher Challenge Heart and Health Study | 64 | New Zealand | Electoral roll | measured | 10336 | 26.4 (4.2) | 44.2 (14.9) | 69.0 (16.0) | 7449 (72) | 2423 (24) | 1993 | 5.8 (5.4, 6.2) | + | 366 | 168 | 112 | 23 | 134 | 21 |
| **Subtotal (12 studies)** | | | | | **368036** | **26.3 (4.2)** | **55.6 (10.3)** | **73.9 (9.3)** | **186664 (50.7)** | **38077 (10.3)** | **1992-3*** | **8.0 (7.1, 8.8)** | | **35615** | **10763** | **5920** | **2350** | **12628** | **2469** |
| **East Asia** | | | | | | | | | | | | | | | | | | | |
| Anzhen 02 Cohort Study | 127 | China | General popln. | measured | 4152 | 24.0 (3.3) | 47.0 (8.2) | 53.0 (7.0) | 2032 (49) | 864 (21) | 1992 | 3.0 (3.0, 3.0) | + | 19 | 2 | 1 | 1 | 0 | 0 |
| Beijing Anzhen Cohort Study | 127 | China | General popln. | measured | 8336 | 23.9 (3.7) | 53.7 (12.7) | 71.0 (11.0) | 3744 (45) | 2377 (29) | 1991 | 4.3 (4.3, 4.3) | + | 318 | 189 | 58 | 96 | 66 | 11 |
| Beijing Aging Study | 130 | China | General popln. | measured | 2052 | 23.3 (3.9) | 69.5 (8.3) | 77.0 (8.0) | 1015 (49) | 611 (30) | 1992 | 4.8 (4.7, 4.9) | ++ | 401 | 196 | 0 | 83 | 48 | 28 |
| Capital Iron & Steel Company Study | 131 | China | Occupational | measured | 5049 | 23.2 (2.7) | 45.3 (7.9) | 59.0 (9.0) | 5049 (100) | 3666 (74) | 1980 | 12.5 (12.5, 13.5) | + | 324 | 104 | 12 | 64 | 104 | 0 |
| Capital Iron & Steel Company Hospital Cohort | 127 | China | Occupational | measured | 2165 | 24.7 (3.5) | 44.2 (7.4) | 49.0 (8.0) | 1064 (49) | 591 (27) | 1992 | 3.3 (3.2, 3.4) | + | 7 | 1 | 1 | 0 | 3 | 0 |
| China Hypertension Survey Epidemiology Follow-up Study | 132 | China | General popln. | measured | 142974 | 22.7 (3.6) | 55.8 (10.6) | 70.6 (10.9) | 70057 (49) | 50040 (35) | 1991 | 8.2 (8.1, 8.3) | + | 17275 | 8980 | 1241 | 3871 | 3901 | 801 |
| China Prospective Study | 133 | China | Popln. register | measured | 217628 | 21.7 (2.7) | 54.7 (10.1) | 69.3 (10.2) | 217628 (100) | 145990 (67) | 1991 | 14.1 (9.2, 14.2) | + | 48017 | 18810 | 3366 | 10305 | 9933 | 10198 |
| East Beijing Cohort Study | 127 | China | General popln. | measured | 1080 | 23.6 (3.2) | 43.2 (14.9) | 71.0 (13.0) | 524 (49) | 320 (30) | 1980 | 17.1 (9.3, 17.5) | + | 85 | 45 | 9 | 19 | 15 | 2 |
| Fangshan Cohort Study | 134 | China | Census list | measured | 2606 | 24.4 (3.6) | 47.3 (10.0) | 62.0 (12.0) | 871 (33) | 1018 (39) | 1991 | 3.6 (2.7, 3.7) | + | 46 | 22 | 0 | 11 | 8 | 1 |
| Huashan Study | 127 | China | Census/electoral roll | measured | 1852 | 23.4 (3.4) | 53.0 (11.6) | 66.0 (11.0) | 889 (48) | 460 (25) | 1992 | 2.8 (2.7, 3.0) | + | 26 | 12 | 3 | 6 | 4 | 1 |
| Nutrition Intervention Trials - Linxian | 135 | China | General popln. | measured | 29433 | 22.0 (2.5) | 51.9 (8.9) | 69.5 (8.8) | 13123 (45) | 8839 (30) | 1985 | 22.7 (12.6, 24.7) | ++ | 15918 | NA | NA | NA | NA | NA |
| Seven Cities Cohort Study | 127 | China | General popln. | measured | 10690 | 22.7 (3.6) | 53.7 (12.0) | 70.0 (10.0) | 4877 (46) | 3757 (35) | 1987 | 2.7 (2.7, 10.5) | ++ | 859 | 500 | 81 | 183 | 172 | 55 |
| Shanghai Cohort Study | 136 | China | General popln. | self-reported | 18066 | 22.2 (3.0) | 55.3 (5.7) | 69.3 (7.0) | 18074 (100) | 9140 (51) | 1988 | 18.0 (16.1, 18.7) | + | 4972 | 1748 | 449 | 1040 | 1984 | 520 |
| Shanghai Men's Health Study | 137 | China | General popln. | measured | 61339 | 23.7 (3.1) | 55.3 (9.7) | 66.3 (9.4) | 61339 (100) | 35981 (59) | 2004 | 3.1 (2.1, 4.2) | + | 938 | 307 | 98 | 136 | 422 | 44 |
| Shanghai Women's Health Study | 138 | China | Popln. register | measured | 74828 | 24.0 (3.4) | 52.6 (9.1) | 66.2 (8.4) | 0 (0) | 1778 (2) | 1998 | 8.7 (7.9, 9.4) | + | 2882 | 815 | 175 | 450 | 1349 | 67 |
| Six Chinese Cohorts Study | 127 | China | Occupational | measured | 19328 | 21.2 (2.6) | 44.7 (7.0) | 55.0 (8.0) | 10311 (53) | 8900 (46) | 1983 | 9.0 (8.1, 9.1) | + | 912 | 287 | 27 | 120 | 380 | 46 |
| Tianjin Study | 139 | China | NA | measured | 9213 | 23.6 (3.9) | 54.4 (11.9) | 70.0 (11.0) | 4507 (49) | 4692 (51) | 1984 | 6.1 (6.1, 6.1) | + | 1158 | 634 | 85 | 287 | 295 | 97 |
| Yunnan Tin Miner Cohort Study | 140 | China | Occupational | measured | 6571 | 21.6 (2.9) | 55.8 (9.3) | 66.0 (8.0) | 6368 (97) | 4439 (68) | 1992 | 4.5 (4.4, 4.7) | + | 626 | 222 | 11 | 106 | 237 | 61 |
| Guangzhou Occupational Cohort Study | 141 | Hong Kong | Occupational | measured | 20537 | 22.6 (3.0) | 42.4 (6.1) | 54.0 (8.0) | 14029 (68) | 9907 (48) | 1991 | 7.5 (6.7, 8.6) | + | 250 | 67 | 13 | 36 | 127 | 6 |
| Hong Kong Study | 142 | Hong Kong | Health insurance list | measured | 2520 | 22.2 (3.7) | 76.9 (5.2) | 81.0 (6.0) | 1107 (44) | 488 (19) | 1991 | 2.5 (1.5, 3.3) | + | 433 | 141 | 55 | 54 | 104 | 120 |
| Aito Town Study | 127 | Japan | General popln. | measured | 1708 | 22.6 (3.0) | 51.0 (9.1) | 69.0 (9.0) | 740 (43) | 320 (28) | 1981 | 15.2 (15.0, 16.4) | + | 179 | 57 | 14 | 25 | 62 | 14 |

Notes are at the end of eTable 1, on page 10.

# eTable 1 (continued)

| Study | Ref | Country | Population source | Weight & height assessment | Total participants | Mean (SD) BMI | Mean (SD) age at survey | Mean (SD) age at death | No (%) males | No (%) current smokers | Median baseline survey year | Median (IQR) follow-up time | Death ascertainment | Number of deaths | | | | | |
|---|---|---|---|---|---|---|---|---|---|---|---|---|---|---|---|---|---|---|---|
| | | | | | | | | | | | | | | All-cause mortality | CVD | CHD | Stroke | Cancer | Respiratory disease |
| Akabane Study | 127 | Japan | General popln. | measured | 1832 | 22.5 (3.0) | 54.4 (7.8) | 68.0 (8.0) | 812 (44) | 512 (28) | 1985 | 11.0 (10.7, 12.6) | + | 135 | 34 | 6 | 12 | 57 | 5 |
| Civil Service Workers Study | 143 | Japan | Occupational | measured | 9312 | 22.5 (2.7) | 46.7 (4.6) | 52.0 (4.0) | 6228 (67) | 3500 (38) | 1991 | 6.7 (6.6, 6.8) | NA | 98 | 12 | 1 | 2 | 60 | 2 |
| Funagata Study | 144 | Japan | General popln. | measured | 2757 | 23.7 (3.2) | 56.6 (11.5) | 75.0 (10.0) | 1216 (44) | 331 (29) | 1991 | 11.2 (7.3, 12.0) | + | 243 | 76 | 27 | 43 | 105 | 22 |
| Hisayama Study | 145 | Japan | Popln. screening | measured | 1514 | 21.6 (2.7) | 55.9 (10.9) | 75.0 (10.0) | 665 (44) | 652 (43) | 1961 | 24.6 (12.6, 25.1) | ++ | 781 | 289 | 50 | 172 | 190 | 156 |
| Ibaraki Prefectural Health Study | 146 | Japan | General popln. | measured | 97526 | 23.5 (3.1) | 58.8 (10.4) | 75.0 (8.5) | 33322 (34) | 19859 (20) | 1993 | 12.3 (12.1, 12.5) | + | 10955 | NA | NA | NA | NA | NA |
| Ikawa, Kyowa, Noichi Study | 147 | Japan | Popln. screening | measured | 2148 | 23.6 (3.2) | 52.2 (8.4) | 69.0 (9.0) | 951 (44) | 682 (32) | 1976 | 16.5 (15.5, 18.5) | ++ | 340 | 120 | 11 | 58 | 103 | 24 |
| Japan Collaborative Cohort Study | 148 | Japan | General popln. | self-reported | 86520 | 22.8 (3.0) | 57.6 (10.0) | 74.1 (9.4) | 36205 (42) | 20694 (24) | 1989 | 14.3 (10.4, 15.0) | + | 12820 | 3964 | 803 | 1833 | 4853 | 1443 |
| Japan Public Health Center–based Prospective Study 1 | 149 | Japan | Residential registry | self-reported | 42754 | 23.6 (3.0) | 49.6 (5.9) | 61.6 (7.0) | 20457 (48) | 12124 (28) | 1990 | 15.6 (15.4, 15.9) | ++ | 3391 | 882 | 188 | 399 | 1501 | 201 |
| Japan Public Health Center–based Prospective Study 2 | 149 | Japan | Residential registry | self-reported | 55669 | 23.5 (3.1) | 54.2 (8.8) | 67.1 (8.3) | 26402 (47) | 15312 (28) | 1993 | 12.8 (11.9, 12.9) | ++ | 5346 | 1321 | 337 | 543 | 2418 | 456 |
| Japan Railways | 150 | Japan | Occupational | measured | 54954 | 22.7 (2.8) | 42.8 (7.5) | 53.0 (5.0) | 54954 (100) | 27228 (50) | 1976 | 8.5 (4.5, 10.5) | + | 1367 | 514 | 122 | 224 | 500 | 33 |
| Konan Health and Nutrition Study | 127 | Japan | General popln. | measured | 1188 | 21.9 (3.0) | 51.6 (15.7) | 74.0 (13.0) | 532 (45) | 359 (30) | 1991 | 6.4 (4.4, 8.4) | + | 76 | 23 | 2 | 11 | 25 | 9 |
| Kyowa Study | 147 | Japan | Popln. screening | measured | 4189 | 23.5 (3.2) | 53.9 (8.5) | 67.0 (8.0) | 1816 (43) | 1386 (33) | 1983 | 10.5 (8.5, 11.5) | ++ | 284 | 77 | 17 | 33 | 118 | 23 |
| Life Span Study Cohort | 151 | Japan | General popln. | self-reported | 49245 | 22.0 (3.4) | 52.0 (13.4) | 78.4 (11.4) | 19607 (40) | 19224 (39) | 1969 | 21.9 (11.0, 32.2) | + | 25315 | 9317 | 1924 | 4246 | 7200 | 3313 |
| Miyagi Cohort | 152 | Japan | Residential registry | self-reported | 44845 | 23.6 (3.0) | 51.9 (7.5) | 64.7 (7.6) | 21497 (48) | 14445 (32) | 1990 | 13.6 (13.6, 13.6) | ++ | 3435 | 624 | 156 | 272 | 1279 | 141 |
| Miyama Cohort Study | 153 | Japan | Popln. register | measured | 1031 | 22.1 (2.9) | 60.4 (9.5) | 72.0 (8.0) | 461 (45) | 305 (30) | 1989 | 6.6 (6.1, 6.7) | + | 79 | 18 | 2 | 6 | 34 | 5 |
| Noichi Study | 147 | Japan | Popln. screening | measured | 2226 | 22.9 (3.2) | 53.8 (9.2) | 71.0 (9.0) | 828 (37) | 581 (26) | 1976 | 16.5 (14.5, 18.5) | ++ | 372 | 146 | 20 | 60 | 116 | 27 |
| Ohasama Study | 154 | Japan | General popln. | measured | 3081 | 23.6 (3.2) | 57.2 (12.2) | 71.0 (12.0) | 1213 (39) | 677 (22) | 1990 | 5.5 (4.5, 5.5) | + | 110 | 40 | 12 | 16 | 39 | 9 |
| Ohsaki National Health Insurance Cohort Study | 155 | Japan | Health insurance list | self-reported | 47614 | 23.5 (3.2) | 60.1 (10.2) | 73.9 (9.0) | 22971 (48) | 13621 (29) | 1995 | 12.0 (9.8, 12.0) | + | 6863 | 2107 | 467 | 984 | 2343 | 830 |
| Osaka Study | 156 | Japan | Popln. register | measured | 12601 | 23.0 (2.8) | 52.5 (9.7) | 74.0 (11.0) | 8588 (68) | 4860 (39) | 1992 | 10.2 (6.5, 16.2) | ++ | 721 | 145 | 18 | 38 | 234 | 74 |
| Oyabe study | 157 | Japan | Popln. screening | measured | 5150 | 23.0 (2.9) | 56.9 (11.2) | 75.0 (10.0) | 1608 (31) | 988 (19) | 1988 | 10.4 (10.3, 10.5) | ++ | 470 | 130 | 28 | 68 | 187 | 49 |
| Saitama Cohort Study | 158 | Japan | General popln. | measured | 3598 | 22.4 (2.9) | 54.5 (11.8) | 72.0 (10.0) | 1363 (38) | 1024 (29) | 1987 | 11.0 (9.0, 12.0) | + | 360 | 118 | 24 | 54 | 144 | 38 |
| Seven Countries Japan | 4 | Japan | General popln. | measured | 960 | 22.0 (2.4) | 49.8 (5.7) | 67.0 (8.0) | 960 (100) | 700 (73) | 1958 | 24.5 (16.5, 24.5) | + | 444 | 154 | 34 | 97 | 148 | 36 |
| Shibata Cohort Study | 159 | Japan | General popln. | measured | 2328 | 22.4 (3.0) | 56.7 (11.1) | 76.0 (10.0) | 984 (42) | 770 (33) | 1977 | 20.0 (12.6, 20.0) | ++ | 816 | 337 | 65 | 203 | 206 | 94 |
| Shigaraki Town Study | 160 | Japan | General popln. | measured | 3728 | 22.5 (3.1) | 57.1 (13.9) | 76.0 (8.0) | 1516 (41) | 1063 (29) | 1993 | 4.4 (2.4, 5.4) | + | 113 | 28 | 3 | 12 | 55 | 8 |
| Shirakawa Study | 127 | Japan | General popln. | measured | 4637 | 21.5 (2.8) | 48.0 (12.3) | 67.0 (10.0) | 2121 (46) | 1617 (35) | 1977 | 17.5 (15.5, 18.5) | + | 461 | 163 | 44 | 72 | 164 | 32 |
| Tanno - Soubetsu Study | 161 | Japan | General popln. | measured | 1983 | 23.6 (3.2) | 51.1 (6.9) | 66.0 (8.0) | 930 (47) | 764 (39) | 1977 | 16.4 (16.4, 17.4) | NA | 255 | 73 | 20 | 33 | 90 | 0 |
| Three Prefecture Study Aichi | 162 | Japan | Residential registry | self-reported | 32015 | 22.1 (2.9) | 56.0 (11.1) | 75.6 (11.1) | 15203 (47) | 10099 (32) | 1985 | 15.2 (7.7, 15.5) | + | 5623 | 1947 | 372 | 779 | 1903 | 621 |
| Three Prefecture Study Miyagi | 162 | Japan | Residential registry | self-reported | 29414 | 23.3 (3.4) | 56.7 (11.0) | 75.6 (10.9) | 13260 (45) | 6541 (22) | 1984 | 15.0 (8.0, 15.0) | + | 5789 | 2262 | 442 | 1097 | 1752 | 586 |
| Toyama Study | 163 | Japan | Occupational | measured | 4524 | 23.1 (3.0) | 45.6 (6.6) | 73.0 (11.0) | 2907 (64) | 1751 (39) | 1996 | 12.7 (12.7, 12.8) | + | 97 | 16 | 6 | 3 | 29 | 1 |
| Singapore Chinese Health Study | 164 | Singapore | Household listings | self-reported | 63075 | 23.1 (3.2) | 56.5 (8.0) | 69.8 (8.2) | 27872 (44) | 12277 (19) | 1994 | 12.4 (9.8, 13.8) | + | 10629 | 3689 | 2095 | 1017 | 3888 | 1478 |
| Singapore Thyroid and Heart Study | 165 | Singapore | Census list | measured | 2304 | 23.6 (4.3) | 40.7 (13.4) | 66.0 (11.0) | 1175 (51) | 507 (22) | 1984 | 14.6 (12.7, 15.5) | + | 166 | 58 | 30 | 21 | 35 | 16 |
| Singapore National Health Survey 1992 | 166 | Singapore | General popln. | measured | 3293 | 23.3 (4.1) | 39.2 (12.5) | 57.0 (13.0) | 1589 (48) | 604 (18) | 1992 | 6.2 (6.2, 6.3) | + | 71 | 33 | 22 | 6 | 22 | 2 |
| Korean Cancer Prevention Study | 167 | South Korea | Health insurance list | measured | 1087380 | 23.4 (2.8) | 50.0 (10.4) | 70.8 (11.1) | 672475 (62) | 404324 (37) | 1992 | 18.0 (17.3, 19.0) | + | 167893 | 40382 | 9063 | 21835 | 60919 | 10163 |
| Korean Medical Insurance Corporation Study | 168 | South Korea | Health insurance list | measured | 183368 | 23.0 (2.5) | 44.1 (6.7) | 51.0 (7.0) | 115547 (63) | 61588 (38) | 1992 | 4.0 (4.0, 4.0) | + | 2908 | 490 | 114 | 256 | 1232 | 54 |
| Korean Multi-center Cancer Cohort Study | 169 | South Korea | Popln. screening | measured | 15991 | 23.7 (3.2) | 55.6 (12.2) | 71.1 (10.2) | 6342 (40) | 4110 (26) | 1998 | 6.6 (3.5, 9.3) | ++ | 1293 | 330 | 74 | 183 | 385 | 94 |
| Seoul Male Cancer Cohort | 170 | South Korea | Health insurance list | self-reported | 13951 | 23.4 (2.3) | 49.2 (5.2) | 60.8 (6.4) | 13951 (100) | 6926 (50) | 1992 | 15.1 (14.9, 15.1) | + | 844 | 151 | 54 | 64 | 447 | 26 |

Notes are at the end of eTable 1, on page 10.

## eTable 1 (continued)

| Study | Ref | Country | Population source | Weight & height assessment | Total participants | Mean (SD) BMI | Mean (SD) age at survey | Mean (SD) age at death | No (%) males | No (%) current smokers | Median baseline survey year | Median (IQR) follow-up time | Death ascertainment | Number of deaths | | | | | |
|---|---|---|---|---|---|---|---|---|---|---|---|---|---|---|---|---|---|---|---|
| | | | | | | | | | | | | | | All-cause mortality | CVD | CHD | Stroke | Cancer | Respiratory disease |
| Cardiovascular Disease Risk Factors Two-Township Study | 171 | Taiwan | General popln. | measured | 5005 | 23.8 (3.5) | 48.2 (15.1) | 73.8 (11.9) | 2208 (44) | 1116 (22) | 1991 | 15.7 (14.9, 16.7) | + | 823 | 218 | 49 | 117 | 225 | 82 |
| Community-based Cancer Screening Project | 172 | Taiwan | General popln. | measured | 23716 | 24.0 (3.4) | 47.3 (10.0) | 64.3 (9.4) | 11939 (50) | 6002 (25) | 1991 | 15.9 (15.5, 16.3) | + | 2748 | 558 | 158 | 258 | 1033 | 162 |
| The Kinmen Neurological Disorders Survey | 173 | Taiwan | General popln. | measured | 1262 | 23.4 (3.4) | 62.7 (9.2) | 77.0 (10.0) | 669 (53) | 353 (30) | 1994 | 2.9 (2.8, 2.9) | + | 83 | 34 | 8 | 9 | 30 | 6 |
| Electrical Generating Authority of Thailand Study | 174 | Thailand | Occupational | measured | 3490 | 23.1 (3.1) | 43.0 (5.1) | 53.0 (6.0) | 2697 (77) | 1509 (43) | 1985 | 11.4 (11.4, 11.4) | + | 164 | 51 | 28 | 16 | 43 | 0 |
| **Subtotal (61 studies)** | | | | | **2654305** | **23.0 (3.1)** | **52.4 (9.6)** | **67.8 (9.2)** | **1593389 (60.0)** | **975163 (36.7)** | **1990*** | **13.9 (12.2, 14.8)** | | **370426** | **104070** | **22595** | **52045** | **113326** | **32393** |
| | | | | | | | | | | | | | | | | | | | |
| **South Asia** | | | | | | | | | | | | | | | | | | | |
| Health Effects of Arsenic Longitudinal Study | 175 | Bangladesh | General popln. | measured | 11062 | 19.9 (3.1) | 37.1 (9.9) | 51.6 (11.0) | 4798 (43) | 3189 (29) | 2001 | 6.7 (6.2, 7.1) | ++ | 351 | 93 | 30 | 39 | 59 | 23 |
| Mumbai Cohort Study | 176 | India | Electoral roll | measured | 143111 | 22.5 (4.0) | 50.6 (11.0) | 65.0 (12.0) | 85576 (60) | 21862 (15) | 1993 | 5.7 (5.2, 6.1) | ++ | 11916 | 3796 | 2103 | 762 | 747 | 1061 |
| Trivandrum Oral Cancer Screening Trial | 177 | India | General popln. | measured | 126055 | 22.0 (3.9) | 49.3 (12.2) | 67.3 (12.2) | 48668 (39) | 27597 (22) | 1997 | 8.2 (5.8, 9.2) | + | 9983 | 3829 | 2454 | 1190 | 1216 | 1140 |
| **Subtotal (3 studies)** | | | | | **280228** | **21.5 (3.7)** | **45.7 (11.0)** | **61.3 (11.8)** | **139042 (50)** | **52648 (19)** | **1997*** | **6.9 (5.5, 7.5)** | | **22250** | **7718** | **4587** | **1991** | **2022** | **2224** |
| | | | | | | | | | | | | | | | | | | | |
| **TOTAL (239 studies)** | | | | | **10625411** | **25.2 (3.9)** | **53.1 (8.9)** | **68.5 (8.6)** | **4588709 (43.2)** | **2752921 (26)** | **1989*** | **13.7 (11.4, 14.7)** | | **1601786** | **574740** | **258996** | **142671** | **502004** | **123280** |

Data are for participants with BMI ⩾15 or ⩽60kg/m$^2$, or with a baseline age ⩾20 or ⩽89 years.

Inverse probability sampling weights were used to account for the complex survey study design of the National Health and Nutrition Examination Survey I and III and the National Health Interview Survey.

Ref = reference number in the list of references in eAppendix 5.

NA: Not available.

Death ascertainment method: +, Death certificate only; ++, Death certificate supplemented by medical record.

** EPIC participants were recruited from the general population of their respective countries, with the following exceptions: the French cohort was occupational; the Italian and Spanish studies included members of blood donor associations and the general population; the Utrecht (the Netherlands) and Florence (Italy) studies were from population screening; the Oxford UK study included many vegetarians, vegans, and low meat eaters; and the studies in France, Norway, Naples (Italy) and Utrecht (the Netherlands) included only women.

†Analysis omitted data on 2961 participants from two studies that each recorded fewer than 10 deaths, so a total of 12 deaths were omitted from the Capital Iron & Steel Company Hospital Cohort and the Risk Factors and Life Expectancy Pooling Project (GREPCO).

* For subtotals and for the total (all studies), the table gives the median of the study-specific median baseline survey year; for all 239 studies this is 1989 (IQR 1980-1993)

## eTable 2: Chronic diseases recorded in each participating study at baseline examination

| Study | Ref | Country | Heart disease | Stroke | Other vascular disease | Cancer | Respiratory diseases |
|---|---|---|---|---|---|---|---|
| **Europe** | | | | | | | |
| Vorarlberg Health Monitoring and Promotion Programme | 1 | Austria | | | | | |
| Belgian Inter-university Research on Nutrition and Health | 2 | Belgium | ✔ | | | | |
| Flemish Study on Environment Genes and Health (FLEMENGHO) | 3 | Belgium | | | | | |
| Seven Countries Croatia | 4 | Croatia | ✔ | | | | |
| Copenhagen City Heart Study | 5 | Denmark | ✔ | ✔ | | | |
| Research Centre for Prevention and Health (Glostrup Population Studies) | 6 | Denmark | ✔ | | | | |
| Finnish Mobile Clinic Survey | 7 | Finland | ✔ | | | | |
| Finrisk Cohort 1977 | 8 | Finland | ✔ | ✔ | | | |
| Finrisk Cohort 1982 | 8 | Finland | ✔ | ✔ | | | |
| Finrisk Cohort 1987 | 8 | Finland | ✔ | ✔ | | | |
| Finrisk Cohort 1992 | 8 | Finland | ✔ | ✔ | | ✔ | |
| Finrisk Cohort 1997 | 8 | Finland | ✔ | ✔ | | ✔ | |
| Helsinki Aging Study | 9 | Finland | ✔ | | | | |
| Helsinki Businessmen Study | 10 | Finland | ✔ | | | | |
| Kuopio Ischaemic Heart Disease Study | 11 | Finland | ✔ | ✔ | ✔ | ✔ | ✔ |
| North Karelia Project | 12 | Finland | ✔ | ✔ | | ✔ | |
| Seven Countries Finland | 4 | Finland | ✔ | ✔ | | | |
| Centre d'Investigations Preventives et Cliniques (IPC) Paris | 13 | France | ✔ | | | | |
| Data from an Epidemiological Study on the Insulin Resistance Syndrome | 14 | France | ✔ | ✔ | ✔ | | |
| Paris Prospective Study I | 15 | France | ✔ | ✔ | | | |
| Prospective Epidemiological Study of Myocardial Infarction | 16 | France / NI | ✔ | ✔ | ✔ | ✔ | ✔ |
| Epidemiologische Studie zu Chancen der Verhütung | 17 | Germany | ✔ | ✔ | | ✔ | |
| Göttingen Risk Incidence and Prevalence Study | 18 | Germany | ✔ | ✔ | ✔ | | |
| MONICA/KORA Augsburg Survey 1 | 19 | Germany | ✔ | ✔ | | | |
| MONICA/KORA Augsburg Survey 2 | 19 | Germany | ✔ | ✔ | | | |
| MONICA/KORA Augsburg Survey 3 | 19 | Germany | ✔ | ✔ | | | |
| Prospective Cardiovascular Münster Study | 20 | Germany | ✔ | ✔ | | | |
| Study of Health in Pomerania | 21 | Germany | ✔ | ✔ | ✔ | ✔ | |
| Württemberg Construction Workers Cohort | 22 | Germany | ✔ | ✔ | | | |
| ATTICA Study | 23 | Greece | | | | | |
| Seven Countries Greece | 4 | Greece | ✔ | ✔ | | | |
| Reykjavik Study | 24 | Iceland | ✔ | ✔ | ✔ | ✔ | |
| Bezafibrate Infarction Prevention Registry Study | 25 | Israel | ✔ | ✔ | ✔ | | |
| Israeli Ischaemic Heart Disease Study | 26 | Israel | ✔ | | | | |
| Glucose Intolerance Obesity and Hypertension Study | 27 | Israel | ✔ | ✔ | | | |
| Bruneck Study | 28 | Italy | ✔ | ✔ | ✔ | | |
| Cardiovascular Study in the Elderly | 29 | Italy | ✔ | ✔ | ✔ | | ✔ |
| Occupational Groups (OG), Rome | 30 | Italy | ✔ | ✔ | | | |
| Progetto CUORE (ATENA) | 31 | Italy | ✔ | ✔ | | | |
| Progetto CUORE (EMOFRI) | 31 | Italy | ✔ | ✔ | | | |
| Progetto CUORE (MATISS83) | 31 | Italy | ✔ | ✔ | | ✔ | |
| Progetto CUORE (MATISS87) | 31 | Italy | ✔ | ✔ | | ✔ | |
| Progetto CUORE (MATISS93) | 31 | Italy | ✔ | ✔ | | ✔ | |
| Progetto CUORE (MONFRI86) | 31 | Italy | ✔ | ✔ | | | |
| Progetto CUORE (MONFRI89) | 31 | Italy | ✔ | ✔ | | | |
| Progetto CUORE (MONFRI94) | 31 | Italy | ✔ | ✔ | | | |
| Risk Factors and Life Expectancy Pooling Project (ATS_SAR) | 32 | Italy | ✔ | ✔ | | | |
| Risk Factors and Life Expectancy Pooling Project (DISCO) | 32 | Italy | ✔ | ✔ | | | |
| Risk Factors and Life Expectancy Pooling Project (GREPCO) | 32 | Italy | ✔ | ✔ | | | |
| Risk Factors and Life Expectancy Pooling Project (GUBBIO) | 32 | Italy | | | | | |
| Risk Factors and Life Expectancy Pooling Project (MICOL) | 32 | Italy | ✔ | ✔ | | | |
| Risk Factors and Life Expectancy Pooling Project (MONICA) | 32 | Italy | | | | | |
| Risk Factors and Life Expectancy Pooling Project (NFR) | 32 | Italy | ✔ | ✔ | | | |
| Risk Factors and Life Expectancy Pooling Project (OB43) | 32 | Italy | ✔ | ✔ | | | |
| Risk Factors and Life Expectancy Pooling Project (RF2) | 32 | Italy | ✔ | ✔ | | | |
| Seven Countries Italy | 4 | Italy | ✔ | ✔ | | | |
| Vicenza Thrombophilia and Athrosclerosis Project | 33 | Italy | ✔ | ✔ | ✔ | | |
| Consultation Bureau Project | 34 | Netherlands | ✔ | ✔ | | | |
| Hoorn Study | 35 | Netherlands | ✔ | ✔ | ✔ | | |
| Longitudinal Aging Study Amsterdam | 36 | Netherlands | ✔ | ✔ | ✔ | | |

Ref = reference number in the list of references in eAppendix 5.

## eTable 2 (continued)

| Study | Ref | Country | Heart disease | Stroke | Other vascular disease | Cancer | Respiratory diseases |
|---|---|---|---|---|---|---|---|
| Monitoring of CVD Risk Factors Project | 37 | Netherlands | ✔ | ✔ | ✔ | | |
| Prevention of Renal and Vascular End Stage Disease Study | 38 | Netherlands | ✔ | ✔ | ✔ | | |
| Seven Countries Netherlands | 4 | Netherlands | ✔ | ✔ | | | |
| The Rotterdam Study | 39 | Netherlands | ✔ | ✔ | ✔ | | |
| The Rotterdam Study II | 39 | Netherlands | ✔ | ✔ | ✔ | | |
| The Rotterdam Study III | 39 | Netherlands | ✔ | ✔ | ✔ | | |
| Cohort of Norway (FINNMARK) | 40 | Norway | ✔ | ✔ | | | |
| Cohort of Norway (HUBRO) | 40 | Norway | ✔ | ✔ | | | |
| Cohort of Norway (OPPHED) | 40 | Norway | ✔ | ✔ | | | |
| Cohort of Norway (OSLO2) | 40 | Norway | ✔ | ✔ | | | |
| Cohort of Norway (TROMS) | 40 | Norway | ✔ | ✔ | | | |
| Norwegian Counties Study (NCS1) | 41 | Norway | ✔ | ✔ | | | |
| Norwegian Counties Study (NCS2) | 41 | Norway | ✔ | ✔ | | | |
| Norwegian Counties Study (NCS3) | 41 | Norway | ✔ | ✔ | | | |
| Oslo Study | 42 | Norway | ✔ | ✔ | | | |
| Tromsø Study | 43 | Norway | ✔ | ✔ | ✔ | | |
| Edinburgh Artery Study | 44 | Scotland | ✔ | ✔ | ✔ | | |
| Prospective Study of Pravastatin in the Elderly at Risk | 45 | Scotland/Ireland/Netherland | ✔ | ✔ | ✔ | | |
| Seven Countries Serbia | 4 | Serbia | ✔ | ✔ | | | |
| Diet and Risk of Cardiovascular Disease in Spain | 46 | Spain | ✔ | ✔ | | | |
| Zaragoza study | 47 | Spain | | | | | |
| Apolipoprotein Related Mortality Risk Study | 48 | Sweden | ✔ | ✔ | ✔ | ✔ | |
| Cohort of Swedish Men | 49 | Sweden | ✔ | | | ✔ | |
| Göteborg 1913 Study | 50 | Sweden | ✔ | ✔ | | | |
| Göteborg 1933 Study | 50 | Sweden | ✔ | ✔ | | | |
| Göteborg 1943 Study | 50 | Sweden | ✔ | ✔ | | | |
| MONICA Göteborg Study | 19 | Sweden | ✔ | ✔ | | | |
| Malmö Prevention Project | 51 | Sweden | ✔ | ✔ | | ✔ | |
| Prospective Study of Women in Gothenburg | 52 | Sweden | ✔ | ✔ | | ✔ | |
| Swedish Mammography Cohort | 53 | Sweden | ✔ | | | ✔ | |
| Uppsala Longitudinal Study of Adult Men | 54 | Sweden | ✔ | ✔ | ✔ | ✔ | |
| Women's Lifestyle and Health Study | 55 | Sweden | ✔ | | | ✔ | |
| Turkish Adult Risk Factor Study | 56 | Turkey | ✔ | ✔ | ✔ | | |
| British Regional Heart Study | 57 | UK | ✔ | ✔ | | | |
| British Union Provident Association Study | 58 | UK | ✔ | ✔ | ✔ | | |
| British Women's Heart and Health Study | 59 | UK | ✔ | ✔ | ✔ | | |
| Caerphilly Prospective Study | 60 | UK | ✔ | ✔ | | | |
| Hertfordshire Cohort Study | 61 | UK | ✔ | ✔ | | | |
| Lower Extremity Arterial Disease Event Reduction Trial | 62 | UK | ✔ | ✔ | | | |
| Midspan Family Study | 63 | UK | ✔ | ✔ | | | |
| The Million Women Study | 64 | UK | ✔ | ✔ | ✔ | ✔ | ✔ |
| MRC Study of Older People | 65 | UK | ✔ | ✔ | | ✔ | |
| Midspan Collaborative Study | 66 | UK | ✔ | | | | |
| Northwick Park Heart Study I | 66 | UK | ✔ | ✔ | | | |
| Northwick Park Heart Study II | 67 | UK | ✔ | | | ✔ | |
| Renfew and Paisley Study | 68 | UK | ✔ | ✔ | | | |
| Scottish Heart Health Study | 60 | UK | ✔ | ✔ | ✔ | | |
| Speedwell Study | 69 | UK | ✔ | ✔ | | | |
| Thrombosis Prevention Trial | 70 | UK | | | | | |
| UK Biobank | 71 | UK | ✔ | ✔ | ✔ | ✔ | ✔ |
| United Kingdom Heart Disease Prevention Project | 72 | UK | ✔ | ✔ | | | |
| West of Scotland Coronary Prevention Study | 73 | UK | ✔ | | | | |
| Whitehall I Study | 74 | UK | ✔ | ✔ | | | |
| Whitehall II Study | 75 | UK | ✔ | ✔ | | | |
| European Prospective Investigation into Cancer and Nutrition | 76 | Europe | ✔ | ✔ | ✔ | | |
| **North America** | | | | | | | |
| Nova Scotia Health Survey | 77 | Canada | ✔ | ✔ | ✔ | ✔ | |
| Quebec Cardiovascular Study | 78 | Canada | ✔ | ✔ | ✔ | ✔ | |
| Agricultural Health Study | 79 | USA | ✔ | | | ✔ | |
| Air Force/Texas Coronary Atherosclerosis Prevention Study | 80 | USA | | ✔ | | ✔ | |
| NIH-AARP Diet and Health | 81 | USA | ✔ | | | ✔ | |

Ref = reference number in the list of references in eAppendix 5.

## eTable 2 (continued)

| Study | Ref | Country | Heart disease | Stroke | Other vascular disease | Cancer | Respiratory diseases |
|---|---|---|---|---|---|---|---|
| Atherosclerosis Risk in Communities Study | 82 | USA | ✔ | ✔ | ✔ | ✔ | ✔ |
| Breast Cancer Detection Demonstration Project | 83 | USA | ✔ | | | ✔ | |
| CLUE II | 84 | USA | | | | ✔ | |
| California Teachers Study | 85 | USA | ✔ | | | ✔ | |
| Cancer Prevention Study I | 86 | USA | ✔ | ✔ | | ✔ | ✔ |
| Cancer Prevention Study II | 86 | USA | ✔ | ✔ | | ✔ | ✔ |
| Cardiovascular Health Study (CHS1) | 87 | USA | ✔ | ✔ | ✔ | | |
| Cardiovascular Health Study (CHS2) | 87 | USA | ✔ | ✔ | ✔ | | |
| Charleston Heart Study | 88 | USA | ✔ | ✔ | | ✔ | |
| Chicago Heart Association Detection Project In Industry | 89 | USA | | | | | |
| Established Populations for the Epidemiologic Studies of the Elderly - East Boston | 90 | USA | ✔ | ✔ | ✔ | ✔ | |
| Established Populations for the Epidemiologic Studies of the Elderly - Iowa | 90 | USA | ✔ | ✔ | ✔ | ✔ | |
| Established Populations for the Epidemiologic Studies of the Elderly - New Haven | 90 | USA | ✔ | ✔ | ✔ | ✔ | |
| Established Populations for the Epidemiologic Studies of the Elderly - North Carolina | 90 | USA | ✔ | ✔ | ✔ | ✔ | |
| Framingham Offspring Study | 91 | USA | | | | | |
| Health Professionals Follow-up Study | 92 | USA | ✔ | ✔ | | | |
| Honolulu Heart Program | 93 | USA | ✔ | ✔ | | | |
| Iowa Women's Health Study | 94 | USA | ✔ | | | ✔ | |
| Lipid Research Clinics Follow-up Study | 95 | USA | ✔ | | | | |
| Minnesota Heart Health Project | 96 | USA | ✔ | ✔ | | | |
| Minnesota Heart Survey | 97 | USA | ✔ | ✔ | | | |
| Multi-Ethnic Study of Atherosclerosis | 98 | USA | ✔ | ✔ | ✔ | | |
| Multiple Risk Factor Intervention Trial | 99 | USA | ✔ | ✔ | | ✔ | |
| NHLBI Framingham Heart Study | 100 | USA | ✔ | ✔ | | | |
| NYU Women's Health Study | 101 | USA | ✔ | | | ✔ | |
| National Health and Nutrition Examination Survey I | 102 | USA | ✔ | ✔ | | | |
| National Health and Nutrition Examination Survey III | 103 | USA | ✔ | ✔ | ✔ | ✔ | |
| National Health Interview Survey | 104 | USA | ✔ | ✔ | | ✔ | |
| Nurses' Health Study I | 105 | USA | ✔ | | | | |
| Nurses' Health Study II | 106 | USA | ✔ | ✔ | ✔ | ✔ | ✔ |
| Prostate, Lung, Colorectal and Ovarian (PLCO) Cancer Screening Trial | 107 | USA | ✔ | | | ✔ | |
| Physicians' Health Study I & II | 108 | USA | ✔ | | | ✔ | |
| Puerto Rico Heart Health Program | 109 | USA | ✔ | ✔ | ✔ | | |
| Rancho Bernardo Study | 110 | USA | ✔ | ✔ | | | |
| Seven Countries US | 4 | USA | ✔ | ✔ | | | |
| Seventh-day Adventist Cohort Study | 111 | USA | ✔ | | | ✔ | |
| Strong Heart Study | 112 | USA | ✔ | ✔ | | | |
| Tecumseh Study | 113 | USA | ✔ | | | | |
| U.S. Radiologic Technologists (URST) Cohort | 114 | USA | ✔ | | | ✔ | |
| Vitamins and Lifestyle (VITAL) Study | 115 | USA | ✔ | | | ✔ | |
| Women's Health Initiative (Oberservational Study) | 116 | USA | ✔ | ✔ | ✔ | | |
| Women's Health Study | 117 | USA | ✔ | | | ✔ | |
| Antihypertensive and Lipid-Lowering Treatment to Prevent Heart Attack Trial | 118 | USA/Canada/Puerto Rico/US Virgin Islands | ✔ | ✔ | ✔ | | |
| **Australia/New Zealand** | | | | | | | |
| 45 and Up Study | 119 | Australia | ✔ | ✔ | | ✔ | |
| Abdominal Aortic Aneurysm Screening Program | 120 | Australia | ✔ | ✔ | | | |
| Australian Diabetes, Obesity and Lifestyle Study | 121 | Australia | ✔ | ✔ | ✔ | | |
| Australian Longitudinal Study of Ageing | 122 | Australia | ✔ | ✔ | | | |
| Busselton Health Study | 123 | Australia | ✔ | ✔ | | | |
| Dubbo Study of the Elderly | 124 | Australia | ✔ | ✔ | ✔ | ✔ | |
| Health in Men Study | 125 | Australia | ✔ | ✔ | ✔ | | |
| National Heart Foundation Risk Factor Prevalence Survey 1989 | 126 | Australia | ✔ | ✔ | | | |
| Newcastle Study | 127 | Australia | ✔ | ✔ | | | |
| Perth Risk Factors Survey | 128 | Australia | ✔ | ✔ | | | |
| Melbourne Collaborative Cohort Study | 129 | Australia/New Zealand/Europe | | | | ✔ | |
| Fletcher Challenge Heart and Health Study | 64 | New Zealand | ✔ | ✔ | | | |
| **East Asia** | | | | | | | |
| Anzhen 02 Cohort Study | 127 | China | | | | | |
| Beijing Anzhen Cohort Study | 127 | China | ✔ | ✔ | | | |
| Beijing Aging Study | 130 | China | ✔ | ✔ | | | |
| Capital Iron & Steel Company Study | 131 | China | ✔ | ✔ | | | |
| Capital Iron & Steel Company Hospital Cohort | 127 | China | ✔ | ✔ | | | |
| China Hypertension Survey Epidemiology Follow-up Study | 132 | China | ✔ | ✔ | ✔ | | |
| China Prospective Study | 133 | China | ✔ | ✔ | | ✔ | ✔ |
| East Beijing Cohort Study | 127 | China | ✔ | ✔ | | | |

Ref = reference number in the list of references in eAppendix 5.

## eTable 2 (continued)

| Study | Ref | Country | Heart disease | Stroke | Other vascular disease | Cancer | Respiratory diseases |
|---|---|---|---|---|---|---|---|
| Fangshan Cohort Study | 134 | China | ✔ | ✔ | | | |
| Huashan Study | 127 | China | ✔ | ✔ | | | |
| Nutrition Intervention Trials - Linxian | 135 | China | ✔ | ✔ | ✔ | | |
| Seven Cities Cohorts Study | 127 | China | ✔ | ✔ | | | |
| Shanghai Cohort Study | 136 | China | | | | | |
| Shanghai Men's Health Study | 137 | China | ✔ | | ✔ | | ✔ |
| Shanghai Women's Health Study | 138 | China | ✔ | ✔ | ✔ | | ✔ |
| Six Chinese Cohorts Study | 127 | China | | | | | |
| Tianjin Study | 139 | China | ✔ | ✔ | | | |
| Yunnan Tin Miner Cohort Study | 140 | China | | | | | |
| Guangzhou Occupational Cohort study | 141 | Hong Kong | | | | | |
| Hong Kong Study | 142 | Hong Kong | ✔ | ✔ | | | |
| Aito Town Study | 127 | Japan | ✔ | ✔ | | | |
| Akabane Study | 127 | Japan | ✔ | ✔ | | | |
| Civil Service Workers Study | 143 | Japan | ✔ | ✔ | | | |
| Funagata Study | 144 | Japan | | | | | |
| Hisayama Study | 145 | Japan | | ✔ | | | |
| Ibaraki Prefectural Health Study | 146 | Japan | ✔ | ✔ | ✔ | | |
| Ikawa, Kyowa, Noichi Study | 147 | Japan | ✔ | ✔ | | | |
| Japan Collaborative Cohort Study | 148 | Japan | ✔ | ✔ | ✔ | ✔ | |
| Japan Public Health Center–based Prospective Study 1 | 149 | Japan | ✔ | ✔ | ✔ | ✔ | |
| Japan Public Health Center–based Prospective Study 2 | 149 | Japan | ✔ | ✔ | ✔ | ✔ | |
| Japan Railways | 150 | Japan | | | | | |
| Konan Health and Nutrition Study | 127 | Japan | ✔ | ✔ | | | |
| Kyowa Study | 147 | Japan | ✔ | ✔ | | | |
| Life Span Study Cohort | 151 | Japan | | | | | |
| Miyagi Cohort | 152 | Japan | ✔ | ✔ | ✔ | ✔ | |
| Miyama Cohort Study | 153 | Japan | ✔ | ✔ | | | |
| Noichi Study | 147 | Japan | ✔ | ✔ | | | |
| Ohasama Study | 154 | Japan | ✔ | ✔ | | | |
| Ohsaki National Health Insurance Cohort Study | 155 | Japan | ✔ | ✔ | ✔ | ✔ | |
| Osaka Study | 156 | Japan | ✔ | ✔ | | | |
| Oyabe study | 157 | Japan | | ✔ | | | |
| Saitama Cohort Study | 158 | Japan | ✔ | ✔ | | | |
| Seven Countries Japan | 4 | Japan | ✔ | ✔ | | | |
| Shibata Cohort Study | 159 | Japan | ✔ | ✔ | | | |
| Shigaraki Town Study | 160 | Japan | ✔ | ✔ | | | |
| Shirakawa Study | 127 | Japan | ✔ | ✔ | | | |
| Tanno - Soubetsu Study | 161 | Japan | | | | | |
| Three Prefecture Study Aichi | 162 | Japan | ✔ | | ✔ | ✔ | ✔ |
| Three Prefecture Study Miyagi | 162 | Japan | ✔ | ✔ | ✔ | ✔ | |
| Toyama Study | 163 | Japan | | | | | |
| Singapore Chinese Health Study | 164 | Singapore | ✔ | ✔ | ✔ | ✔ | |
| Singapore Thyroid and Heart Study | 165 | Singapore | ✔ | ✔ | | | |
| Singapore National Health Survey 1992 | 166 | Singapore | ✔ | ✔ | | | |
| Korean Cancer Prevention Study | 167 | South Korea | ✔ | ✔ | | ✔ | ✔ |
| Korean Medical Insurance Corporation Study | 168 | South Korea | | | | | |
| Korean Multi-center Cancer Cohort Study | 169 | South Korea | | ✔ | | ✔ | ✔ |
| Seoul Male Cancer Cohort | 170 | South Korea | ✔ | ✔ | ✔ | | |
| Cardiovascular Disease Risk Factors Two-Township Study | 171 | Taiwan | ✔ | | ✔ | ✔ | |
| Community-based Cancer Screening Project | 172 | Taiwan | ✔ | ✔ | ✔ | ✔ | |
| The Kinmen Neurological Disorders Survey | 173 | Taiwan | ✔ | ✔ | | | |
| Electrical Generating Authority of Thailand Study | 174 | Thailand | | | | | |
| **South Asia** | | | | | | | |
| Health Effects of Arsenic Longitudinal Study | 175 | Bangladesh | ✔ | | ✔ | ✔ | |
| Mumbai Cohort Study | 176 | India | | | | | |
| Trivandrum Oral Cancer Screening Trial | 177 | India | ✔ | | ✔ | ✔ | ✔ |

Ref = reference number in the list of references in eAppendix 5.

**eTable 3: Mortality outcomes and their International Classification of Diseases codes**

| | ICD version | | |
|---|---|---|---|
| | **ICD-8** | **ICD-9** | **ICD-10** |
| Cardiovascular disease | 390-458, 795 | 390-459, 798 | I00-I99, R96 |
| Coronary heart disease | 410-414 | 410-414 | I20-I25 |
| Stroke | 430-438 | 430-438 | I60-I69 |
| Neoplastic disease | 140-239 | 140-239 | C00-C97, D00-D48 |
| Respiratory disease | 460-519 | 460-519 | J00-J99 |

## eTable 4: Six WHO BMI groups vs all-cause mortality, by successively stricter inclusion criteria, in all 237 studies

| Exclude baseline chronic disease? | Adjust for smoking? | Exclude the first 5years follow-up? | Restrict analyses to never-smokers? | No of studies | No of partici-pants | No of deaths | Underweight (BMI 15 to <18.5) | | Normal weight (18.5 to <25) | | Overweight (25 to <30) | | Obesity Grade I (30 to <35) | | Obesity Grade II (35 to <40) | | Obesity Grade III (40 to <60) | |
|---|---|---|---|---|---|---|---|---|---|---|---|---|---|---|---|---|---|---|
| | | | | | | | No of deaths | HR (95% CI) | No of deaths | HR (95% CI) | No of deaths | HR (95% CI) | No of deaths | HR (95% CI) | No of deaths | HR (95% CI) | No of deaths | HR (95% CI) |
| **All Studies** | | | | | | | | | | | | | | | | | | |
| | | | | 237 | 10,622,450 | 1,601,774 | 68,455 | 1.82 (1.74, 1.91) | 810,838 | 1.00 (0.98, 1.02) | 526,098 | 0.95 (0.94, 0.97) | 144,871 | 1.17 (1.16, 1.18) | 36,113 | 1.49 (1.47, 1.51) | 15,399 | 1.95 (1.90, 2.01) |
| √ | | | | 236 | 9,104,247 | 1,210,250 | 52,789 | 1.81 (1.72, 1.91) | 631,488 | 1.00 (0.98, 1.02) | 388,781 | 0.95 (0.95, 0.96) | 102,315 | 1.18 (1.16, 1.20) | 24,556 | 1.52 (1.48, 1.55) | 10,321 | 2.05 (1.98, 2.13) |
| √ | √ | | | 234 | 8,801,617 | 1,185,728 | 51,170 | 1.70 (1.61, 1.80) | 618,881 | 1.00 (0.98, 1.02) | 381,617 | 0.99 (0.98, 1.00) | 100,113 | 1.25 (1.23, 1.27) | 23,945 | 1.63 (1.59, 1.66) | 10,002 | 2.24 (2.15, 2.33) |
| √ | √ | √ | | 213 | 7,805,434 | 949,010 | 33,817 | 1.60 (1.51, 1.70) | 496,310 | 1.00 (0.98, 1.02) | 312,450 | 1.03 (1.01, 1.04) | 80,037 | 1.31 (1.29, 1.33) | 18,737 | 1.70 (1.67, 1.74) | 7,659 | 2.36 (2.27, 2.45) |
| √ | | | √ | 215 | 4,425,240 | 481,928 | 20,091 | 1.53 (1.45, 1.60) | 241,359 | 1.00 (0.98, 1.02) | 158,243 | 1.07 (1.07, 1.08) | 45,802 | 1.38 (1.36, 1.41) | 11,504 | 1.81 (1.76, 1.87) | 4,929 | 2.54 (2.41, 2.67) |
| √ | | √ | √ | 189 | 3,951,455 | 385,879 | 12,726 | 1.47 (1.39, 1.55) | 192,523 | 1.00 (0.98, 1.02) | 130,293 | 1.11 (1.10, 1.11) | 37,318 | 1.44 (1.41, 1.47) | 9,179 | 1.92 (1.86, 1.98) | 3,840 | 2.71 (2.55, 2.86) |
| **Europe** | | | | | | | | | | | | | | | | | | |
| | | | | 114 | 3,389,305 | 305,601 | 5,823 | 2.14 (1.98, 2.31) | 127,353 | 1.00 (0.98, 1.02) | 117,996 | 0.95 (0.94, 0.95) | 39,533 | 1.15 (1.13, 1.18) | 10,674 | 1.49 (1.45, 1.54) | 4,222 | 2.06 (1.98, 2.14) |
| √ | | | | 113 | 2,924,772 | 241,415 | 4,464 | 2.14 (1.98, 2.31) | 103,986 | 1.00 (0.98, 1.02) | 92,812 | 0.95 (0.95, 0.95) | 29,564 | 1.16 (1.13, 1.18) | 7,664 | 1.49 (1.45, 1.54) | 2,925 | 2.11 (2.02, 2.21) |
| √ | √ | | | 112 | 2,773,056 | 231,023 | 4,279 | 1.92 (1.79, 2.07) | 99,750 | 1.00 (0.98, 1.02) | 88,891 | 1.00 (0.99, 1.00) | 28,132 | 1.24 (1.21, 1.26) | 7,262 | 1.64 (1.58, 1.69) | 2,709 | 2.35 (2.24, 2.47) |
| √ | √ | √ | | 105 | 2,507,123 | 184,323 | 3,120 | 1.75 (1.62, 1.90) | 80,225 | 1.00 (0.98, 1.02) | 71,068 | 1.03 (1.03, 1.03) | 22,097 | 1.29 (1.26, 1.32) | 5,652 | 1.73 (1.66, 1.79) | 2,161 | 2.56 (2.42, 2.70) |
| √ | | | √ | 98 | 1,245,060 | 70,865 | 910 | 1.72 (1.59, 1.86) | 27,912 | 1.00 (0.97, 1.03) | 27,216 | 1.09 (1.09, 1.10) | 10,488 | 1.43 (1.38, 1.49) | 3,125 | 1.90 (1.80, 2.01) | 1,214 | 2.81 (2.63, 3.01) |
| √ | | √ | √ | 89 | 1,135,600 | 56,477 | 675 | 1.69 (1.56, 1.85) | 22,235 | 1.00 (0.96, 1.04) | 21,785 | 1.12 (1.11, 1.13) | 8,386 | 1.51 (1.45, 1.56) | 2,424 | 2.01 (1.87, 2.17) | 972 | 3.00 (2.80, 3.22) |
| **North America** | | | | | | | | | | | | | | | | | | |
| | | | | 48 | 3,932,741 | 867,889 | 20,982 | 1.80 (1.69, 1.92) | 397,766 | 1.00 (0.97, 1.03) | 322,182 | 0.96 (0.95, 0.97) | 93,096 | 1.16 (1.14, 1.19) | 23,410 | 1.46 (1.42, 1.51) | 10,453 | 1.95 (1.85, 2.07) |
| √ | | | | 48 | 3,114,974 | 594,278 | 12,348 | 1.75 (1.63, 1.88) | 272,770 | 1.00 (0.97, 1.03) | 223,187 | 0.97 (0.96, 0.98) | 63,550 | 1.18 (1.15, 1.21) | 15,521 | 1.49 (1.43, 1.55) | 6,902 | 2.05 (1.92, 2.19) |
| √ | √ | | | 47 | 3,083,899 | 590,668 | 12,281 | 1.59 (1.49, 1.70) | 271,427 | 1.00 (0.98, 1.02) | 221,752 | 1.01 (1.01, 1.02) | 63,028 | 1.26 (1.23, 1.29) | 15,354 | 1.60 (1.53, 1.67) | 6,826 | 2.22 (2.07, 2.38) |
| √ | √ | √ | | 45 | 2,789,678 | 496,720 | 9,456 | 1.50 (1.42, 1.59) | 230,905 | 1.00 (0.98, 1.02) | 187,109 | 1.04 (1.03, 1.05) | 51,771 | 1.32 (1.28, 1.35) | 12,254 | 1.72 (1.65, 1.80) | 5,225 | 2.39 (2.23, 2.55) |
| √ | | | √ | 43 | 1,563,123 | 258,393 | 5,029 | 1.46 (1.37, 1.57) | 116,307 | 1.00 (0.97, 1.03) | 95,761 | 1.08 (1.06, 1.09) | 30,185 | 1.36 (1.31, 1.40) | 7,664 | 1.83 (1.73, 1.94) | 3,447 | 2.46 (2.27, 2.66) |
| √ | | √ | √ | 40 | 1,415,087 | 219,922 | 3,846 | 1.44 (1.33, 1.55) | 99,792 | 1.00 (0.97, 1.03) | 81,935 | 1.10 (1.08, 1.12) | 25,348 | 1.40 (1.35, 1.44) | 6,299 | 1.96 (1.84, 2.08) | 2,702 | 2.64 (2.41, 2.88) |
| **Australia / NZ** | | | | | | | | | | | | | | | | | | |
| | | | | 12 | 368,036 | 35,615 | 821 | 2.05 (1.80, 2.33) | 13,535 | 1.00 (0.97, 1.03) | 14,301 | 0.90 (0.87, 0.92) | 5,139 | 1.04 (0.99, 1.10) | 1,353 | 1.43 (1.35, 1.53) | 466 | 2.02 (1.84, 2.23) |
| √ | | | | 12 | 289,343 | 20,814 | 441 | 1.99 (1.76, 2.25) | 7,919 | 1.00 (0.97, 1.04) | 8,410 | 0.90 (0.86, 0.93) | 3,004 | 1.04 (0.97, 1.11) | 782 | 1.45 (1.33, 1.57) | 258 | 2.00 (1.76, 2.29) |
| √ | √ | | | 12 | 280,326 | 20,193 | 416 | 1.81 (1.58, 2.07) | 7,623 | 1.00 (0.97, 1.04) | 8,182 | 0.94 (0.88, 0.99) | 2,951 | 1.09 (1.01, 1.17) | 767 | 1.52 (1.39, 1.68) | 254 | 2.10 (1.85, 2.39) |
| √ | √ | √ | | 11 | 260,710 | 11,655 | 163 | 1.55 (1.33, 1.82) | 4,218 | 1.00 (0.94, 1.07) | 4,930 | 0.98 (0.94, 1.02) | 1,774 | 1.19 (1.14, 1.25) | 453 | 1.73 (1.51, 1.97) | 117 | 1.85 (1.46, 2.33) |
| √ | | | √ | 12 | 157,904 | 8,169 | 159 | 1.51 (1.29, 1.78) | 3,148 | 1.00 (0.97, 1.03) | 3,231 | 0.99 (0.94, 1.05) | 1,175 | 1.21 (1.12, 1.30) | 339 | 1.66 (1.46, 1.89) | 117 | 2.12 (1.64, 2.76) |
| √ | | √ | √ | 11 | 149,602 | 4,656 | 63 | 1.41 (1.10, 1.82) | 1,692 | 1.00 (0.96, 1.04) | 1,924 | 1.01 (0.93, 1.08) | 717 | 1.28 (1.18, 1.40) | 203 | 1.76 (1.53, 2.03) | 57 | 2.11 (1.62, 2.74) |
| **East Asia** | | | | | | | | | | | | | | | | | | |
| | | | | 60 | 2,652,140 | 370,419 | 34,513 | 1.49 (1.35, 1.64) | 259,718 | 1.00 (0.99, 1.01) | 68,738 | 0.99 (0.95, 1.02) | 6,613 | 1.32 (1.25, 1.39) | 604 | 1.66 (1.49, 1.84) | 233 | 1.54 (1.29, 1.84) |
| √ | | | | 60 | 2,500,583 | 332,658 | 29,713 | 1.48 (1.33, 1.65) | 234,917 | 1.00 (0.99, 1.01) | 61,574 | 0.98 (0.94, 1.02) | 5,723 | 1.30 (1.23, 1.38) | 519 | 1.74 (1.53, 1.98) | 212 | 1.61 (1.33, 1.93) |
| √ | √ | | | 60 | 2,389,761 | 322,759 | 28,371 | 1.48 (1.30, 1.68) | 228,185 | 1.00 (0.98, 1.02) | 59,994 | 1.00 (0.97, 1.04) | 5,528 | 1.31 (1.26, 1.37) | 492 | 1.73 (1.51, 1.97) | 189 | 1.67 (1.35, 2.07) |
| √ | √ | √ | | 49 | 2,029,374 | 250,310 | 19,434 | 1.37 (1.18, 1.60) | 177,579 | 1.00 (0.98, 1.02) | 48,534 | 1.04 (1.00, 1.08) | 4,259 | 1.36 (1.30, 1.42) | 354 | 1.60 (1.41, 1.82) | 150 | 1.48 (1.19, 1.82) |
| √ | | | √ | 59 | 1,241,291 | 130,234 | 10,502 | 1.48 (1.35, 1.62) | 85,810 | 1.00 (0.97, 1.03) | 29,913 | 1.08 (1.06, 1.10) | 3,564 | 1.47 (1.38, 1.56) | 314 | 1.68 (1.44, 1.95) | 131 | 1.59 (1.29, 1.96) |
| √ | | √ | √ | 46 | 1,074,385 | 100,784 | 7,178 | 1.40 (1.25, 1.57) | 66,498 | 1.00 (0.97, 1.03) | 24,020 | 1.12 (1.10, 1.14) | 2,753 | 1.53 (1.43, 1.63) | 231 | 1.71 (1.44, 2.03) | 104 | 1.62 (1.26, 2.10) |
| **South Asia** | | | | | | | | | | | | | | | | | | |
| | | | | 3 | 280,228 | 22,250 | 6,316 | 1.39 (1.22, 1.58) | 12,466 | 1.00 (0.99, 1.01) | 2,881 | 0.86 (0.82, 0.90) | 490 | 0.94 (0.84, 1.05) | 72 | 1.08 (0.85, 1.37) | 25 | 1.95 (1.31, 2.90) |
| √ | | | | 3 | 274,575 | 21,085 | 5,823 | 1.40 (1.22, 1.60) | 11,896 | 1.00 (0.99, 1.01) | 2,798 | 0.86 (0.81, 0.91) | 474 | 0.93 (0.83, 1.04) | 70 | 1.06 (0.83, 1.37) | 24 | 1.97 (1.30, 2.97) |
| √ | √ | | | 3 | 274,575 | 21,085 | 5,823 | 1.36 (1.16, 1.58) | 11,896 | 1.00 (0.99, 1.01) | 2,798 | 0.87 (0.81, 0.94) | 474 | 0.94 (0.83, 1.05) | 70 | 1.06 (0.83, 1.37) | 24 | 1.97 (1.30, 2.98) |
| √ | √ | √ | | 3 | 218,549 | 6,002 | 1,644 | 1.27 (1.06, 1.52) | 3,383 | 1.00 (0.99, 1.01) | 809 | 0.93 (0.81, 1.07) | 136 | 1.01 (0.85, 1.20) | 24 | 1.38 (0.92, 2.07) | 6 | 1.53 (0.68, 3.41) |
| √ | | | √ | 3 | 217,862 | 14,267 | 3,491 | 1.36 (1.23, 1.50) | 8,182 | 1.00 (*) | 2,122 | 0.87 (0.81, 0.93) | 390 | 0.93 (0.82, 1.04) | 62 | 1.02 (0.61, 1.69) | 20 | 1.97 (1.01, 3.83) |
| √ | | √ | √ | 3 | 176,781 | 4,040 | 964 | 1.28 (1.11, 1.46) | 2,306 | 1.00 (0.99, 1.01) | 629 | 0.93 (0.80, 1.09) | 114 | 0.99 (0.81, 1.19) | 22 | 1.42 (0.93, 2.16) | 5 | 1.57 (0.65, 3.79) |

All analyses are standardised for baseline age and sex. Reference category is WHO-defined normal weight (BMI 18.5 to <25 kg/m$^2$).

Group-specific CIs were calculated using floating variance estimates (reflecting independent variability within each group, including the reference group).

*The penultimate analysis of the South Asian studies used standard CIs rather than floating absolute risks.

**eTable 5: Six WHO BMI groups vs all-cause mortality, by successively stricter inclusion criteria, in the 189 studies contributing to all such analyses (including the strictest, which is the pre-specified primary analysis)**

| Exclude baseline chronic disease? | Adjust for smoking? | Exclude the first 5 years of follow-up? | Restrict analyses to never-smokers? | No of studies | No of partici-pants | No of deaths | Underweight (BMI 15 to <18.5) | | Normal weight (18.5 to <25) | | Overweight (25 to <30) | | Obesity Grade I (30 to <35) | | Obesity Grade II (35 to <40) | | Obesity Grade III (40 to <60) | |
|---|---|---|---|---|---|---|---|---|---|---|---|---|---|---|---|---|---|---|
| | | | | | | | No of deaths | HR (95% CI) | No of deaths | HR (95% CI) | No of deaths | HR (95% CI) | No of deaths | HR (95% CI) | No of deaths | HR (95% CI) | No of deaths | HR (95% CI) |
| | | | | 189 | 10,097,300 | 1,569,327 | 67,412 | 1.81 (1.72, 1.91) | 796,151 | 1.00 (0.98, 1.02) | 513,629 | 0.96 (0.95, 0.97) | 141,514 | 1.18 (1.17, 1.19) | 35,434 | 1.50 (1.47, 1.52) | 15,187 | 1.96 (1.90, 2.02) |
| √ | | | | 189 | 8,613,459 | 1,183,235 | 51,921 | 1.80 (1.70, 1.90) | 618,867 | 1.00 (0.98, 1.02) | 378,600 | 0.96 (0.95, 0.97) | 99,695 | 1.18 (1.17, 1.20) | 24,009 | 1.51 (1.48, 1.55) | 10,143 | 2.04 (1.97, 2.12) |
| √ | √ | | | 189 | 8,411,814 | 1,164,433 | 50,585 | 1.68 (1.58, 1.78) | 609,250 | 1.00 (0.98, 1.02) | 373,294 | 0.99 (0.98, 1.01) | 97,958 | 1.25 (1.23, 1.27) | 23,489 | 1.62 (1.58, 1.66) | 9,857 | 2.21 (2.13, 2.30) |
| √ | √ | √ | | 189 | 7,706,287 | 936,304 | 33,654 | 1.59 (1.49, 1.69) | 491,157 | 1.00 (0.98, 1.02) | 306,933 | 1.03 (1.01, 1.04) | 78,567 | 1.31 (1.29, 1.32) | 18,426 | 1.69 (1.66, 1.73) | 7,567 | 2.34 (2.25, 2.44) |
| √ | | | √ | 189 | 4,290,799 | 479,530 | 19,937 | 1.51 (1.43, 1.59) | 240,089 | 1.00 (0.98, 1.02) | 157,492 | 1.08 (1.07, 1.08) | 45,628 | 1.39 (1.36, 1.42) | 11,466 | 1.81 (1.76, 1.87) | 4,918 | 2.52 (2.38, 2.66) |
| √ | | √ | √ | 189 | 3,951,455 | 385,879 | 12,726 | 1.47 (1.39, 1.55) | 192,523 | 1.00 (0.98, 1.02) | 130,293 | 1.11 (1.10, 1.11) | 37,318 | 1.44 (1.41, 1.47) | 9,179 | 1.92 (1.86, 1.98) | 3,840 | 2.71 (2.55, 2.86) |

All analyses are standardised for baseline age and sex. Reference category is WHO-defined normal weight (BMI 18.5 to <25 kg/m$^2$).

Group-specific CIs were calculated using floating variance estimates (reflecting independent variability within each group, including the reference group).

**eTable 6: Six WHO BMI groups vs all-cause mortality, by baseline smoking status**

| | No of studies | No of participants | No of deaths | Underweight (BMI 15 to <18.5) | Normal weight (18.5 to <25) | Overweight (25 to <30) | Obesity Grade I (30 to <35) | Obesity Grade II (35 to <40) | Obesity Grade III (40 to <60) |
|---|---|---|---|---|---|---|---|---|---|
| Never-smokers | 98 | 440,289 | 45,480 | 1.53 (1.39, 1.68) | 1.00 (0.96, 1.04) | 1.07 (1.06, 1.07) | 1.39 (1.33, 1.44) | 1.86 (1.73, 1.99) | 2.69 (2.43, 2.97) |
| Ex- and current smokers | 98 | 564,671 | 97,853 | 1.86 (1.74, 1.99) | 1.00 (0.97, 1.03) | 0.94 (0.94, 0.95) | 1.13 (1.09, 1.17) | 1.51 (1.42, 1.61) | 2.04 (1.85, 2.24) |
| P value for heterogeneity between HRs in never-smokers and others | | | | 0.0458 | | 0.0003 | <0.0001 | 0.0004 | 0.0003 |

Analyses excluded the first 5 years of follow-up and people without pre-existing chronic disease.

Reference category is WHO-defined normal weight (BMI 18.5 to <25 $kg/m^2$).

Group-specific CIs were calculated using floating variance estimates (reflecting independent variability within each group, including the reference group).

**eTable 7: Six WHO BMI groups vs all-cause mortality, using standard CIs, not floating absolute risks**

| | No of studies | No of participants | No of deaths | Underweight (BMI 15 to <18.5) | Normal weight (18.5 to <25) | Overweight (25 to <30) | Obesity Grade I (30 to <35) | Obesity Grade II (35 to <40) | Obesity Grade III (40 to <60) |
|---|---|---|---|---|---|---|---|---|---|
| **All studies** | 189 | 3,951,455 | 385,879 | 1.47 (1.40, 1.55) | Reference | 1.11 (1.09, 1.13) | 1.44 (1.40, 1.49) | 1.92 (1.84, 2.00) | 2.71 (2.54, 2.88) |
| **Men** | 157 | 913,344 | 115,373 | 1.83 (1.66, 2.02) | Reference | 1.12 (1.09, 1.15) | 1.70 (1.62, 1.79) | 2.68 (2.53, 2.84) | 4.24 (3.77, 4.77) |
| **Women** | 141 | 2,743,371 | 264,657 | 1.53 (1.45, 1.60) | Reference | 1.08 (1.06, 1.11) | 1.37 (1.32, 1.42) | 1.86 (1.77, 1.95) | 2.73 (2.55, 2.93) |

Analyses excluded the first 5 years of follow-up, restricted to never-smokers without pre-existing chronic disease. Reference category is WHO-defined normal weight (BMI 18.5 to <25 kg/$m^2$).

**eTable 8: Nine BMI groups vs all-cause mortality, by geographic region**

| | Body-mass index (kg/m²) | | | | | | | | |
|---|---|---|---|---|---|---|---|---|---|
| | BMI 15 to <18.5 | 18.5 to <20 | 20 to <22.5 | 22.5 to <25 | 25 to <27.5 | 27.5 to <30 | 30 to <35 | 35 to <40 | 40 to <60 |
| **All geographic regions** (189 studies; 3,951,455 participants; 385,879 deaths) | | | | | | | | | |
| No. participants / deaths | 114091 / 12726 | 230749 / 20989 | 838907 / 72701 | 1075894/98833 | 821303 / 84952 | 428800 / 45341 | 330840 / 37318 | 80827 / 9179 | 30044 / 3840 |
| HR (95% CI) | 1.51 (1.43, 1.59) | 1.13 (1.09, 1.17) | 1.00 (0.98, 1.02) | 1.00 (0.99, 1.01) | 1.07 (1.07, 1.08) | 1.20 (1.18, 1.22) | 1.45 (1.41, 1.48) | 1.94 (1.87, 2.01) | 2.76 (2.60, 2.92) |
| $I^2$ (95% CI) | 64% (58%, 69%) | 43% (32%, 52%) | 10% (0%, 25%) | . | 11% (0%, 27%) | 33% (20%, 44%) | 53% (45%, 60%) | 31% (17%, 42%) | 36% (23%, 47%) |
| **Europe** (89 studies; 1,135,600 participants; 56,477 deaths) | | | | | | | | | |
| No. participants / deaths | 13398 / 675 | 42584 / 1508 | 199369 / 7449 | 306566 / 13278 | 249929 / 12850 | 153147 / 8935 | 127536 / 8386 | 32749 / 2424 | 10322 / 972 |
| HR (95% CI) | 1.79 (1.63, 1.97) | 1.25 (1.14, 1.38) | 1.02 (0.97, 1.07) | 1.00 (0.97, 1.03) | 1.07 (1.06, 1.09) | 1.21 (1.18, 1.25) | 1.52 (1.45, 1.58) | 1.99 (1.87, 2.12) | 3.04 (2.84, 3.27) |
| $I^2$ (95% CI) | 0% (0%, 26%) | 23% (0%, 42%) | 0% (0%, 26%) | . | 5% (0%, 28%) | 19% (0%, 39%) | 51% (38%, 62%) | 24% (0%, 42%) | 0% (0%, 26%) |
| **North America** (40 studies; 1,415,087 participants; 219,922 deaths) | | | | | | | | | |
| No. participants / deaths | 22028 / 3846 | 67114 / 8597 | 274883 / 36200 | 359022 / 54995 | 317721 / 53464 | 168183 / 28471 | 149807 / 25348 | 39379 / 6299 | 16950 / 2702 |
| HR (95% CI) | 1.51 (1.34, 1.70) | 1.09 (1.02, 1.16) | 1.01 (0.96, 1.06) | 1.00 (0.97, 1.03) | 1.06 (1.04, 1.07) | 1.17 (1.12, 1.22) | 1.39 (1.30, 1.49) | 1.93 (1.74, 2.13) | 2.58 (2.26, 2.93) |
| $I^2$ (95% CI) | 40% (12%, 59%) | 13% (0%, 41%) | 6% (0%, 35%) | . | 9% (0%, 38%) | 55% (36%, 69%) | 70% (59%, 78%) | 65% (51%, 75%) | 63% (48%, 74%) |
| **Australia / NZ** (11 studies; 149,602 participants; 4,656 deaths) | | | | | | | | | |
| No. participants / deaths | 1592 / 63 | 4620 / 135 | 20698 / 530 | 35234 / 1027 | 34479 / 1121 | 23823 / 803 | 21070 / 717 | 5884 / 203 | 2202 / 57 |
| HR (95% CI) | 1.45 (1.12, 1.90) | 1.29 (0.96, 1.74) | 1.02 (0.91, 1.14) | 1.00 (0.94, 1.06) | 0.98 (0.93, 1.04) | 1.11 (0.99, 1.25) | 1.31 (1.20, 1.42) | 1.81 (1.55, 2.11) | 2.03 (1.49, 2.77) |
| $I^2$ (95% CI) | 0% (0%, 60%) | 28% (0%, 64%) | 0% (0%, 60%) | . | 0% (0%, 60%) | 0% (0%, 60%) | 0% (0%, 60%) | 0% (0%, 60%) | 0% (0%, 60%) |
| **East Asia** (46 studies; 1,074,385 participants; 100,784 deaths) | | | | | | | | | |
| No. participants / deaths | 46979 / 7178 | 94409 / 10206 | 301242 / 27537 | 336758 / 28755 | 194857 / 17070 | 72133 / 6950 | 25658 / 2753 | 1941 / 231 | 408 / 104 |
| HR (95% CI) | 1.36 (1.25, 1.49) | 1.11 (1.04, 1.18) | 0.99 (0.97, 1.02) | 1.00 (0.97, 1.03) | 1.07 (1.04, 1.11) | 1.28 (1.21, 1.35) | 1.54 (1.42, 1.67) | 2.01 (1.59, 2.54) | 2.38 (1.33, 4.24) |
| $I^2$ (95% CI) | 86% (82%, 89%) | 67% (55%, 76%) | 13% (0%, 40%) | . | 19% (0%, 44%) | 0% (0%, 34%) | 24% (0%, 48%) | 0% (0%, 34%) | 7% (0%, 35%) |
| **South Asia** (3 studies; 176,781 participants; 4,040 deaths) | | | | | | | | | |
| No. participants / deaths | 30094 / 964 | 22022 / 543 | 42715 / 985 | 38314 / 778 | 24317 / 447 | 11514 / 182 | 6769 / 114 | 874 / 22 | 162 / 5 |
| HR (95% CI) | 1.30 (1.10, 1.52) | 1.08 (0.90, 1.29) | 1.04 (0.96, 1.13) | 1.00 (0.89, 1.13) | 0.99 (0.83, 1.19) | 0.94 (0.72, 1.24) | 1.03 (0.85, 1.26) | 1.44 (0.94, 2.20) | 1.62 (0.67, 3.91) |
| $I^2$ (95% CI) | 0% (0%, 90%) | 54% (0%, 87%) | 0% (0%, 90%) | . | 45% (0%, 84%) | 59% (0%, 88%) | 0% (0%, 90%) | 0% (0%, 90%) | 0% (0%, 90%) |

Analyses excluded the first 5 years of follow-up, and are restricted to never-smokers without pre-existing chronic disease.

Reference category is BMI 22.5 to <25 kg/m².

Group-specific CIs were calculated using floating variance estimates (reflecting independent variability within each group, including the reference group).

## eTable 9: Six WHO BMI groups vs all-cause mortality, by geographic region

| | Underweight (BMI 15 to <18.5) | Normal weight (18.5 to <25) | Overweight (25 to <30) | Obesity Grade I (30 to <35) | Obesity Grade II (35 to <40) | Obesity Grade III (40 to <60) |
|---|---|---|---|---|---|---|
| **Europe** | | | | | | |
| (89 studies; 1,135,600 participants; 56,477 deaths) | | | | | | |
| No participants / deaths | 13398 / 675 | 548519 / 22235 | 403076 / 21785 | 127536 / 8386 | 32749 / 2424 | 10322 / 972 |
| HR (95% CI) | 1.69 (1.56, 1.85) | 1.00 (0.96, 1.04) | 1.12 (1.11, 1.13) | 1.51 (1.45, 1.56) | 2.01 (1.87, 2.17) | 3.00 (2.80, 3.22) |
| $I^2$ (95% CI) | 0% (0%, 26%) | . | 33% (13%, 49%) | 60% (49%, 68%) | 31% (9%, 47%) | 0% (0%, 26%) |
| **North America** | | | | | | |
| (40 studies; 1,415,087 participants; 219,922 deaths) | | | | | | |
| No participants / deaths | 22028 / 3846 | 701019 / 99792 | 485904 / 81935 | 149807 / 25348 | 39379 / 6299 | 16950 / 2702 |
| HR (95% CI) | 1.44 (1.33, 1.55) | 1.00 (0.97, 1.03) | 1.10 (1.08, 1.12) | 1.40 (1.35, 1.44) | 1.96 (1.84, 2.08) | 2.64 (2.41, 2.88) |
| $I^2$ (95% CI) | 37% (7%, 57%) | . | 66% (53%, 76%) | 80% (73%, 85%) | 71% (61%, 79%) | 69% (57%, 78%) |
| **Australia / NZ** | | | | | | |
| (11 studies; 149,602 participants; 4656 deaths) | | | | | | |
| No participants / deaths | 1592 / 63 | 60552 / 1692 | 58302 / 1924 | 21070 / 717 | 5884 / 203 | 2202 / 57 |
| HR (95% CI) | 1.41 (1.10, 1.82) | 1.00 (0.96, 1.04) | 1.01 (0.93, 1.08) | 1.28 (1.18, 1.40) | 1.76 (1.53, 2.03) | 2.11 (1.62, 2.74) |
| $I^2$ (95% CI) | 0% (0%, 60%) | . | 0% (0%, 60%) | 0% (0%, 60%) | 0% (0%, 60%) | 0% (0%, 60%) |
| **East Asia** | | | | | | |
| (46 studies; 1,074,385 participants; 100,784 deaths) | | | | | | |
| No participants / deaths | 46979 / 7178 | 732409 / 66498 | 266990 / 24020 | 25658 / 2753 | 1941 / 231 | 408 / 104 |
| HR (95% CI) | 1.40 (1.25, 1.57) | 1.00 (0.97, 1.03) | 1.12 (1.10, 1.14) | 1.53 (1.43, 1.63) | 1.71 (1.44, 2.03) | 1.62 (1.26, 2.10) |
| $I^2$ (95% CI) | 85% (81%, 89%) | . | 53% (34%, 66%) | 38% (11%, 57%) | 0% (0%, 34%) | 4% (0%, 30%) |
| **South Asia** | | | | | | |
| (3 studies; 176,781 participants; 4040 deaths) | | | | | | |
| No participants / deaths | 30094 / 964 | 103051 / 2306 | 35831 / 629 | 6769 / 114 | 874 / 22 | 162 / 5 |
| HR (95% CI) | 1.28 (1.11, 1.46) | 1.00 (0.99, 1.01) | 0.93 (0.80, 1.09) | 0.99 (0.81, 1.19) | 1.42 (0.93, 2.16) | 1.57 (0.65, 3.79) |
| $I^2$ (95% CI) | 26% (0%, 92%) | . | 35% (0%, 79%) | 0% (0%, 90%) | 0% (0%, 90%) | 0% (0%, 90%) |
| **P-value for heterogeneity by region** | 0.0526 | - | 0.3980 | 0.0152 | 0.5994 | 0.0005 |

Analyses excluded the first 5 years of follow-up, restricted to never-smokers without pre-existing chronic disease.

P-values for difference were calculated using multivariate meta-regression.

Reference category is WHO-defined normal weight (BMI 18.5 to <25 kg/m$^2$).

Group-specific CIs were calculated using floating variance estimates (reflecting independent variability within each group, including the reference group).

## eTable 10: Six WHO BMI groups vs all-cause mortality, by geographic region and by sex

| | Underweight (BMI 15 to <18.5) | Normal weight (18.5 to <25) | Overweight (25 to <30) | Obesity Grade I (30 to <35) | Obesity Grade II (35 to <40) | Obesity Grade III (40 to <60) |
|---|---|---|---|---|---|---|
| **All geographic regions** | | | | | | |
| **Men** (157 studies; 913,344 participants; 115,373 deaths) | | | | | | |
| No of participants / deaths | 20172 / 2761 | 458050 / 54707 | 353791 / 46465 | 67782 / 9485 | 10640 / 1532 | 2909 / 423 |
| HR (95% CI) | 1.83 (1.66, 2.02) | 1.00 (0.98, 1.02) | 1.12 (1.11, 1.13) | 1.70 (1.63, 1.77) | 2.68 (2.54, 2.83) | 4.24 (3.77, 4.76) |
| **Women** (141 studies; 2,743,371 participants; 264,657 deaths) | | | | | | |
| No of participants / deaths | 81728 / 9637 | 1513911 / 134546 | 819776 / 82231 | 239900 / 27393 | 63773 / 7524 | 24283 / 3326 |
| HR (95% CI) | 1.53 (1.44, 1.62) | 1.00 (0.98, 1.02) | 1.08 (1.07, 1.10) | 1.37 (1.34, 1.40) | 1.86 (1.79, 1.93) | 2.73 (2.57, 2.91) |
| **Europe** | | | | | | |
| **Men** (75 studies; 166,935 participants; 14,373 deaths) | | | | | | |
| No of participants / deaths | 761 / 93 | 72953 / 5412 | 76276 / 6743 | 14987 / 1832 | 1658 / 238 | 300 / 55 |
| HR (95% CI) | 2.17 (1.76, 2.68) | 1.00 (0.96, 1.04) | 1.17 (1.16, 1.18) | 1.91 (1.77, 2.05) | 3.38 (2.96, 3.86) | 7.83 (5.93, 10.35) |
| **Women** (59 studies; 819,235 participants; 41,254 deaths) | | | | | | |
| No of participants / deaths | 8131 / 552 | 389654 / 16337 | 284478 / 14800 | 100031 / 6484 | 28045 / 2173 | 8896 / 908 |
| HR (95% CI) | 2.00 (1.67, 2.40) | 1.00 (0.96, 1.04) | 1.06 (1.04, 1.08) | 1.31 (1.26, 1.37) | 1.87 (1.73, 2.03) | 2.92 (2.70, 3.17) |
| **North America** | | | | | | |
| **Men** (30 studies;352,288 participants; 64,859 deaths) | | | | | | |
| No of participants / deaths | 1786 / 476 | 128348 / 24420 | 173053 / 31613 | 39832 / 6817 | 7168 / 1188 | 2101 / 345 |
| HR (95% CI) | 2.13 (1.70, 2.67) | 1.00 (0.97, 1.03) | 1.11 (1.10, 1.12) | 1.55 (1.49, 1.61) | 2.52 (2.29, 2.78) | 3.72 (3.17, 4.37) |
| **Women** (33 studies; 946,179 participants; 150,583 deaths) | | | | | | |
| No of participants / deaths | 16220 / 3141 | 504025 / 72940 | 283505 / 49048 | 100226 / 18175 | 29015 / 5002 | 13188 / 2277 |
| HR (95% CI) | 1.49 (1.36, 1.65) | 1.00 (0.96, 1.04) | 1.09 (1.07, 1.11) | 1.35 (1.31, 1.40) | 1.91 (1.80, 2.03) | 2.53 (2.35, 2.73) |
| **Australia / NZ** | | | | | | |
| **Men** (11 studies; 55,340 participants; 2,141 deaths) | | | | | | |
| No of participants | 214 / 15 | 18309 / 731 | 27002 / 1016 | 7843 / 301 | 1543 / 66 | 429 / 12 |
| HR (95% CI) | 2.35 (1.40, 3.95) | 1.00 (0.94, 1.06) | 0.98 (0.82, 1.17) | 1.36 (1.10, 1.69) | 2.79 (2.17, 3.60) | 4.46 (2.48, 8.02) |
| **Women** (9 studies; 88,284 participants; 2,450 deaths) | | | | | | |
| No of participants | 1251 / 47 | 38495 / 922 | 29697 / 888 | 12849 / 411 | 4245 / 137 | 1747 / 45 |
| HR (95% CI) | 1.91 (1.42, 2.58) | 1.00 (0.95, 1.06) | 1.00 (0.91, 1.10) | 1.34 (1.19, 1.52) | 1.58 (1.33, 1.88) | 2.45 (1.82, 3.29) |
| **East Asia** | | | | | | |
| **Men** (39 studies; 281,530 participants; 32,355 deaths) | | | | | | |
| No of participants | 9669 / 1831 | 201325 / 23132 | 66543 / 6850 | 3765 / 499 | 162 / 32 | - |
| HR (95% CI) | 1.45 (1.28, 1.65) | 1.00 (0.96, 1.04) | 1.12 (1.06, 1.17) | 2.02 (1.71, 2.39) | 3.12 (2.20, 4.43) | - |
| **Women** (37 studies; 778,840 participants; 68,006 deaths) | | | | | | |
| No of participants | 35868 / 5288 | 521018 / 43068 | 198358 / 17114 | 21568 / 2247 | 1716 / 198 | - |
| HR (95% CI) | 1.39 (1.30, 1.49) | 1.00 (0.98, 1.02) | 1.11 (1.08, 1.15) | 1.56 (1.43, 1.71) | 2.40 (1.89, 3.04) | - |

Analyses excluded the first 5 years of follow-up, and were restricted to never-smokers without pre-existing chronic disease.
Reference category is WHO-defined normal weight (BMI value 18.5 to <25 kg/m$^2$).
CIs were calculated using floating variance estimates (reflecting independent variability within each group, including the reference group).
Results for South Asia and highest BMI category in East Asia were not shown as there were too few events to estimate sex-specific HRs reliably.

## eTable 11: Six WHO BMI groups vs all-cause mortality, by geographic region and baseline age

| | Underweight (BMI 15 to <18.5) | Normal weight (18.5 to <25) | Overweight (25 to <30) | Obesity Grade I (30 to <35) | Obesity Grade II (35 to <40) | Obesity Grade III (40 to <60) |
|---|---|---|---|---|---|---|
| **All geographic regions** | | | | | | |
| Age 35-49 years (124 studies, 1,206,420 participants, 42,531 deaths) | | | | | | |
| No of participants | 42933 / 1214 | 749003 / 22615 | 323176 / 12958 | 68994 / 3947 | 16000 / 1162 | 6314 / 635 |
| HR (95% CI) | 1.46 (1.35, 1.58) | 1.00 (0.96, 1.04) | 1.20 (1.17, 1.24) | 1.86 (1.79, 1.95) | 2.86 (2.66, 3.09) | 4.67 (4.27, 5.10) |
| Age 50-69 years (167 studies, 2,205,216 participants, 246,629 deaths) | | | | | | |
| No of participants | 47216 / 6424 | 1094744 / 117156 | 770657 / 87401 | 218503 / 26230 | 54459 / 6686 | 19637 / 2732 |
| HR (95% CI) | 1.56 (1.39, 1.75) | 1.00 (0.98, 1.02) | 1.11 (1.09, 1.12) | 1.50 (1.46, 1.53) | 2.16 (2.07, 2.26) | 3.14 (2.95, 3.34) |
| Age 70-89 years (88 studies, 225,490 participants, 90,722 deaths) | | | | | | |
| No of participants | 11419 / 4744 | 118708 / 49413 | 72488 / 28297 | 18253 / 6682 | 3517 /1205 | 1105 / 381 |
| HR (95% CI) | 1.38 (1.30, 1.46) | 1.00 (0.98, 1.02) | 1.00 (0.98, 1.02) | 1.19 (1.14, 1.23) | 1.64 (1.48, 1.82) | 1.75 (1.57, 1.95) |
| **Europe** | | | | | | |
| Age 35-49 years (55 studies, 226,459 participants, 7752 deaths) | | | | | | |
| No of participants | 3002 / 80 | 124652 / 3469 | 75707 / 3000 | 18135 / 911 | 3861 / 220 | 1102 / 72 |
| HR (95% CI) | 1.86 (1.49, 2.33) | 1.00 (0.94, 1.06) | 1.17 (1.15, 1.20) | 1.90 (1.72, 2.09) | 3.01 (2.62, 3.46) | 5.34 (4.20, 6.78) |
| Age 50-69 years (79 studies, 727,044 participants, 41,041 deaths) | | | | | | |
| No of participants | 5441 / 440 | 324186 / 15542 | 271692 / 15755 | 92792 / 6435 | 25028 / 2013 | 7905 / 856 |
| HR (95% CI) | 2.25 (1.97, 2.56) | 1.00 (0.96, 1.04) | 1.11 (1.07, 1.15) | 1.60 (1.51, 1.70) | 2.23 (2.06, 2.40) | 4.04 (3.58, 4.55) |
| Age 70-89 years (28 studies, 23,613 participants, 6,775 deaths) | | | | | | |
| No of participants | 346 / 123 | 9714 / 2711 | 9804 / 2772 | 3056 / 957 | 575 / 177 | 118 / 35 |
| HR (95% CI) | 1.65 (1.31, 2.08) | 1.00 (0.92, 1.09) | 0.98 (0.93, 1.02) | 1.12 (1.03, 1.21) | 1.56 (1.25, 1.95) | 1.91 (1.35, 2.71) |
| **North America** | | | | | | |
| Age 35-49 years (26 studies, 390,431 participants, 19,811 deaths) | | | | | | |
| No of participants | 6560 / 299 | 217529 / 8799 | 116454 / 6623 | 35414 / 2647 | 9984 / 897 | 4490 / 546 |
| HR (95% CI) | 1.43 (1.20, 1.71) | 1.00 (0.93, 1.07) | 1.27 (1.26, 1.29) | 1.85 (1.76, 1.95) | 2.60 (2.28, 2.98) | 4.01 (3.48, 4.63) |
| Age 50-69 years (36 studies, 794,285 participants, 139,428 deaths) | | | | | | |
| No of participants | 8820 / 1804 | 358849 / 59630 | 299389 / 54405 | 93372 / 17447 | 23861 / 4360 | 9994 / 1782 |
| HR (95% CI) | 1.39 (0.95, 2.05) | 1.00 (0.97, 1.03) | 1.13 (1.10, 1.16) | 1.51 (1.47, 1.56) | 2.15 (2.04, 2.28) | 2.87 (2.68, 3.08) |
| Age 70-89 years (28 studies, 111,757 participants, 56,180 deaths) | | | | | | |
| No of participants | 2587 / 1514 | 55030 / 28920 | 40056 / 19625 | 11047 / 4896 | 2267 / 931 | 770 / 294 |
| HR (95% CI) | 1.45 (1.33, 1.58) | 1.00 (0.98, 1.02) | 0.99 (0.96, 1.02) | 1.17 (1.12, 1.22) | 1.71 (1.47, 1.98) | 1.79 (1.54, 2.07) |

Notes are at the end of eTable 11, on page 24.

## eTable 11 (continued)

| | Underweight (BMI 15 to <18.5) | Normal weight (18.5 to <25) | Overweight (25 to <30) | Obesity Grade I (30 to <35) | Obesity Grade II (35 to <40) | Obesity Grade III (40 to <60) |
|---|---|---|---|---|---|---|
| **Australia / NZ** | | | | | | |
| Age 35-49 years (6 studies, 28,449 participants, 275 deaths) | | | | | | |
| No of participants | . | 12629 / 121 | 10323 / 99 | 3593 / 38 | 1093 / 11 | 508 / 6 |
| HR (95% CI) | . | 1.00 (0.87, 1.15) | 1.05 (0.77, 1.45) | 1.76 (1.23, 2.51) | 3.30 (1.56, 6.99) | 4.63 (1.99, 10.77) |
| Age 50-69 years (9 studies, 89,893 participants, 2,522 deaths) | | | | | | |
| No of participants | 762 / 21 | 33345 / 815 | 36229 / 1082 | 14022 / 423 | 4050 / 146 | 1485 / 35 |
| HR (95% CI) | 3.47 (1.93, 6.24) | 1.00 (0.91, 1.10) | 1.05 (1.00, 1.11) | 1.38 (1.16, 1.64) | 2.05 (1.58, 2.66) | 2.75 (1.90, 3.98) |
| Age 70-89 years (9 studies, 21,191participants, 1,782 deaths) | | | | | | |
| No of participants | 357 / 41 | 9056 / 711 | 8496 / 721 | 2593 / 248 | 535 / 46 | 154 / 15 |
| HR (95% CI) | 1.90 (1.15, 3.12) | 1.00 (0.93, 1.08) | 0.97 (0.89, 1.05) | 1.31 (1.14, 1.51) | 1.74 (1.19, 2.53) | 2.14 (0.99, 4.64) |
| **East Asia** | | | | | | |
| Age 35-49 years (34 studies, 465,574 participants, 14,070 deaths) | | | | | | |
| No of participants | 17568 / 698 | 338759 / 9862 | 100473 / 3140 | 8056 / 330 | 588 / 31 | 130 / 9 |
| HR (95% CI) | 1.60 (1.33, 1.91) | 1.00 (0.93, 1.07) | 1.24 (1.17, 1.31) | 2.16 (1.85, 2.52) | 3.49 (2.45, 4.98) | 3.63 (1.88, 6.99) |
| Age 50-69 years (41 studies, 531,345participants, 61,511deaths) | | | | | | |
| No of participants | 22069 / 3691 | 341802 / 39963 | 150351 / 15789 | 15763 / 1859 | 1170 / 153 | 190 / 56 |
| HR (95% CI) | 1.39 (1.25, 1.54) | 1.00 (0.98, 1.02) | 1.11 (1.07, 1.16) | 1.61 (1.46, 1.79) | 2.27 (1.83, 2.82) | 2.27 (1.61, 3.21) |
| Age 70-89 years (21 studies, 58,715participants, 24,728deaths) | | | | | | |
| No of participants | 5710 / 2716 | 38866 / 16353 | 12660 / 5020 | 1320 / 556 | 103 / 46 | 56 / 37 |
| HR (95% CI) | 1.30 (1.15, 1.47) | 1.00 (0.98, 1.02) | 1.00 (0.92, 1.08) | 1.24 (1.13, 1.36) | 1.51 (1.12, 2.05) | 1.56 (1.11, 2.17) |

Analyses excluded the first 5 years of follow-up, restricted to never-smokers without pre-existing chronic disease.

Reference categories are participants with normal weight (BMI 18.5 to <25 kg/m$^2$).

CIs were calculated using floating variance estimates (reflecting independent variability within each group, including the reference group).

Results for the lowest BMI category in Australia / NZ for baseline age 35-49 years were not shown because there were too few events to estimate age-specific HRs reliably.

Results for all BMI categories in South Asian studies were not shown because there were not enough events to estimate age-specific HRs reliably.

**eTable 12: Population-attribuable fractions (PAFs) for adult mortality associated with overweight and obesity**

PAF = proportion of all adult mortality that would be avoided if the individuals who are overweight (BMI 25 to <30 kg/m$^2$) or obese (BMI 30 to ≤60 kg/m$^2$) had the mortality rates of those in the WHO-defined normal range of BMI (18.5 to <25 kg/m$^2$)

| | (a) PAFs within the Global BMI Collaboration studies (with lower prevalences of adiposity than nowadays) | | | (b) PAFs using contemporary prevalence estimates and HRs from all regions* | |
|---|---|---|---|---|---|
| | HR (95% CI) | Within-study prevalence | Within-study PAF (%) | Contemporary prevalence | Contemporary PAF (%) |
| **North America** | | | | | |
| Overweight | 1.10 (1.08, 1.12) | 34% | 3.0% (2.0%, 4.0%) | 38% | 3.2% |
| Obese | 1.59 (1.55, 1.64) | 15% | 7.8% (7.1%, 8.5%) | 31% | 15.9% |
| **Australia/New Zealand** | | | | | |
| Overweight | 1.01 (0.93, 1.08) | 39% | 0.2% (0.0%, 3.2%) | 37% | 3.3% |
| Obese | 1.44 (1.34, 1.54) | 19% | 7.9% (6.0%, 9.8%) | 25% | 13.2% |
| **Europe** | | | | | |
| Overweight | 1.12 (1.11, 1.13) | 36% | 3.6% (2.5%, 4.8%) | 33% | 3.0% |
| Obese | 1.79 (1.74, 1.85) | 15% | 9.0% (8.2%, 9.9%) | 20% | 10.5% |
| **East Asia** | | | | | |
| Overweight | 1.12 (1.10, 1.14) | 25% | 2.8% (1.8%, 3.7%) | 19% | 1.9% |
| Obese | 1.55 (1.46, 1.64) | 3% | 1.3% (1.1%, 1.6%) | 6% | 2.8% |

The hazard ratios (HRs) in this table involve comparisons versus the WHO-defined normal range (BMI 18.5 to <25.0 kg/m$^2$); comparisons vs the minimum-mortality range (BMI 20 to <25 kg/m$^2$) would increase these HRs only slightly (by ~0.01or 0.02).

HRs for all obesity were derived from the HRs in eTable 9 for obesity grade I, II and III, using inverse-variance weights.
Results for South Asia are not shown separately because there were too few events to estimate the obesity HR reliably.

* The HRs (and their 95% CIs) from our meta-analyses of all regions were 1.11 (1.10, 1.11) for overweight and 1.64 (1.61, 1.67) for obese.
The regional prevalences of overweight and of obesity are from the Global Burden of Metabolic Risk Factors for Chronic Diseases Collaboration (Stevens G, Singh G et al. National, regional, and global trends in adult overweight and obesity prevalences. Popn Health Metrics 2012;10(1):22).

**eTable 13: All-cause mortality HR per 5 kg/m$^2$ higher BMI in the BMI range above 25 kg/m$^2$ overall; by sex; and by baseline age**

| | All regions | Europe | North America | East Asia | Australia / NZ | South Asia |
|---|---|---|---|---|---|---|
| **Overall (both sexes, all ages)** | | | | | | |
| No. studies/participants/deaths | 189 / 1691813 / 180630 | 89 / 573683 / 33567 | 40 / 692039 / 116284 | 46 / 294589 / 27004 | 11 / 85256 / 2844 | 3 / 43474 / 765 |
| HR per 5 units of BMI (95% CI) | 1.31 (1.29, 1.33) | 1.39(1.34, 1.43) | 1.29(1.26, 1.32) | 1.39 (1.34, 1.44) | 1.31 (1.27, 1.35) | 1.13 (0.97, 1.30) |
| **Male** | | | | | | |
| No. studies/participants/deaths | 157 / 435097 / 57897 | 75 / 93227 / 8870 | 30 / 222154 / 39963 | 39 / 70470 / 7381 | 11 / 36384 / 1380 | 2 / 12354 / 280 |
| HR per 5 units of BMI (95% CI) | 1.51 (1.46, 1.56) | 1.70 (1.54, 1.88) | 1.43 (1.37, 1.50) | 1.77 (1.68, 1.88) | 1.51 (1.21, 1.90) | 1.64 (1.05, 2.55) |
| **Female** | | | | | | |
| No. studies/participants/deaths | 141 / 1147732 / 120474 | 59 / 421450 / 24365 | 33 / 425934 / 74502 | 37 / 221642 / 19559 | 9 / 46791 / 1436 | 3 / 29716 / 471 |
| HR per 5 units of BMI (95% CI) | 1.30 (1.26, 1.33) | 1.32 (1.26, 1.39) | 1.28 (1.25, 1.32) | 1.40 (1.29, 1.51) | 1.29 (1.19, 1.40) | 1.03 (0.84, 1.28) |
| **Baseline age 35-49** | | | | | | |
| No. studies/participants/deaths | 124 / 414484 / 18702 | 55 / 98805 / 4203 | 26 / 166342 / 10713 | 34 / 109117 / 3501 | 6 / 15009 / 148 | 3 / 24489 / 120 |
| HR per 5 units of BMI (95% CI) | 1.52 (1.47, 1.56) | 1.61 (1.58, 1.63) | 1.40 (1.37, 1.43) | 1.78 (1.72, 1.83) | 1.61 (1.41, 1.84) | 1.71 (1.23, 2.37) |
| **Baseline age 50-69** | | | | | | |
| No. studies/participants/deaths | 167 / 1063262 / 123051 | 80 / 398391 / 25701 | 36 / 426616 / 77994 | 41 / 167284 / 17801 | 9 / 54301 / 1651 | 2 / 15900 / 450 |
| HR per 5 units of BMI (95% CI) | 1.37 (1.35, 1.39) | 1.43 (1.37, 1.49) | 1.34 (1.31, 1.37) | 1.48 (1.45, 1.51) | 1.33 (1.25, 1.41) | 1.33 (0.95, 1.86) |
| **Baseline age 70-89** | | | | | | |
| No. studies/participants/deaths | 88 / 95332 / 36555 | 28 / 13553 / 3941 | 28 / 54140 / 25746 | 21 / 14083 / 5622 | 9 / 11620 / 1012 | 2 / 1719 / 182 |
| HR per 5 units of BMI (95% CI) | 1.21 (1.17, 1.25) | 1.21 (1.15, 1.29) | 1.20 (1.15, 1.26) | 1.26 (1.14, 1.39) | 1.39 (1.34, 1.45) | 1.03 (0.82, 1.30) |

Analyses excluded the first 5 years of follow-up, and are restricted to never-smokers without pre-existing chronic disease.

HRs per 5 units of BMI were estimated by inverse-variance-weighted regression of the pooled log HR on BMI in the BMI range 25 to 60 kg/m$^2$.

## eTable 14: Cause-specific mortality HR per 5 $kg/m^2$ higher BMI in the BMI range above 25 $kg/m^2$, by geographic region

| | All regions | Europe | North America | East Asia | Australia / NZ | South Asia |
|---|---|---|---|---|---|---|
| **All causes** | | | | | | |
| No. studies/participants/deaths | 189 / 1691813 / 180630 | 89 / 573683 / 33567 | 40 / 692039 / 116284 | 46 / 294589 / 27004 | 11 / 85256 / 2844 | 3 / 43474 / 765 |
| HR per 5 units of BMI (95% CI) | 1.31 (1.29, 1.33) | 1.39 (1.34, 1.43) | 1.29 (1.26, 1.32) | 1.39 (1.34, 1.44) | 1.31 (1.27, 1.35) | 1.13 (0.97, 1.30) |
| **Cardiovascular disease** | | | | | | |
| No. studies/participants/deaths | 164 / 1483133 / 66435 | 79 / 563075 / 11025 | 37 / 666326 / 50814 | 35 / 123322 / 3455 | 10 / 84262 / 769 | 3 / 43474 / 324 |
| HR per 5 units of BMI (95% CI) | 1.49 (1.45, 1.53) | 1.56 (1.54, 1.58) | 1.38 (1.35, 1.41) | 1.54 (1.39, 1.69) | 1.46 (1.26, 1.70) | 1.10 (0.83, 1.46) |
| **Coronary heart disease** | | | | | | |
| No. studies/participants/deaths | 124 / 1550175 / 30570 | 57 / 505350 / 5111 | 33 / 655408 / 23123 | 23 / 262007 / 1740 | 9 / 81859 / 397 | 2 / 42884 / 187 |
| HR per 5 units of BMI (95% CI) | 1.42 (1.35, 1.49) | 1.53 (1.48, 1.59) | 1.38 (1.32, 1.44) | 1.67 (1.50, 1.85) | 1.53 (1.20, 1.96) | 1.12 (0.76, 1.65) |
| **Stroke** | | | | | | |
| No. studies/participants/deaths | 114 / 1530488 / 17229 | 44 / 488239 / 2644 | 30 / 650176 / 10202 | 31 / 267714 / 4090 | 7 / 78843 / 184 | 2 / 42884 / 88 |
| HR per 5 units of BMI (95% CI) | 1.42 (1.35, 1.50) | 1.52 (1.35, 1.71) | 1.28 (1.20, 1.38) | 1.50 (1.31, 1.72) | 1.49 (1.24, 1.80) | 1.13 (0.87, 1.47) |
| **Respiratory disease** | | | | | | |
| No. studies/participants/deaths | 89 / 1411864 / 8321 | 31 / 462318 / 1209 | 27 / 626233 / 5645 | 24 / 263516 / 1315 | 5 / 16107 / 83 | 2 / 42884 / 52 |
| HR per 5 units of BMI (95% CI) | 1.38 (1.30, 1.47) | 1.63 (1.51, 1.76) | 1.32 (1.19, 1.46) | 1.49 (1.17, 1.91) | 1.56 (1.08, 2.27) | 1.56 (0.84, 2.89) |
| **Cancer** | | | | | | |
| No. studies/participants/deaths | 160 / 1656763 / 50890 | 73 / 564704 / 13520 | 38 / 690850 / 28452 | 37 / 271341 / 7999 | 10 / 84262 / 813 | 2 / 42884 / 76 |
| HR per 5 units of BMI (95% CI) | 1.19 (1.18, 1.20) | 1.21 (1.19, 1.23) | 1.19 (1.15, 1.23) | 1.25 (1.17, 1.33) | 1.19 (1.12, 1.26) | 0.87 (0.58, 1.30) |

Analyses excluded the first 5 years of follow-up, and were restricted to never-smokers without pre-existing chronic disease.

For Europe and North America, HRs per 5 units of BMI were estimated by inverse-variance-weighted regression of the pooled log HR on BMI in the BMI range 25 to <60 $kg/m^2$.

Elsewhere, there were too few events in the range 40 to 60 $kg/m^2$ to estimate HRs reliably, so the HRs per 5 units of BMI were estimated by inverse-variance-weighted regression of the pooled log HR on BMI in the more limited BMI range 25 to <40 $kg/m^2$.

## eTable 15: Nine BMI groups vs cause-specific mortality, by geographic region

| | No of studies | No of participants | No of deaths | Body-mass index (BMI, kg/m²) | | | | | | | | |
|---|---|---|---|---|---|---|---|---|---|---|---|---|
| | | | | 15 to <18.5 | 18.5 to <20 | 20 to <22.5 | 22.5 to <25 | 25 to <27.5 | 27.5 to <30 | 30 to <35 | 35 to <40 | 40 to <60 |
| **Cardiovascular disease** | | | | | | | | | | | | |
| All regions | 164 | 3276962 | 127586 | 1.33 (1.20, 1.47) | 1.14 (1.06, 1.22) | 0.98 (0.94, 1.03) | 1.00 (0.97, 1.03) | 1.11 (1.09, 1.14) | 1.35 (1.31, 1.40) | 1.75 (1.67, 1.84) | 2.73 (2.49, 2.99) | 4.03 (3.48, 4.67) |
| Europe | 79 | 1117915 | 16967 | 1.77 (1.52, 2.07) | 1.16 (1.04, 1.30) | 1.01 (0.93, 1.09) | 1.00 (0.95, 1.05) | 1.15 (1.10, 1.19) | 1.40 (1.33, 1.47) | 1.87 (1.76, 1.99) | 2.99 (2.68, 3.34) | 5.13 (4.53, 5.80) |
| North America | 37 | 1345640 | 93086 | 1.27 (1.18, 1.37) | 1.08 (1.01, 1.15) | 0.97 (0.92, 1.02) | 1.00 (0.97, 1.03) | 1.12 (1.11, 1.13) | 1.33 (1.28, 1.38) | 1.64 (1.54, 1.73) | 2.40 (2.15, 2.67) | 3.25 (2.88, 3.67) |
| Australia / NZ | 10 | 147994 | 1266 | 1.12 (0.55, 2.26) | 1.35 (0.94, 1.93) | 1.16 (0.95, 1.42) | 1.00 (0.88, 1.13) | 1.02 (0.91, 1.14) | 1.10 (0.95, 1.28) | 1.41 (1.21, 1.65) | 2.62 (1.93, 3.56) | 2.96 (1.40, 6.23) |
| East Asia | 35 | 488632 | 14871 | 1.13 (1.00, 1.27) | 1.02 (0.90, 1.15) | 0.92 (0.85, 0.98) | 1.00 (0.97, 1.03) | 1.08 (1.02, 1.14) | 1.35 (1.25, 1.46) | 1.87 (1.60, 2.19) | 2.36 (1.81, 3.06) | 1.06 (0.75, 1.51) |
| **Coronary heart disease** | | | | | | | | | | | | |
| All regions | 124 | 3599426 | 54872 | 1.10 (1.04, 1.17) | 0.95 (0.90, 1.01) | 0.89 (0.84, 0.93) | 1.00 (0.96, 1.04) | 1.18 (1.15, 1.20) | 1.45 (1.42, 1.49) | 1.89 (1.78, 2.00) | 2.61 (2.45, 2.79) | 3.54 (3.23, 3.88) |
| Europe | 57 | 1016359 | 7630 | 1.71 (1.31, 2.23) | 1.11 (0.94, 1.31) | 1.00 (0.90, 1.10) | 1.00 (0.93, 1.08) | 1.20 (1.14, 1.26) | 1.51 (1.43, 1.61) | 2.07 (1.89, 2.27) | 2.88 (2.57, 3.23) | 5.06 (4.26, 6.02) |
| North America | 33 | 1328311 | 40213 | 1.16 (1.03, 1.31) | 1.01 (0.91, 1.12) | 0.85 (0.80, 0.91) | 1.00 (0.95, 1.05) | 1.18 (1.17, 1.20) | 1.41 (1.32, 1.50) | 1.83 (1.67, 2.01) | 2.52 (2.21, 2.87) | 3.29 (2.85, 3.79) |
| Australia / NZ | 9 | 143673 | 648 | 1.80 (0.28, 11.42) | 1.31 (0.81, 2.11) | 1.11 (0.80, 1.53) | 1.00 (0.83, 1.20) | 1.00 (0.86, 1.18) | 1.04 (0.85, 1.28) | 1.33 (1.04, 1.69) | 2.95 (2.04, 4.26) | 2.77 (0.40, 19.3) |
| East Asia | 23 | 941039 | 5630 | 0.94 (0.82, 1.07) | 0.73 (0.64, 0.83) | 0.84 (0.77, 0.91) | 1.00 (0.95, 1.05) | 1.07 (0.97, 1.18) | 1.37 (1.23, 1.53) | 2.04 (1.55, 2.68) | 2.76 (1.73, 4.40) | 1.66 (0.79, 3.48) |
| **Stroke** | | | | | | | | | | | | |
| All regions | 114 | 3580423 | 40084 | 1.38 (1.16, 1.65) | 1.15 (1.04, 1.27) | 1.01 (0.96, 1.07) | 1.00 (0.97, 1.03) | 1.05 (1.00, 1.10) | 1.23 (1.15, 1.32) | 1.49 (1.38, 1.60) | 2.47 (2.10, 2.92) | 3.28 (2.39, 4.49) |
| Europe | 44 | 980210 | 4481 | 2.39 (1.82, 3.13) | 1.45 (1.22, 1.73) | 1.06 (0.96, 1.17) | 1.00 (0.92, 1.09) | 1.07 (0.99, 1.16) | 1.12 (1.02, 1.23) | 1.60 (1.39, 1.84) | 3.12 (2.37, 4.10) | 4.12 (3.19, 5.33) |
| North America | 30 | 1320458 | 20897 | 1.65 (1.24, 2.21) | 1.21 (1.04, 1.40) | 1.09 (0.99, 1.19) | 1.00 (0.94, 1.06) | 1.03 (0.96, 1.11) | 1.12 (1.03, 1.23) | 1.22 (1.09, 1.37) | 1.91 (1.61, 2.26) | 2.48 (1.92, 3.20) |
| Australia / NZ | 7 | 137292 | 308 | 4.41 (1.35, 14.40) | 2.41 (1.15, 5.06) | 1.16 (0.67, 1.99) | 1.00 (0.72, 1.39) | 0.91 (0.71, 1.16) | 1.27 (0.94, 1.70) | 1.35 (0.90, 2.03) | 2.27 (1.07, 4.83) | 5.42 (1.37, 21.5) |
| East Asia | 31 | 972419 | 13962 | 1.03 (0.89, 1.21) | 1.00 (0.92, 1.09) | 0.93 (0.87, 1.00) | 1.00 (0.98, 1.02) | 1.11 (1.04, 1.18) | 1.51 (1.38, 1.66) | 1.71 (1.56, 1.89) | 2.47 (1.84, 3.33) | 1.36 (0.84, 2.19) |
| **Respiratory disease** | | | | | | | | | | | | |
| All regions | 89 | 3353331 | 21634 | 3.26 (2.75, 3.86) | 1.73 (1.59, 1.88) | 1.22 (1.17, 1.27) | 1.00 (0.97, 1.03) | 1.00 (0.96, 1.03) | 1.10 (1.04, 1.15) | 1.30 (1.22, 1.39) | 2.03 (1.81, 2.27) | 3.27 (2.78, 3.83) |
| Europe | 31 | 923340 | 2238 | 5.99 (4.45, 8.08) | 2.17 (1.76, 2.68) | 1.44 (1.28, 1.63) | 1.00 (0.89, 1.12) | 0.97 (0.82, 1.14) | 1.06 (0.94, 1.19) | 1.49 (1.32, 1.68) | 2.68 (2.16, 3.33) | 4.51 (3.36, 6.07) |
| North America | 27 | 1278649 | 11912 | 2.72 (2.19, 3.36) | 1.43 (1.27, 1.62) | 1.11 (1.04, 1.18) | 1.00 (0.97, 1.03) | 0.91 (0.84, 1.00) | 1.07 (0.99, 1.15) | 1.01 (0.89, 1.15) | 1.52 (1.33, 1.74) | 2.77 (2.29, 3.34) |
| Australia / NZ | 5 | 27203 | 145 | 6.02 (2.26, 16.06) | 3.81 (1.18, 12.3) | 1.57 (0.91, 2.71) | 1.00 (0.70, 1.44) | 0.88 (0.60, 1.29) | 1.34 (0.90, 1.98) | 1.20 (0.63, 2.28) | 2.86 (0.89, 9.16) | - |
| East Asia | 24 | 954095 | 6961 | 2.42 (1.86, 3.15) | 1.51 (1.32, 1.74) | 1.16 (1.15, 1.17) | 1.00 (0.90, 1.11) | 1.00 (0.89, 1.12) | 1.02 (0.84, 1.24) | 1.47 (1.21, 1.79) | 3.31 (1.77, 6.19) | 1.78 (1.10, 2.88) |
| **Cancer** | | | | | | | | | | | | |
| All regions | 160 | 3839619 | 106066 | 1.11 (1.06, 1.17) | 1.01 (0.97, 1.05) | 0.96 (0.94, 0.98) | 1.00 (0.99, 1.01) | 1.05 (1.03, 1.07) | 1.13 (1.10, 1.16) | 1.28 (1.25, 1.32) | 1.55 (1.48, 1.61) | 1.90 (1.78, 2.03) |
| Europe | 73 | 1121525 | 23545 | 1.24 (1.08, 1.43) | 1.00 (0.92, 1.08) | 1.00 (0.94, 1.07) | 1.00 (0.97, 1.03) | 1.02 (0.97, 1.08) | 1.12 (1.08, 1.16) | 1.22 (1.15, 1.29) | 1.54 (1.43, 1.64) | 1.97 (1.76, 2.21) |
| North America | 38 | 1413407 | 54336 | 1.23 (1.08, 1.41) | 1.02 (0.95, 1.09) | 0.97 (0.93, 1.01) | 1.00 (0.98, 1.02) | 1.04 (1.01, 1.07) | 1.13 (1.09, 1.18) | 1.32 (1.26, 1.38) | 1.59 (1.47, 1.73) | 1.79 (1.63, 1.95) |
| Australia / NZ | 10 | 147994 | 1292 | 1.33 (0.14, 12.71) | 1.26 (0.88, 1.80) | 0.97 (0.79, 1.18) | 1.00 (0.89, 1.13) | 1.07 (0.96, 1.19) | 1.11 (0.91, 1.35) | 1.34 (1.13, 1.59) | 1.49 (1.11, 2.00) | 3.92 (0.35, 43.7) |
| East Asia | 37 | 986649 | 26567 | 1.06 (0.97, 1.16) | 0.95 (0.89, 1.01) | 0.92 (0.89, 0.94) | 1.00 (0.97, 1.03) | 1.09 (1.03, 1.15) | 1.18 (1.09, 1.28) | 1.41 (1.31, 1.51) | 1.54 (1.17, 2.01) | 1.06 (0.65, 1.74) |

Analyses excluded the first 5 years of follow-up, restricted to never-smokers without pre-existing chronic disease.

Reference category is BMI 22.5 to <25 kg/m².

CIs were calculated using floating variance estimates (reflecting independent variability within each group, including the reference group).

Results for South Asia were not shown because there were too few events to estimate HRs reliably.

Results for respiratory disease for the highest BMI category in Australia / NZ were not shown because there were too few events to estimate HRs reliably.

## eTable 16: Six WHO BMI groups vs cause-specific mortality, by geographic region

| | No of studies | No of participants | No of deaths | Underweight (15 to <18.5) | Normal weight (18.5 to <25) | Overweight (25 to <30) | Obesity Grade I (30 to <35) | Obesity Grade II (35 to <40) | Obesity Grade III (40 to <60) |
|---|---|---|---|---|---|---|---|---|---|
| **Cardiovascular disease** | | | | | | | | | |
| All geographic regions | 164 | 3276962 | 127586 | 1.38 (1.29, 1.48) | 1.00 (0.97, 1.03) | 1.22 (1.20, 1.24) | 1.77 (1.71, 1.84) | 2.61 (2.46, 2.78) | 3.72 (3.38, 4.10) |
| Europe | 79 | 1117915 | 16967 | 1.79 (1.54, 2.09) | 1.00 (0.94, 1.06) | 1.26 (1.24, 1.29) | 1.93 (1.81, 2.06) | 3.08 (2.77, 3.42) | 5.31 (4.69, 6.02) |
| North America | 37 | 1345640 | 93086 | 1.28 (1.18, 1.39) | 1.00 (0.96, 1.04) | 1.21 (1.20, 1.22) | 1.66 (1.57, 1.75) | 2.45 (2.21, 2.73) | 3.25 (2.88, 3.66) |
| Australia / NZ | 10 | 147994 | 1266 | 1.07 (0.58, 2.00) | 1.00 (0.90, 1.12) | 0.99 (0.92, 1.07) | 1.30 (1.05, 1.63) | 2.38 (1.70, 3.32) | 2.90 (1.57, 5.33) |
| East Asia | 35 | 488632 | 14871 | 1.16 (1.05, 1.28) | 1.00 (0.99, 1.01) | 1.18 (1.10, 1.27) | 1.92 (1.60, 2.30) | 2.40 (1.85, 3.13) | 1.08 (0.76, 1.54) |
| **Coronary heart disease** | | | | | | | | | |
| All geographic regions | 124 | 3599426 | 54872 | 1.17 (1.09, 1.24) | 1.00 (0.96, 1.04) | 1.34 (1.32, 1.35) | 2.02 (1.91, 2.13) | 2.81 (2.63, 3.01) | 3.81 (3.47, 4.17) |
| Europe | 57 | 1016359 | 7630 | 1.75 (1.34, 2.28) | 1.00 (0.93, 1.07) | 1.33 (1.29, 1.37) | 2.12 (1.94, 2.33) | 2.94 (2.62, 3.30) | 5.15 (4.33, 6.12) |
| North America | 33 | 1328311 | 40213 | 1.21 (1.06, 1.38) | 1.00 (0.95, 1.06) | 1.35 (1.34, 1.35) | 1.96 (1.80, 2.12) | 2.68 (2.37, 3.02) | 3.46 (3.03, 3.94) |
| Australia / NZ | 9 | 143673 | 648 | 1.64 (0.48, 5.64) | 1.00 (0.79, 1.27) | 1.01 (0.96, 1.06) | 1.30 (0.97, 1.73) | 2.88 (1.94, 4.28) | 2.43 (0.68, 8.70) |
| East Asia | 23 | 941039 | 5630 | 1.08 (0.96, 1.21) | 1.00 (0.95, 1.05) | 1.31 (1.24, 1.39) | 2.49 (1.87, 3.30) | 3.01 (1.89, 4.81) | 1.84 (0.87, 3.87) |
| **Stroke** | | | | | | | | | |
| All geographic regions | 114 | 3580423 | 40084 | 1.37 (1.23, 1.53) | 1.00 (0.96, 1.04) | 1.11 (1.09, 1.14) | 1.46 (1.39, 1.54) | 2.11 (1.93, 2.30) | 2.33 (2.05, 2.65) |
| Europe | 44 | 980210 | 4481 | 2.43 (1.84, 3.22) | 1.00 (0.91, 1.10) | 1.08 (1.06, 1.10) | 1.59 (1.41, 1.79) | 3.25 (2.43, 4.34) | 4.02 (3.15, 5.13) |
| North America | 30 | 1320458 | 20897 | 1.34 (1.17, 1.53) | 1.00 (0.96, 1.04) | 1.03 (0.99, 1.07) | 1.23 (1.13, 1.34) | 1.72 (1.52, 1.95) | 1.98 (1.69, 2.31) |
| Australia / NZ | 7 | 137292 | 308 | 3.59 (1.22, 10.6) | 1.00 (0.66, 1.51) | 0.97 (0.91, 1.03) | 1.29 (0.88, 1.89) | 1.97 (0.97, 3.97) | 3.58 (0.84, 15.2) |
| East Asia | 31 | 972419 | 13962 | 1.04 (0.90, 1.22) | 1.00 (0.95, 1.05) | 1.24 (1.17, 1.31) | 1.72 (1.57, 1.88) | 2.51 (1.86, 3.37) | 1.37 (0.85, 2.21) |
| **Respiratory disease** | | | | | | | | | |
| All geographic regions | 89 | 3353331 | 21634 | 2.73 (2.31, 3.23) | 1.00 (0.99, 1.01) | 0.90 (0.87, 0.94) | 1.16 (1.08, 1.24) | 1.79 (1.60, 1.99) | 2.85 (2.43, 3.34) |
| Europe | 31 | 923340 | 2238 | 4.70 (3.53, 6.26) | 1.00 (0.95, 1.06) | 0.87 (0.79, 0.96) | 1.26 (1.08, 1.48) | 2.21 (1.70, 2.88) | 3.85 (2.86, 5.17) |
| North America | 27 | 1278649 | 11912 | 2.41 (1.86, 3.12) | 1.00 (0.93, 1.07) | 0.89 (0.88, 0.91) | 0.98 (0.90, 1.07) | 1.46 (1.29, 1.66) | 2.58 (2.12, 3.15) |
| Australia / NZ | 5 | 27203 | 145 | 5.16 (1.94, 13.7) | 1.00 (0.76, 1.31) | 0.89 (0.69, 1.16) | 1.03 (0.56, 1.90) | 2.40 (0.74, 7.71) | - |
| East Asia | 24 | 954095 | 6961 | 2.12 (1.59, 2.84) | 1.00 (0.98, 1.02) | 0.87 (0.79, 0.96) | 1.31 (1.09, 1.56) | 2.96 (1.59, 5.50) | 1.61 (1.00, 2.60) |
| **Cancer** | | | | | | | | | |
| All geographic regions | 160 | 3839619 | 106066 | 1.10 (1.05, 1.16) | 1.00 (0.98, 1.02) | 1.10 (1.09, 1.12) | 1.31 (1.28, 1.34) | 1.57 (1.50, 1.63) | 1.96 (1.83, 2.09) |
| Europe | 73 | 1121525 | 23545 | 1.26 (1.09, 1.46) | 1.00 (0.96, 1.04) | 1.07 (1.04, 1.10) | 1.24 (1.19, 1.29) | 1.55 (1.44, 1.67) | 1.99 (1.77, 2.23) |
| North America | 38 | 1413407 | 54336 | 1.23 (1.07, 1.41) | 1.00 (0.97, 1.03) | 1.09 (1.06, 1.12) | 1.33 (1.27, 1.38) | 1.64 (1.51, 1.77) | 1.85 (1.70, 2.01) |
| Australia / NZ | 10 | 147994 | 1292 | 1.48 (0.40, 5.44) | 1.00 (0.90, 1.11) | 1.09 (0.98, 1.22) | 1.37 (1.15, 1.63) | 1.50 (1.12, 2.03) | 2.73 (0.74, 10.2) |
| East Asia | 37 | 986649 | 26567 | 1.11 (1.02, 1.20) | 1.00 (0.97, 1.03) | 1.15 (1.09, 1.21) | 1.47 (1.37, 1.57) | 1.60 (1.22, 2.10) | 1.13 (0.69, 1.85) |

Analyses excluded the first 5 years of follow-up, and were restricted to never-smokers without pre-existing chronic disease.

Reference category is WHO-defined normal weight (BMI 18.5 to <25 kg/m$^2$).

Group-specific CIs were calculated using floating variance estimates (reflecting independent variability within each group, including the reference group).

Results for South Asia are not shown because there were too few events to estimate HRs reliably.

Results for respiratory disease in the highest BMI category in Australia / NZ are not shown as there were too few events to estimate HRs reliably.

## eTable 17: Nine BMI groups vs all-cause mortality, (a) using fixed effect methods or (b) restricting follow-up to 5-15 years

| | No of studies | No of participants | No of deaths | Body-mass index (kg/m$^2$) | | | | | | | | |
|---|---|---|---|---|---|---|---|---|---|---|---|---|
| | | | | 15 to <18.5 | 18.5 to <20 | 20 to <22.5 | 22.5 to <25 | 25 to <27.5 | 27.5 to <30 | 30 to <35 | 35 to <40 | 40 to <60 |
| **(a) Using fixed effect meta-analyses** | | | | | | | | | | | | |
| All geographic regions | 189 | 3,951,455 | 385,879 | 1.36 (1.34, 1.39) | 1.07 (1.05, 1.08) | 0.98 (0.98, 0.99) | 1.00 (0.99, 1.01) | 1.08 (1.07, 1.09) | 1.24 (1.22, 1.25) | 1.45 (1.44, 1.47) | 1.91 (1.87, 1.95) | 2.66 (2.57, 2.74) |
| Europe | 89 | 1,135,600 | 56,477 | 1.70 (1.58, 1.84) | 1.16 (1.10, 1.22) | 1.04 (1.02, 1.06) | 1.00 (0.98, 1.02) | 1.06 (1.04, 1.08) | 1.19 (1.17, 1.22) | 1.43 (1.40, 1.46) | 1.87 (1.80, 1.95) | 2.88 (2.71, 3.07) |
| North America | 40 | 1,415,087 | 219,922 | 1.29 (1.25, 1.33) | 1.04 (1.02, 1.06) | 0.97 (0.96, 0.98) | 1.00 (0.99, 1.01) | 1.09 (1.08, 1.10) | 1.25 (1.24, 1.27) | 1.46 (1.45, 1.48) | 1.94 (1.89, 1.98) | 2.66 (2.56, 2.76) |
| Australia / NZ | 11 | 149,602 | 4,656 | 1.45 (1.12, 1.86) | 1.30 (1.10, 1.54) | 1.03 (0.95, 1.13) | 1.00 (0.94, 1.06) | 0.99 (0.93, 1.05) | 1.11 (1.04, 1.19) | 1.30 (1.21, 1.40) | 1.79 (1.56, 2.06) | 2.15 (1.65, 2.79) |
| East Asia | 46 | 1,074,385 | 100,784 | 1.37 (1.34, 1.41) | 1.08 (1.05, 1.10) | 0.99 (0.97, 1.00) | 1.00 (0.99, 1.01) | 1.07 (1.06, 1.09) | 1.24 (1.21, 1.27) | 1.49 (1.43, 1.55) | 1.78 (1.56, 2.03) | 1.37 (1.13, 1.66) |
| South Asia | 3 | 176,781 | 4,040 | 1.30 (1.22, 1.38) | 1.06 (0.98, 1.16) | 1.02 (0.96, 1.08) | 1.00 (0.93, 1.07) | 0.98 (0.89, 1.08) | 0.90 (0.78, 1.04) | 1.00 (0.83, 1.21) | 1.45 (0.95, 2.20) | 1.60 (0.66, 3.86) |
| **(b) Restricting follow-up to 5-15 years** | | | | | | | | | | | | |
| All geographic regions | 187 | 3,949,650 | 231,712 | 1.53 (1.45, 1.62) | 1.15 (1.11, 1.19) | 1.01 (0.99, 1.03) | 1.00 (0.99, 1.01) | 1.05 (1.04, 1.06) | 1.19 (1.17, 1.21) | 1.43 (1.39, 1.46) | 1.90 (1.82, 1.98) | 2.64 (2.50, 2.80) |
| Europe | 88 | 1,134,616 | 45,383 | 1.98 (1.79, 2.19) | 1.32 (1.18, 1.48) | 1.03 (0.97, 1.09) | 1.00 (0.97, 1.03) | 1.06 (1.04, 1.08) | 1.18 (1.15, 1.21) | 1.46 (1.40, 1.53) | 1.94 (1.82, 2.06) | 3.02 (2.78, 3.27) |
| North America | 39 | 1,414,292 | 103,616 | 1.53 (1.42, 1.64) | 1.12 (1.06, 1.17) | 1.01 (0.99, 1.04) | 1.00 (0.99, 1.01) | 1.05 (1.02, 1.07) | 1.18 (1.13, 1.23) | 1.40 (1.34, 1.47) | 1.88 (1.75, 2.03) | 2.55 (2.31, 2.82) |
| Australia / NZ | 11 | 149,576 | 4,004 | 1.48 (1.13, 1.94) | 1.41 (1.13, 1.76) | 1.04 (0.93, 1.17) | 1.00 (0.94, 1.07) | 1.00 (0.93, 1.08) | 1.13 (0.99, 1.28) | 1.41 (1.28, 1.56) | 1.88 (1.60, 2.21) | 2.30 (1.75, 3.01) |
| East Asia | 46 | 1,074,385 | 74,669 | 1.37 (1.24, 1.52) | 1.09 (1.04, 1.15) | 0.99 (0.97, 1.02) | 1.00 (0.97, 1.03) | 1.06 (1.03, 1.09) | 1.26 (1.21, 1.32) | 1.49 (1.40, 1.60) | 1.89 (1.63, 2.19) | 1.36 (1.02, 1.80) |
| South Asia | 3 | 176,781 | 4,040 | 1.30 (1.10, 1.52) | 1.08 (0.90, 1.29) | 1.04 (0.96, 1.13) | 1.00 (0.89, 1.13) | 0.99 (0.83, 1.19) | 0.94 (0.72, 1.24) | 1.03 (0.85, 1.26) | 1.44 (0.94, 2.20) | 1.62 (0.67, 3.91) |

Analyses excluded the first 5 years of follow-up, and were restricted to never-smokers without pre-existing chronic disease.

Reference categories are BMI 22.5 to <25 kg/m$^2$.

Group-specific CIs were calculated using floating variance estimates (reflecting independent variability within each group, including the reference group).

**eTable 18: Six WHO BMI groups vs all-cause mortality, (a) using fixed effect methods or (b) restricting follow-up to 5-15 years**

| | No of studies | No of participants | No of deaths | Underweight (15 to <18.5) | Normal weight (18.5 to <25) | Overweight (25 to <30) | Obesity Grade I (30 to <35) | Obesity Grade II (35 to <40) | Obesity Grade III (40 to <60) |
|---|---|---|---|---|---|---|---|---|---|
| **(a) Using fixed effect meta-analyses** | | | | | | | | | |
| All geographic regions | 189 | 3,951,455 | 385,879 | 1.36 (1.34, 1.39) | 1.00 (1.00, 1.00) | 1.13 (1.13, 1.14) | 1.45 (1.44, 1.47) | 1.91 (1.87, 1.95) | 2.66 (2.57, 2.74) |
| Europe | 89 | 1,135,600 | 56,477 | 1.67 (1.55, 1.80) | 1.00 (0.99, 1.01) | 1.09 (1.07, 1.10) | 1.40 (1.37, 1.43) | 1.84 (1.76, 1.91) | 2.83 (2.65, 3.01) |
| North America | 40 | 1,415,087 | 219,922 | 1.30 (1.26, 1.34) | 1.00 (0.99, 1.01) | 1.16 (1.15, 1.16) | 1.48 (1.46, 1.50) | 1.95 (1.90, 2.00) | 2.68 (2.58, 2.79) |
| Australia / NZ | 11 | 149,602 | 4,656 | 1.41 (1.10, 1.82) | 1.00 (0.95, 1.05) | 1.01 (0.97, 1.06) | 1.27 (1.18, 1.37) | 1.75 (1.52, 2.01) | 2.09 (1.61, 2.72) |
| East Asia | 46 | 1,074,385 | 100,784 | 1.37 (1.33, 1.40) | 1.00 (0.99, 1.01) | 1.11 (1.10, 1.13) | 1.48 (1.43, 1.54) | 1.77 (1.56, 2.02) | 1.36 (1.12, 1.65) |
| South Asia | 3 | 176,781 | 4,040 | 1.27 (1.19, 1.35) | 1.00 (0.96, 1.04) | 0.93 (0.86, 1.01) | 0.98 (0.82, 1.18) | 1.42 (0.93, 2.15) | 1.57 (0.65, 3.78) |
| **(b) Restricting follow-up to 5-15 years** | | | | | | | | | |
| All geographic regions | 187 | 3,949,650 | 231,712 | 1.49 (1.41, 1.58) | 1.00 (0.98, 1.02) | 1.08 (1.07, 1.09) | 1.42 (1.38, 1.45) | 1.87 (1.80, 1.95) | 2.60 (2.45, 2.75) |
| Europe | 88 | 1,134,616 | 45,383 | 1.82 (1.68, 1.99) | 1.00 (0.97, 1.04) | 1.10 (1.10, 1.11) | 1.46 (1.40, 1.53) | 1.92 (1.79, 2.06) | 2.95 (2.72, 3.19) |
| North America | 39 | 1,414,292 | 103,616 | 1.50 (1.39, 1.62) | 1.00 (0.97, 1.03) | 1.08 (1.06, 1.10) | 1.39 (1.34, 1.44) | 1.87 (1.74, 2.01) | 2.53 (2.30, 2.78) |
| Australia / NZ | 11 | 149,576 | 4,004 | 1.42 (1.09, 1.86) | 1.00 (0.96, 1.05) | 1.01 (0.95, 1.09) | 1.35 (1.23, 1.48) | 1.79 (1.54, 2.08) | 2.24 (1.71, 2.93) |
| East Asia | 46 | 1,074,385 | 74,669 | 1.36 (1.22, 1.52) | 1.00 (0.96, 1.04) | 1.11 (1.10, 1.12) | 1.50 (1.41, 1.58) | 1.86 (1.60, 2.16) | 1.35 (1.02, 1.79) |
| South Asia | 3 | 176,781 | 4,040 | 1.28 (1.11, 1.46) | 1.00 (0.99, 1.01) | 0.93 (0.80, 1.09) | 0.99 (0.81, 1.19) | 1.42 (0.93, 2.16) | 1.57 (0.65, 3.79) |

Analyses excluded the first 5 years of follow-up, and were restricted to never-smokers without pre-existing chronic disease.

Reference category is WHO-defined normal weight (BMI 18.5 to <25 kg/$m^2$).

Group-specific CIs were calculated using floating variance estimates (reflecting independent variability within each group, including the reference group).

**eTable 19: Effects of adjustment for race, or of exclusion of diabetes at baseline, in studies that could do this**

| | No of studies | No of participants | No of deaths | Underweight (15 to <18.5) | Normal weight (18.5 to <25) | Overweight (25 to <30) | Obesity Grade I (30 to <35) | Obesity Grade II (35 to <40) | Obesity Grade III (40 to <60) |
|---|---|---|---|---|---|---|---|---|---|
| **Adjustment for race** | | | | | | | | | |
| Not adjusted for race | 68 | 205478 | 23087 | 1.69 (1.50, 1.90) | 1.00 (0.95, 1.05) | 1.05 (1.05, 1.06) | 1.34 (1.26, 1.43) | 1.82 (1.66, 2.00) | 2.64 (2.32, 3.00) |
| Adjusted for race | 68 | 205478 | 23087 | 1.69 (1.50, 1.90) | 1.00 (0.95, 1.05) | 1.05 (1.04, 1.06) | 1.33 (1.25, 1.41) | 1.80 (1.64, 1.97) | 2.60 (2.28, 2.95) |
| P value for heterogeneity | | | | 0.988 | - | 0.963 | 0.894 | 0.868 | 0.876 |
| | | | | | | | | | |
| **Exclusion of baseline diabetes** | | | | | | | | | |
| Including diabetes | 100 | 441577 | 45509 | 1.53 (1.39, 1.68) | 1.00 (0.96, 1.04) | 1.07 (1.06, 1.07) | 1.39 (1.33, 1.45) | 1.85 (1.73, 1.99) | 2.68 (2.42, 2.97) |
| Excluding diabetes | 100 | 429264 | 42693 | 1.55 (1.40, 1.71) | 1.00 (0.96, 1.04) | 1.07 (1.06, 1.07) | 1.36 (1.30, 1.42) | 1.78 (1.67, 1.90) | 2.66 (2.42, 2.92) |
| P value for heterogeneity | | | | 0.893 | - | 0.980 | 0.765 | 0.800 | 0.765 |

Analyses excluded the first 5 years of follow-up, and were restricted to never-smokers without pre-existing chronic disease.

Reference category is WHO-defined normal weight (BMI 18.5 to <25 $kg/m^2$).

Group-specific CIs were calculated using floating variance estimates (reflecting independent variability within each group, including the reference group).

**eTable 20: Six WHO BMI groups vs all-cause mortality, in studies with full baseline data on heart disease, stroke & cancer**

| | Underweight (BMI 15 to <18.5) | Normal weight (18.5 to <25) | Overweight (25 to <30) | Obesity Grade I (30 to <35) | Obesity Grade II (35 to <40) | Obesity Grade III (40 to <60) |
|---|---|---|---|---|---|---|
| **19 studies; 820,301 participants; 40,205 deaths** | | | | | | |
| No. of participants /deaths | 13589 / 1221 | 425611 / 19522 | 267626 / 12880 | 82213 / 4427 | 23036 / 1464 | 8226 / 691 |
| HR (95% CI) | 1.56 (1.37, 1.78) | 1.00 (0.95, 1.06) | 1.09 (1.04, 1.13) | 1.40 (1.31, 1.50) | 1.75 (1.60, 1.91) | 2.51 (2.09, 3.00) |

Analyses excluded the first 5 years of follow-up, and were restricted to never-smokers without pre-existing chronic disease.

Reference category is WHO-defined normal weight (BMI 18.5 to <25 $kg/m^2$).

Group-specific CIs were calculated using floating variance estimates (reflecting independent variability within each group, including the reference group).

## eTable 21: Six WHO BMI groups vs cause-specific mortality, by year of baseline survey

| | No of studies | No of participants | No of deaths | Underweight (15 to <18.5) | Normal weight (18.5 to <25) | Overweight (25 to <30) | Obesity Grade I (30 to <35) | Obesity Grade II (35 to <40) | Obesity Grade III (40 to <60) |
|---|---|---|---|---|---|---|---|---|---|
| **All studies** | | | | | | | | | |
| BMI recorded <1990 | 113 | 1,054,589 | 115,745 | 1.43 (1.34, 1.53) | 1.00 (0.98, 1.02) | 1.14 (1.14, 1.15) | 1.58 (1.52, 1.64) | 2.10 (1.98, 2.23) | 2.88 (2.57, 3.22) |
| BMI recorded ≥1990 | 76 | 2,896,866 | 270,134 | 1.53 (1.40, 1.67) | 1.00 (0.97, 1.03) | 1.05 (1.03, 1.07) | 1.31 (1.29, 1.33) | 1.76 (1.67, 1.84) | 2.49 (2.35, 2.64) |
| P-value for heterogeneity | | | | 0.27 | | <0.001 | <0.001 | <0.001 | 0.070 |
| **European studies** | | | | | | | | | |
| BMI recorded <1990 | 58 | 335,001 | 23,812 | 1.39 (1.22, 1.58) | 1.00 (0.95, 1.05) | 1.13 (1.12, 1.15) | 1.63 (1.53, 1.74) | 2.09 (1.90, 2.30) | 3.00 (2.63, 3.43) |
| BMI recorded ≥1990 | 31 | 800,599 | 32,665 | 1.88 (1.70, 2.07) | 1.00 (0.94, 1.06) | 1.08 (1.08, 1.08) | 1.33 (1.27, 1.40) | 1.88 (1.73, 2.05) | 2.88 (2.59, 3.21) |
| P-value for heterogeneity | | | | 0.001 | | 0.177 | 0.010 | 0.217 | 0.437 |
| **North American studies** | | | | | | | | | |
| BMI recorded <1990 | 25 | 547,820 | 61,764 | 1.38 (1.25, 1.52) | 1.00 (0.96, 1.04) | 1.12 (1.10, 1.14) | 1.49 (1.40, 1.58) | 2.13 (1.96, 2.32) | 3.01 (2.60, 3.48) |
| BMI recorded ≥1990 | 15 | 867,267 | 158,158 | 1.60 (1.29, 1.99) | 1.00 (0.92, 1.08) | 1.05 (1.00, 1.10) | 1.26 (1.25, 1.28) | 1.66 (1.53, 1.81) | 2.17 (1.98, 2.37) |
| P-value for heterogeneity | | | | 0.601 | | 0.045 | 0.002 | 0.001 | 0.006 |
| **Australia/New Zealand** | | | | | | | | | |
| BMI recorded <1990 | 5 | 13,825 | 1,237 | 1.37 (0.79, 2.37) | 1.00 (0.95, 1.06) | 0.98 (0.87, 1.10) | 1.41 (0.95, 2.09) | 1.57 (1.08, 2.30) | 1.00 (0.31, 3.26) |
| BMI recorded ≥1990 | 6 | 135,777 | 3,419 | 1.49 (1.11, 2.01) | 1.00 (0.93, 1.07) | 1.03 (0.95, 1.13) | 1.29 (1.19, 1.39) | 1.77 (1.40, 2.25) | 2.22 (1.68, 2.93) |
| P-value for heterogeneity | | | | 0.679 | | 0.651 | 0.922 | 0.805 | 0.178 |
| **East Asian studies** | | | | | | | | | |
| BMI recorded <1990 | 25 | 157,943 | 28,932 | 1.25 (1.12, 1.40) | 1.00 (0.99, 1.01) | 1.15 (1.11, 1.18) | 1.73 (1.48, 2.03) | 1.38 (1.06, 1.81) | 1.01 (0.80, 1.27) |
| BMI recorded >1990 | 21 | 916,442 | 71,852 | 1.47 (1.28, 1.70) | 1.00 (0.93, 1.07) | 1.09 (1.08, 1.10) | 1.41 (1.34, 1.49) | 1.90 (1.64, 2.21) | 2.65 (1.87, 3.76) |
| P-value for heterogeneity | | | | 0.157 | | 0.036 | 0.022 | 0.079 | <0.001 |

Analyses excluded the first 5 years of follow-up, and were restricted to never-smokers without pre-existing chronic disease.

Reference category is WHO-defined normal weight (BMI 18.5 to <25 kg/m$^2$).

Group-specific CIs were calculated using floating variance estimates (reflecting independent variability within each group, including the reference group).

Studies were grouped according to median year of baseline survey (see eTable 1). All studies from South Asia began recruitment after 1990.

P-values for heterogeneity were calculated using multivariate meta-regression.

## eTable 22: Six WHO BMI groups vs all-cause mortality, by method of assessing BMI

| | No of studies | No of participants | No of deaths | Underweight (15 to <18.5) | Normal weight (18.5 to <25) | Overweight (25 to <30) | Obesity Grade I (30 to <35) | Obesity Grade II (35 to <40) | Obesity Grade III (40 to <60) |
|---|---|---|---|---|---|---|---|---|---|
| **All geographic regions** | | | | | | | | | |
| Measured BMI | 153 | 1,743,551 | 129,028 | 1.52 (1.41, 1.64) | 1.00 (0.97, 1.03) | 1.09 (1.09, 1.10) | 1.44 (1.40, 1.49) | 1.92 (1.81, 2.04) | 2.79 (2.56, 3.04) |
| Self-reported BMI | 36 | 2,207,904 | 256,851 | 1.38 (1.28, 1.48) | 1.00 (0.98, 1.02) | 1.13 (1.11, 1.14) | 1.47 (1.42, 1.51) | 2.00 (1.90, 2.12) | 2.68 (2.42, 2.96) |
| p-values for heterogeneity | | | | 0.304 | | 0.054 | 0.340 | 0.370 | 0.819 |
| **Europe** | | | | | | | | | |
| Measured BMI | 85 | 588,960 | 33,025 | 1.52 (1.37, 1.69) | 1.00 (0.96, 1.04) | 1.11 (1.09, 1.12) | 1.53 (1.46, 1.60) | 1.98 (1.84, 2.13) | 3.07 (2.76, 3.41) |
| Self-reported BMI | 4 | 546,640 | 23,452 | 1.83 (1.57, 2.13) | 1.00 (0.99, 1.01) | 1.21 (1.01, 1.44) | 1.36 (1.07, 1.72) | 2.72 (1.70, 4.34) | 2.46 (2.11, 2.88) |
| p-values for heterogeneity | | | | 0.003 | | 0.525 | 0.864 | 0.547 | 0.851 |
| **North America** | | | | | | | | | |
| Measured BMI | 20 | 86,010 | 12,868 | 1.74 (1.43, 2.12) | 1.00 (0.92, 1.09) | 1.00 (0.97, 1.04) | 1.21 (1.11, 1.32) | 1.72 (1.48, 1.99) | 2.28 (1.82, 2.86) |
| Self-reported BMI | 20 | 1,329,077 | 207,054 | 1.34 (1.26, 1.43) | 1.00 (0.98, 1.02) | 1.14 (1.12, 1.16) | 1.51 (1.45, 1.57) | 2.08 (1.94, 2.24) | 2.88 (2.61, 3.17) |
| p-values for heterogeneity | | | | 0.002 | | 0.004 | <0.001 | 0.037 | 0.057 |
| **East Asia** | | | | | | | | | |
| Measured BMI | 35 | 844,914 | 75,444 | 1.44 (1.24, 1.67) | 1.00 (0.95, 1.05) | 1.14 (1.11, 1.17) | 1.55 (1.41, 1.70) | 1.78 (1.50, 2.12) | 2.62 (1.83, 3.73) |
| Self-reported BMI | 11 | 229,471 | 25,340 | 1.28 (1.14, 1.43) | 1.00 (0.99, 1.01) | 1.09 (1.02, 1.16) | 1.53 (1.34, 1.75) | 1.80 (1.25, 2.60) | 1.29 (0.88, 1.89) |
| p-values for heterogeneity | | | | 0.445 | | 0.377 | 0.643 | 0.834 | <0.001 |

Analyses excluded the first 5 years of follow-up, and were restricted to never-smokers without pre-existing chronic disease.

Reference category is participants with WHO-defined normal weight (BMI value between 18.5 and <25 $kg/m^2$).

Group-specific CIs were calculated using floating variance estimates (reflecting independent variability within each group, including the reference group).

P-values for heterogeneity were calculated using multivariate meta-regression.

Results from Australia / NZ and South Asia are not shown, as all but one study used measured BMI.

**eTable 23: Six WHO BMI groups vs all-cause mortality, by population source (occupational or population based)**

| | Underweight (BMI 15 to <18.5) | Normal weight (18.5 to <25) | Overweight (25 to <30) | Obesity Grade I (30 to <35) | Obesity Grade II (35 to <40) | Obesity Grade III (40 to <60) |
|---|---|---|---|---|---|---|
| **Occupational studies (30 studies; 397,442 participants; 24,976 deaths)** | | | | | | |
| No of participants / deaths | 8294 / 301 | 216071 / 11098 | 121152 / 9384 | 37223 / 3040 | 10345 / 786 | 4357 / 367 |
| HR (95% CI) | 1.32 (1.15, 1.53) | 1.00 (0.95, 1.05) | 1.19 (1.14, 1.24) | 1.68 (1.57, 1.80) | 2.22 (2.04, 2.41) | 3.31 (2.99, 3.67) |
| **Approximately population-based studies (159 studies, 3,554,013 participants; 360,903 deaths)** | | | | | | |
| No of participants / deaths | 105797 / 12425 | 1929479 / 181425 | 1128951 / 120909 | 293617 / 34278 | 70482 / 8393 | 25687 / 3473 |
| HR (95% CI) | 1.48 (1.40, 1.57) | 1.00 (0.98, 1.02) | 1.09 (1.09, 1.10) | 1.40 (1.37, 1.43) | 1.86 (1.78, 1.94) | 2.60 (2.43, 2.78) |
| P-value for heterogeneity | 0.458 | . | 0.002 | <0.001 | 0.007 | 0.003 |

Analyses excluded the first 5 years of follow-up, and were restricted to never-smokers without pre-existing chronic disease.

Reference category is WHO-defined normal weight (BMI 18.5 to <25 kg/$m^2$).

Group-specific CIs were calculated using floating variance estimates (reflecting independent variability within each group, including the reference group).

**eFigure 1.** Flow diagram

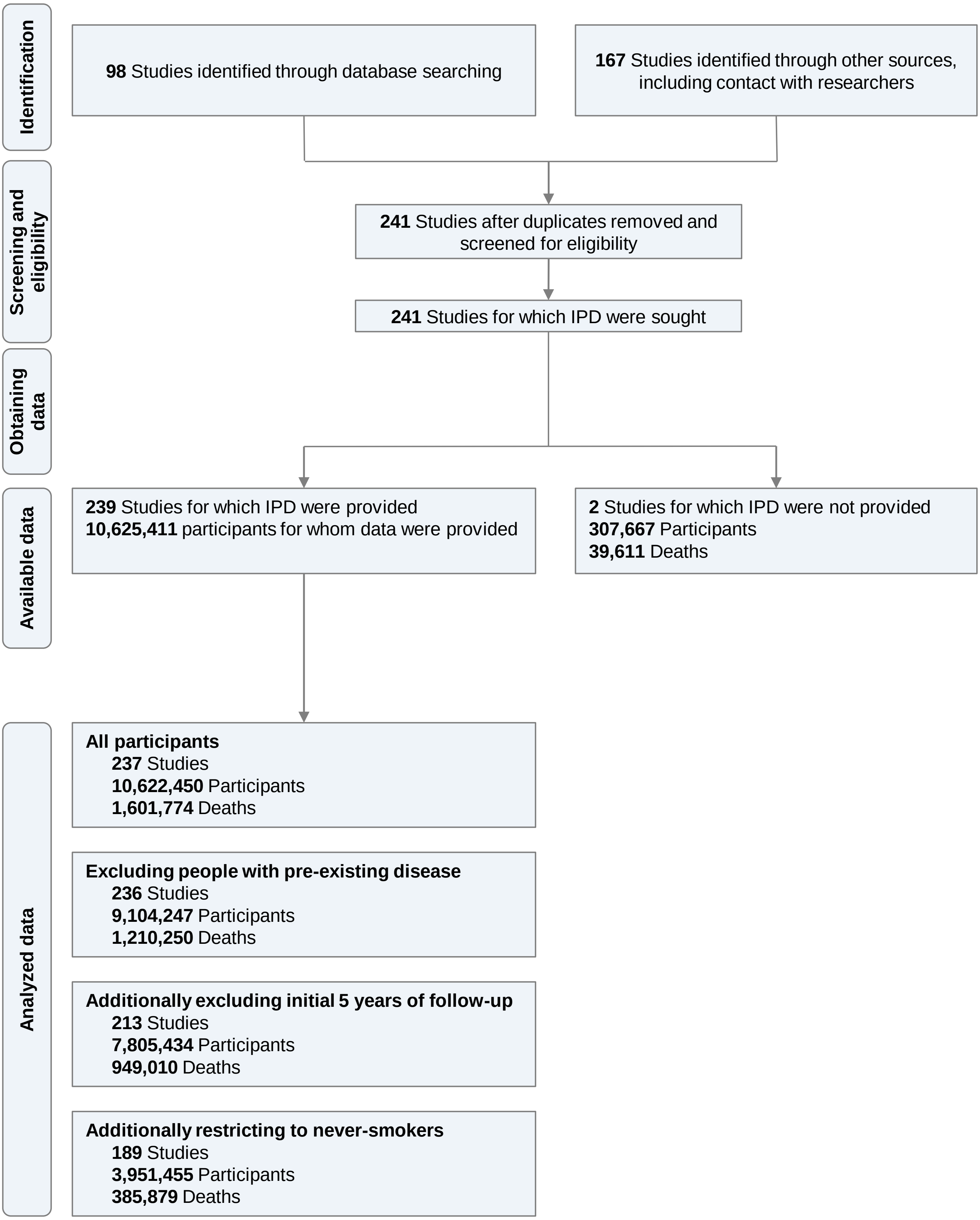

## eFigure 2: 9 BMI categories vs all-cause mortality, by baseline age & 3 geographic regions

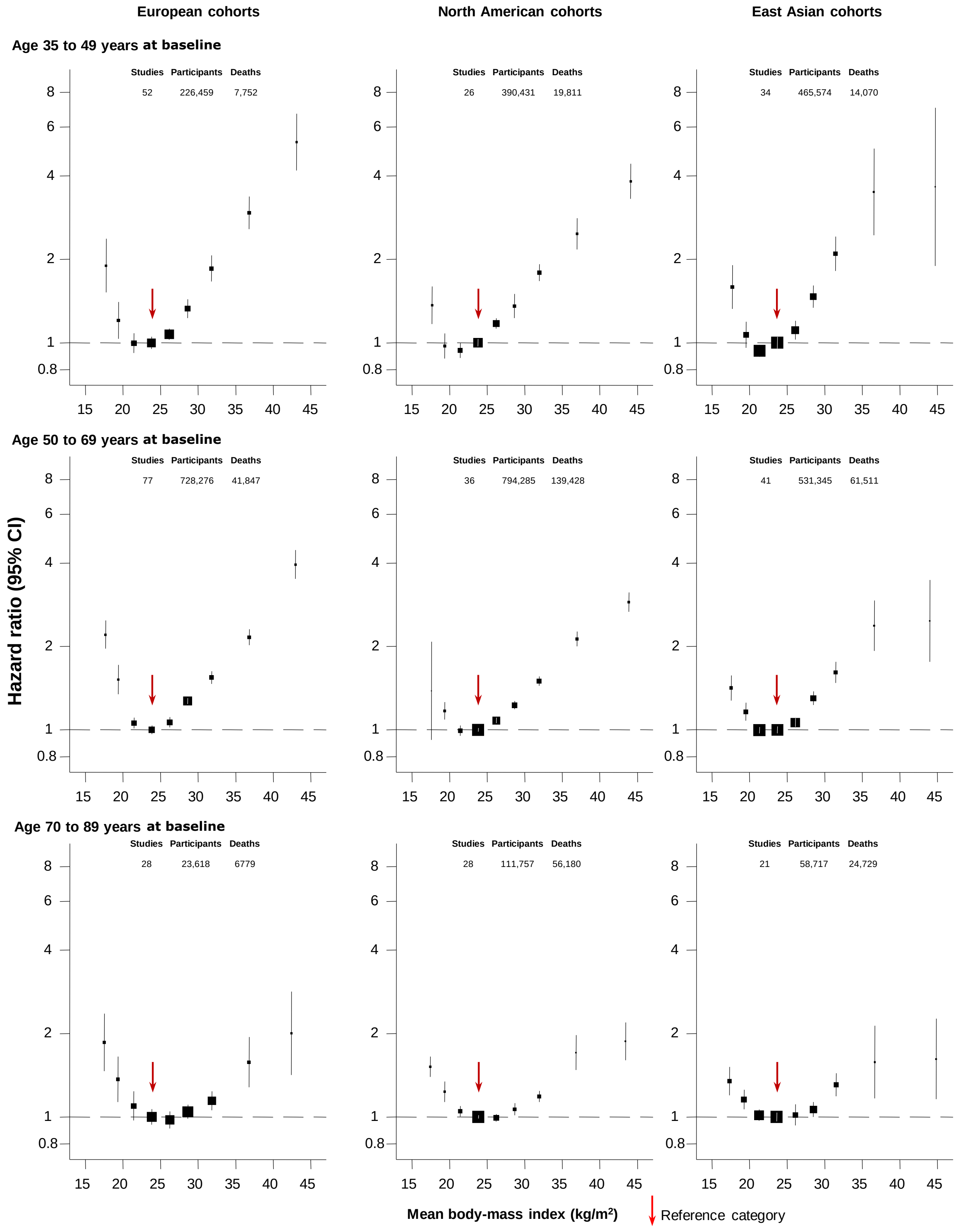


Analyses are restricted to never-smokers without pre-existing chronic disease, and exclude the first 5 years of follow-up.

BMI groups: 15 to <18.5, 18.5 to <20, 20 to <22.5, 22.5 to <25, 25 to <27.5, 27.5 to <30, 30 to <35, 35 to <40, and 40 to <60 kg/m$^2$. Reference category (arrow) is 22.5 to <25 kg/m$^2$.

Group-specific CIs are from floating variance estimates (reflecting independent variability within each category, including the reference group). Areas of squares are proportional to the information content in each.

**eFigure 3: 9 BMI categories vs all-cause mortality, by sex and 3 geographic regions**

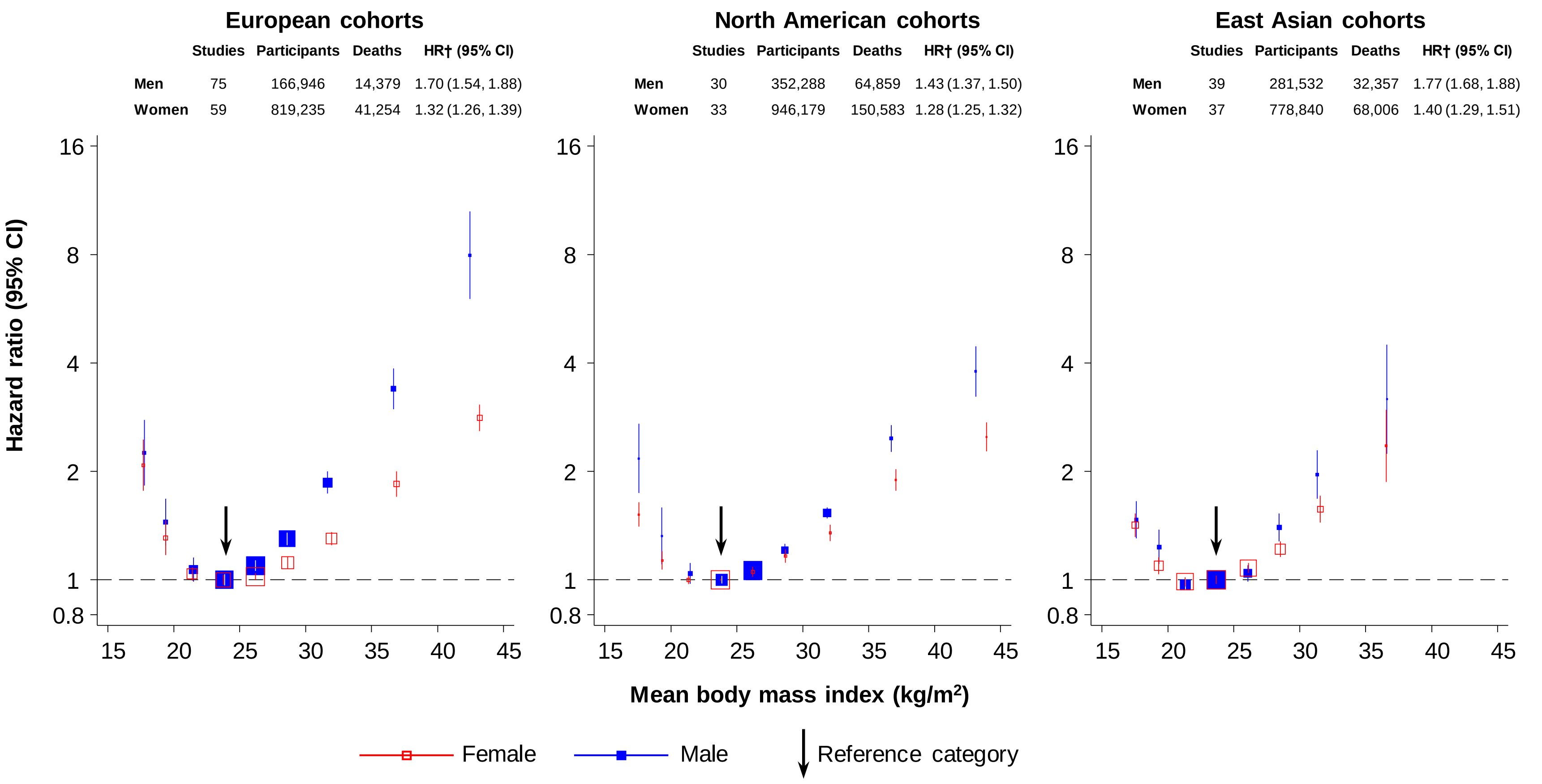


Analyses are restricted to never-smokers without pre-existing chronic disease, and exclude the first 5 years of follow-up.

BMI groups: 15 to <18.5, 18.5 to <20, 20 to <22.5, 22.5 to <25, 25 to <27.5, 27.5 to <30, 30 to <35, 35 to <40, and 40 to <60 kg/m$^2$.

Reference category (arrow) is BMI 22.5 to <25 kg/m$^2$.

Group-specific CIs are from floating variance estimates (reflecting independent variability within each category, including reference).

Areas of squares are proportional to the information content in each. The highest BMI category in East Asians was removed because of small numbers.

**eFigure 4: 9 BMI categories vs all-cause mortality, by sex and 3 geographic regions, in studies with both men and women**

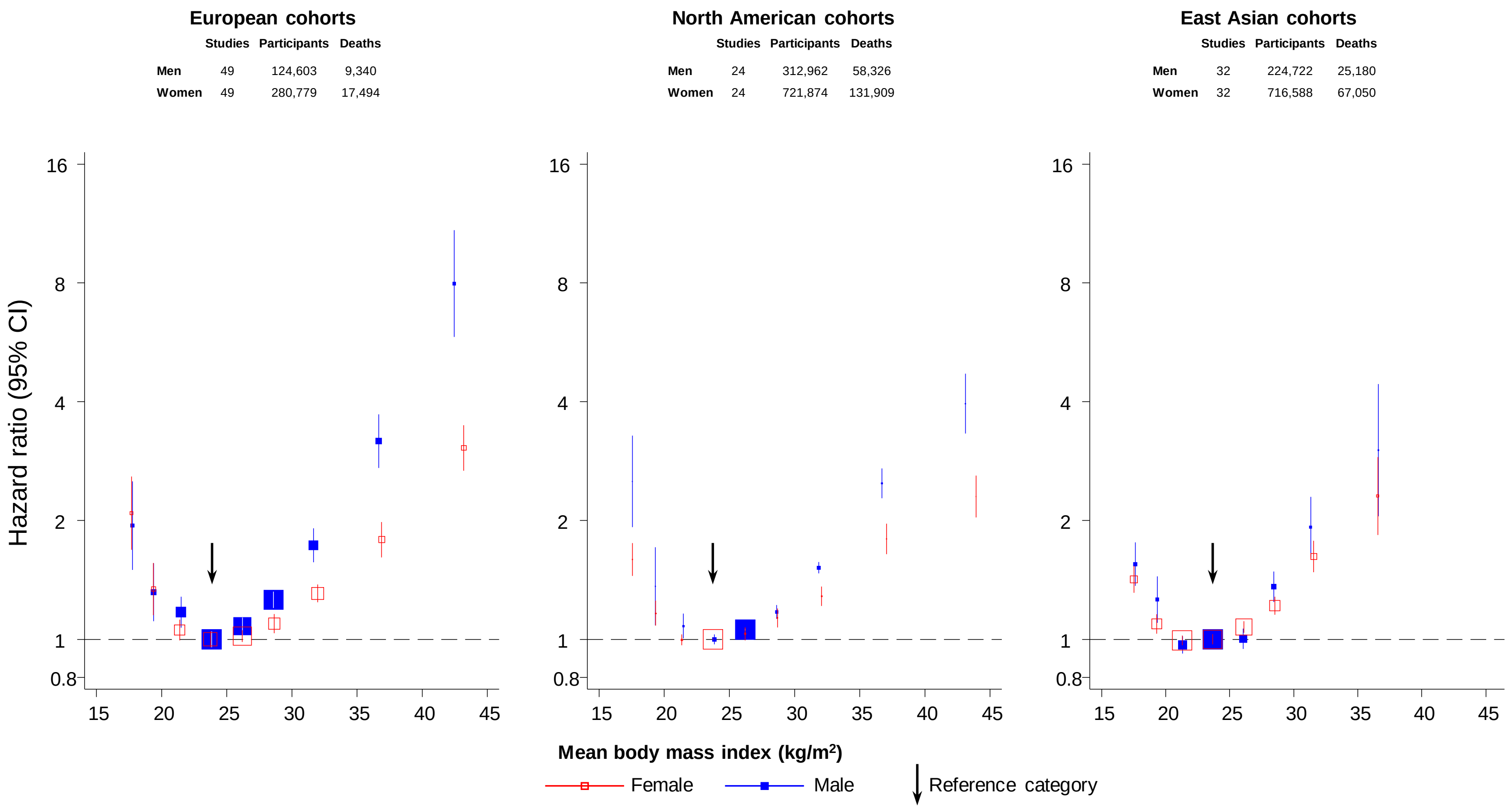


Analyses are restricted to never-smokers without pre-existing chronic disease, and exclude the first 5 years of follow-up.

BMI groups: 15 to <18.5, 18.5 to <20, 20 to <22.5, 22.5 to <25, 25 to <27.5, 27.5 to <30, 30 to <35, 35 to <40, and 40 to <60 kg/$m^2$.

Reference category (arrow) is BMI 22.5 to <25 kg/$m^2$.

Group-specific CIs are from floating variance estimates (reflecting independent variability within each category, including reference).

Areas of squares are proportional to the information content in each. The highest BMI category in East Asians was removed because of small numbers.

## eFigure 5: 9 BMI categories vs cause-specific mortality, by 3 geographic regions

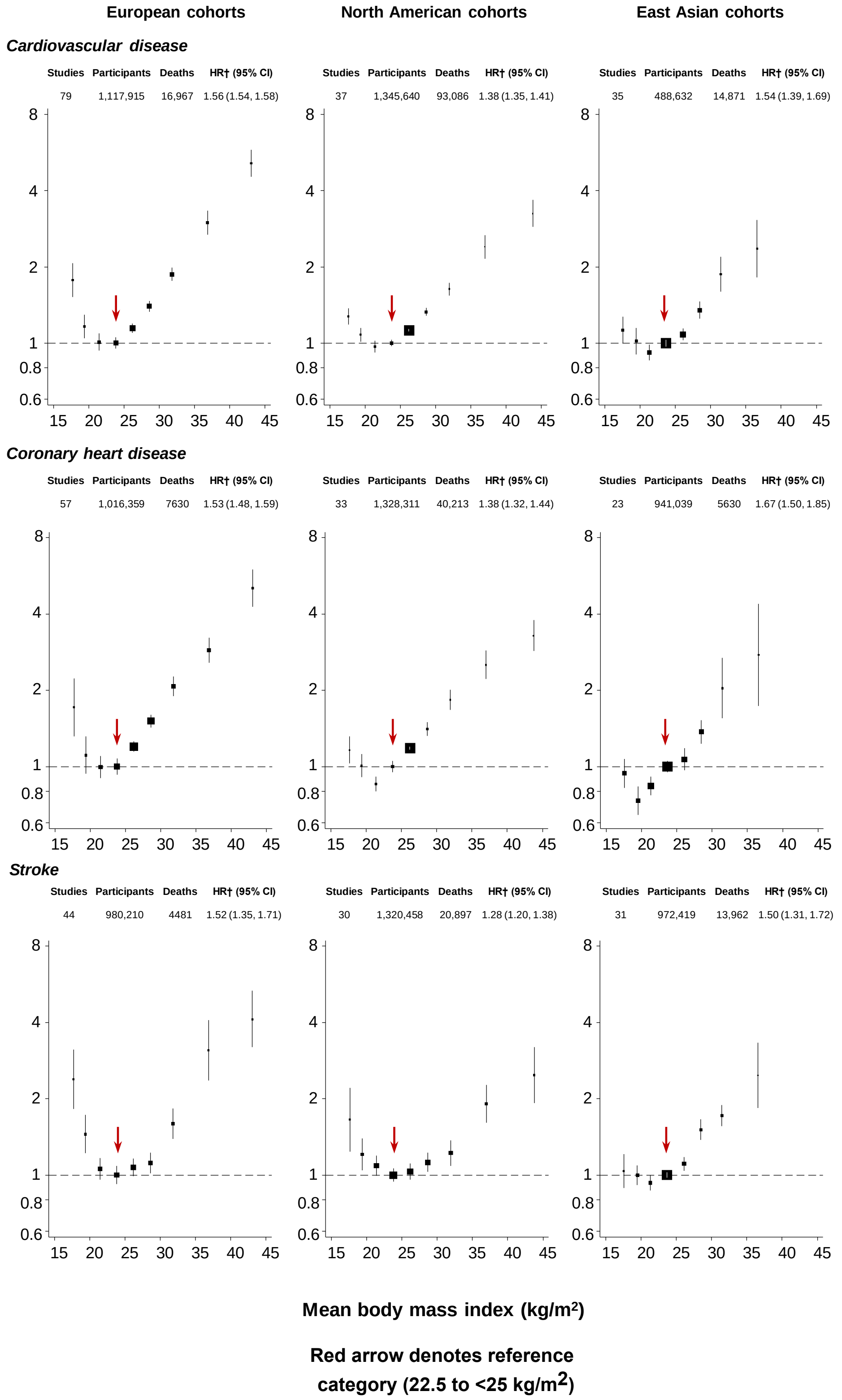

## eFigure 5 (continued)

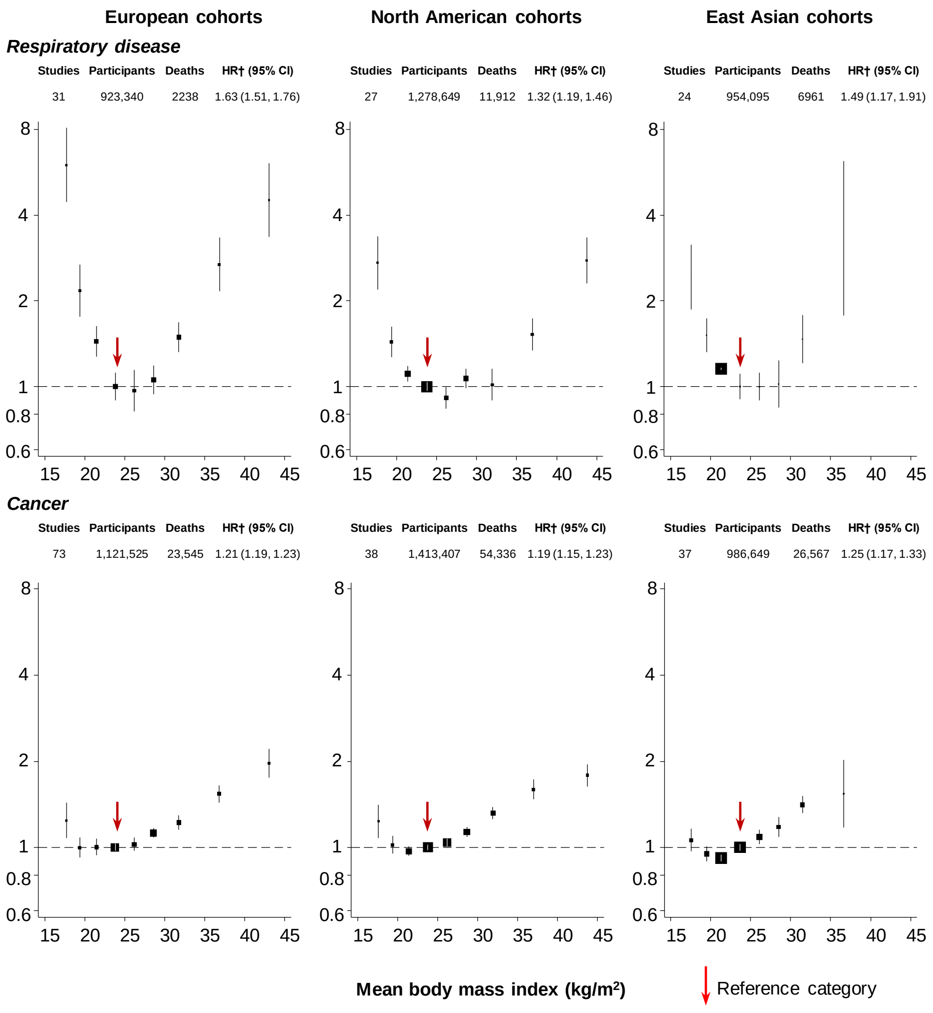


**Red arrow denotes reference category (22.5 to <25 kg/m$^2$)**

†HR, hazard ratio per 5 kg/m$^2$ BMI in the range BMI >25 kg/m$^2$ (and 95% CI).

Analyses are restricted to never-smokers without pre-existing chronic disease, exclude the first 5 years of follow-up, and include data from all geographical regions.

BMI groups: 15 to <18.5, 18.5 <20, 20 to <22.5, 22.5 to <25, 25 to <27.5, 27.5 to <30, 30 to <35, 35 to <40, and 40 to <60 kg/m$^2$. Reference category (arrow) is 22.5 to <25 kg/m$^2$. Reference group is BMI 22.5 to 25 kg/m$^2$.

CIs are from floating variance estimates (reflecting independent variability within each category, including reference).

Areas of squares are proportional to the information content of each.

The highest BMI category in East Asians was removed because it included only a limited number of individuals.

**eFigure 6: 9 BMI categories vs all-cause mortality; effects of adjustments and exclusions**

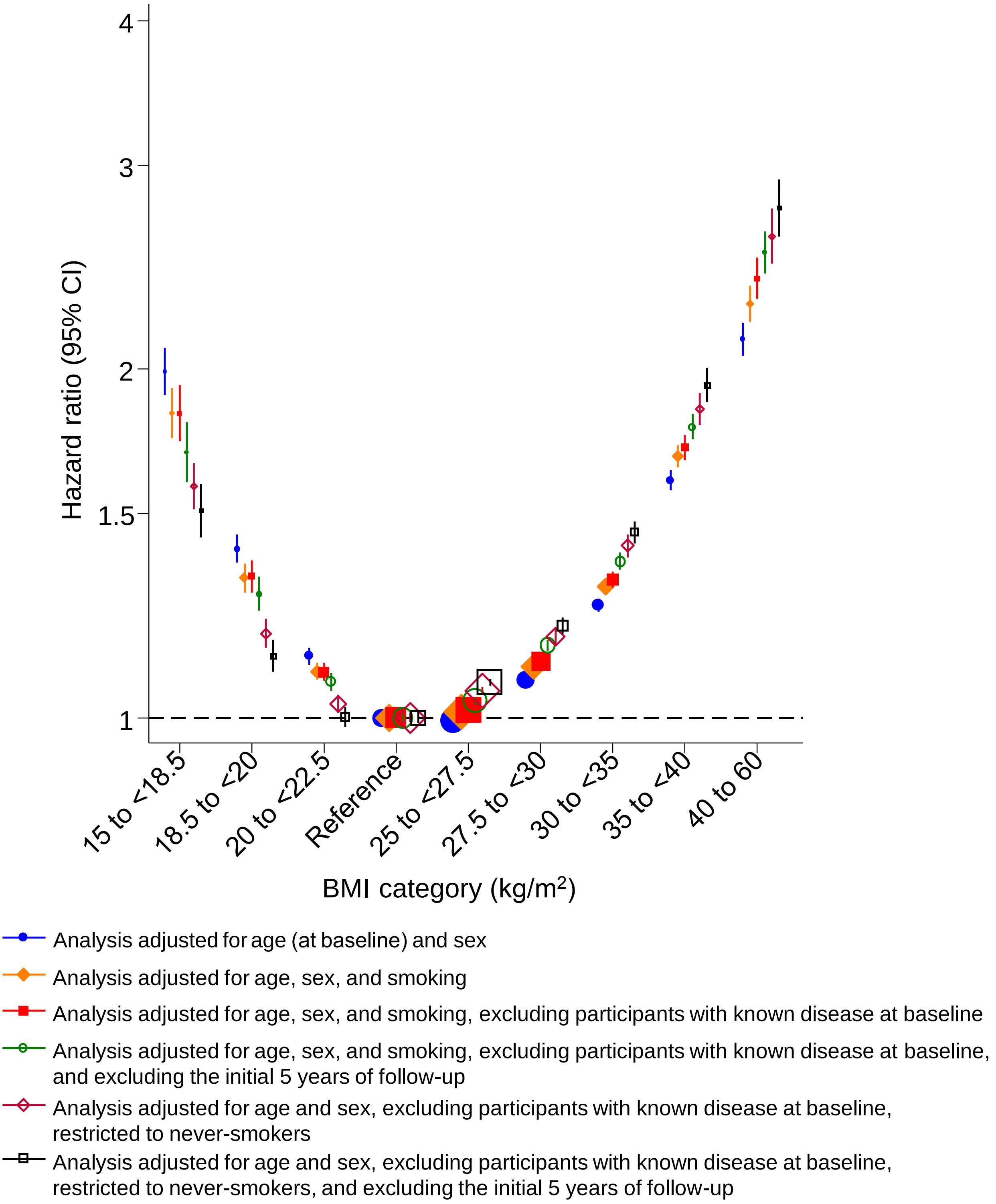


BMI groups: 15 to <18.5, 18.5 to <20, 20 to <22.5, 22.5 to <25, 25 to <27.5, 27.5 to <30, 30 to<35, 35 to<40 and 40 to <60 kg/m$^2$

Reference category is BMI 22.5 to <25 kg/m$^2$ for all analyses.

Group-specific CIs were calculated using floating variance estimates (reflecting independent variability within each group, including the reference group).

Areas of symbols are proportional to the information content of each.

**eFigure 7: 9 BMI categories vs all-cause mortality in the studies that could provide analyses by age at risk**

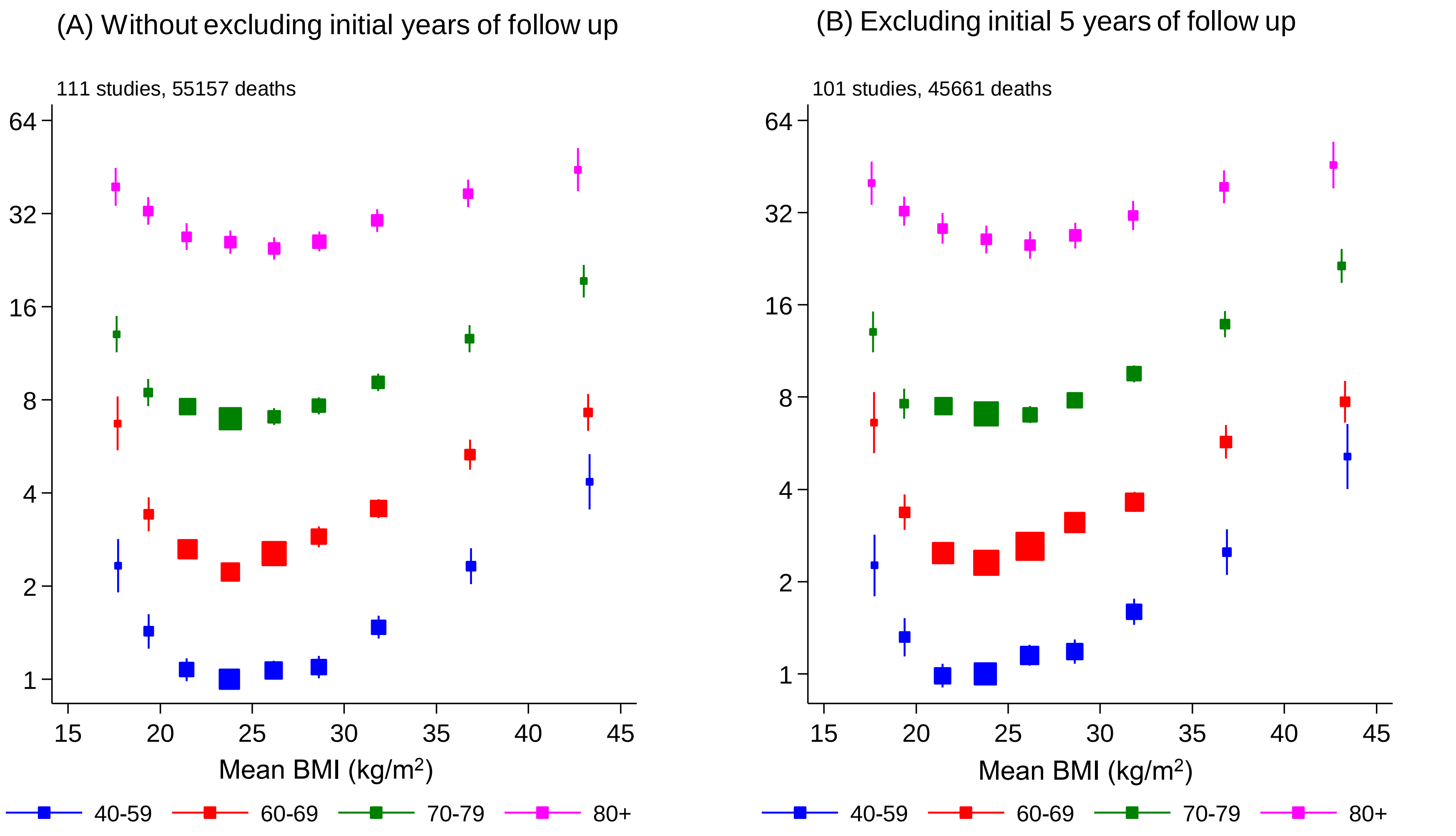


Analyses are restricted to never-smokers without pre-existing chronic disease, and exclude the first 5 years of follow-up.

BMI groupings: 15 to <18.5, 18.5 to <20, 20 to <22.5, 22.5 to <25, 25 to <27.5, 27.5 to <30, 30 to<35, 35 to<40, and 40 to <60 kg/$m^2$.

Reference category is BMI 22.5 to <25 kg/$m^2$.

Group-specific confidence intervals (CIs) were calculated using floating variance estimates (reflecting independent variability within each group, including the reference group).

Areas of squares are proportional to the information content in each.

**eFigure 8: Absolute risks corresponding to the overall relative risks**

## Effects of the Global BMI Consortium relative risks on absolute risks of death at ages 35-69, if absolute risks in the reference BMI category are 19% male and 11% female

(which are intermediate between the current risks in Western Europe and in North America)

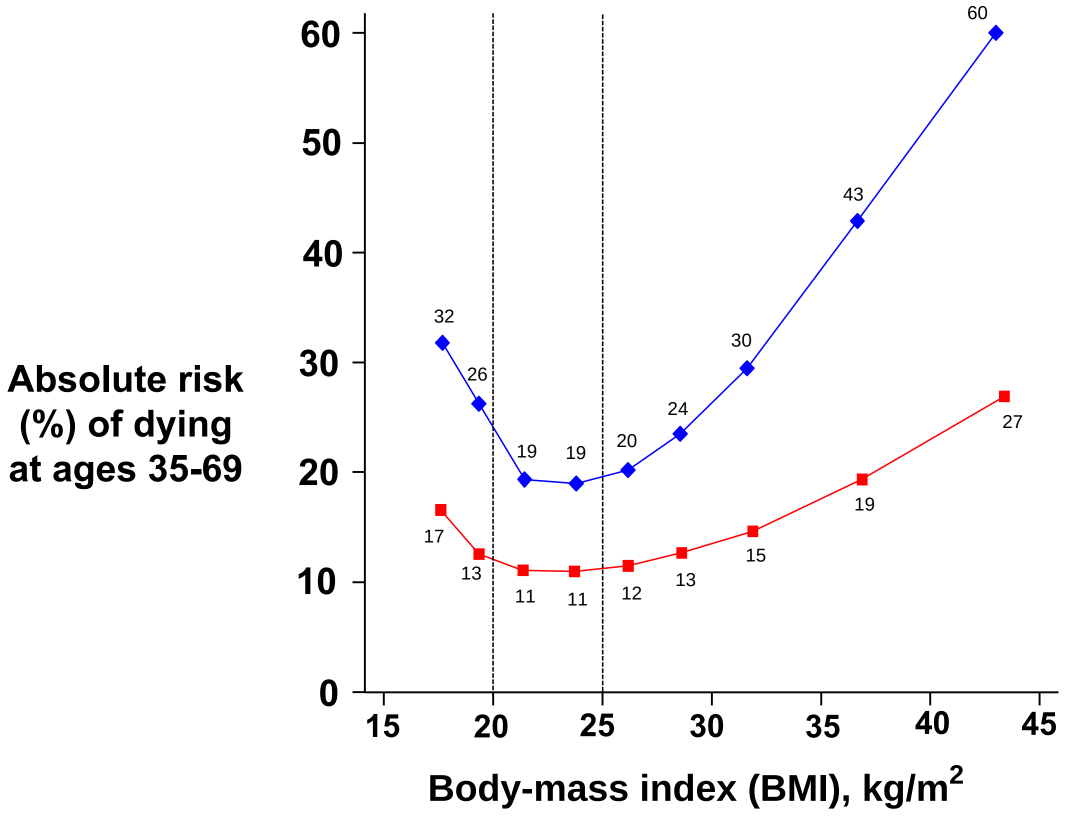


## Relative and absolute risks of death at ages 35-69 by BMI and by sex, if 35-year risk in the reference BMI category is 19% male, 11% female*

| BMI category, $kg/m^2$ | Male | | | Female | | |
|---|---|---|---|---|---|---|
| | Consortium death rate ratio, RR | Mean annual rate per 1000 | 35-year risk, % | Consortium death rate ratio, RR | Mean annual rate per 1000 | 35-year risk, % |
| **15-18.5 (underweight)** | 1.82 | 10.93 | 31.8 | 1.55 | 5.17 | 16.6 |
| **18.5-20** | 1.44 | 8.70 | 26.2 | 1.15 | 3.85 | 12.6 |
| **20-22.5** | 1.02 | 6.15 | 19.4 | 1.01 | 3.35 | 11.1 |
| **22.5 to <25 (reference)** | 1.00 | 6.02 | 19.0 | 1.00 | 3.33 | 11.0 |
| **25-27.5 (overweight)** | 1.07 | 6.47 | 20.3 | 1.05 | 3.49 | 11.5 |
| **27.5-30 (overweight)** | 1.27 | 7.66 | 23.5 | 1.16 | 3.87 | 12.7 |
| **30-35 (grade 1 obese)** | 1.66 | 9.97 | 29.5 | 1.36 | 4.52 | 14.6 |
| **35-40 (grade 2 obese)** | 2.66 | 16.02 | 42.9 | 1.85 | 6.15 | 19.4 |
| **40-60 (grade 3 obese)** | 4.35 | 26.17 | 60.0 | 2.69 | 8.95 | 26.9 |

* If the mean of age-specific death rates/1000 in the age range 35-69 is R, then 35-year risk (%) of dying in this age range is 100 – 100 exp (–35 R/1000). This yields the reference category death rates (from the risks of 19% and 11%). Multiplication by RRs then yields the other death rates, from which come 35-year risks.

**eFigure 9: Log$_e$ hazard ratio for all-cause mortality per 5 kg/m$^2$ higher BMI, plotted against mean BMI in each study**

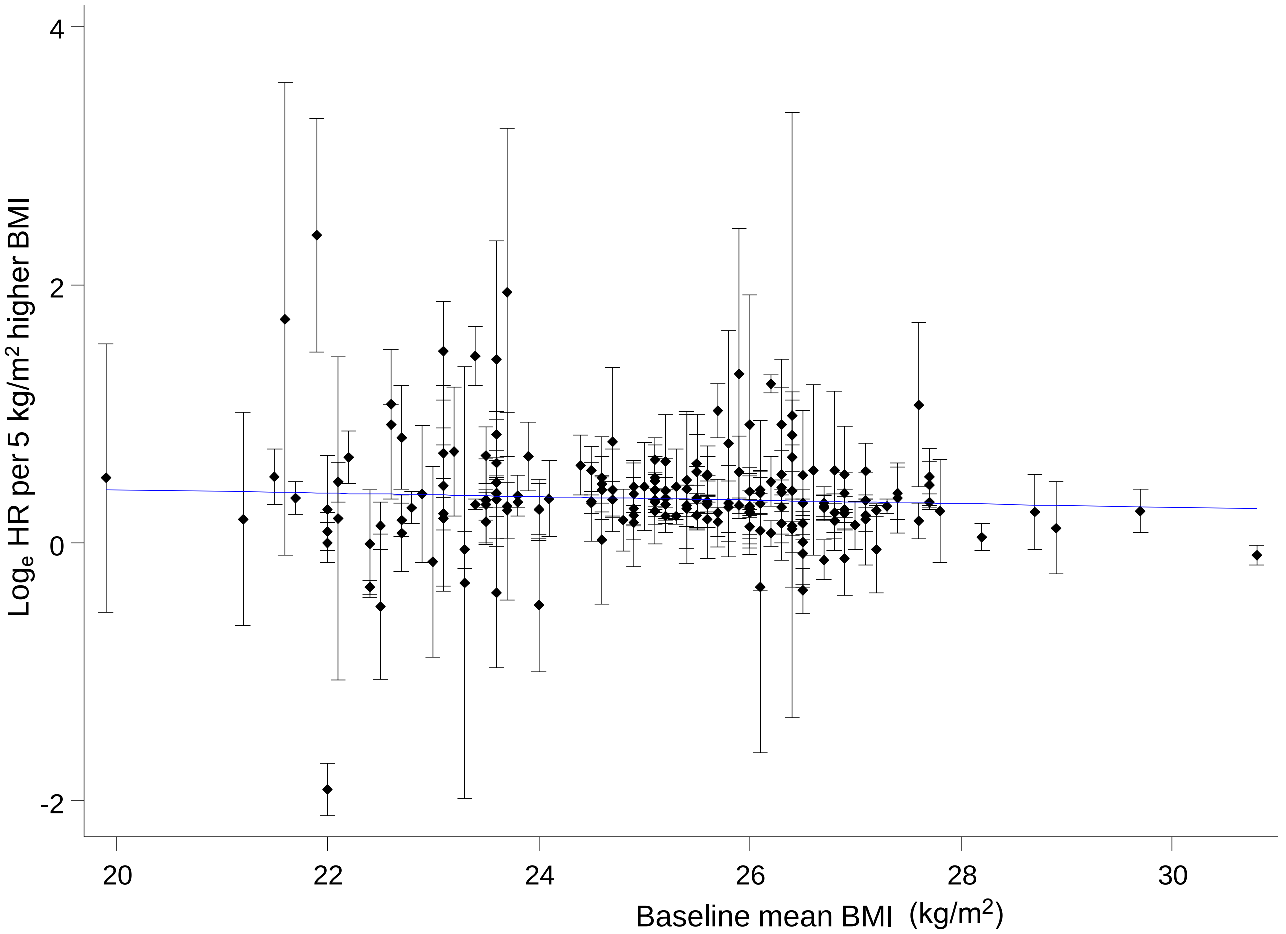


Analyses excluded the first 5 years of follow-up, and were restricted to never-smokers without pre-existing chronic disease.

HRs per 5 units of BMI were estimated by inverse-variance-weighted regression of the pooled log HR on BMI in the BMI range 25 to 60 kg/m$^2$.

Each point represents one study. The line has been fitted by meta-regression: P-value testing for non-zero slope = 0.431.

**eFigure 10: $Log_e$ hazard ratios for all-cause mortality per 5 $kg/m^2$ higher BMI, plotted against median year of baseline survey**

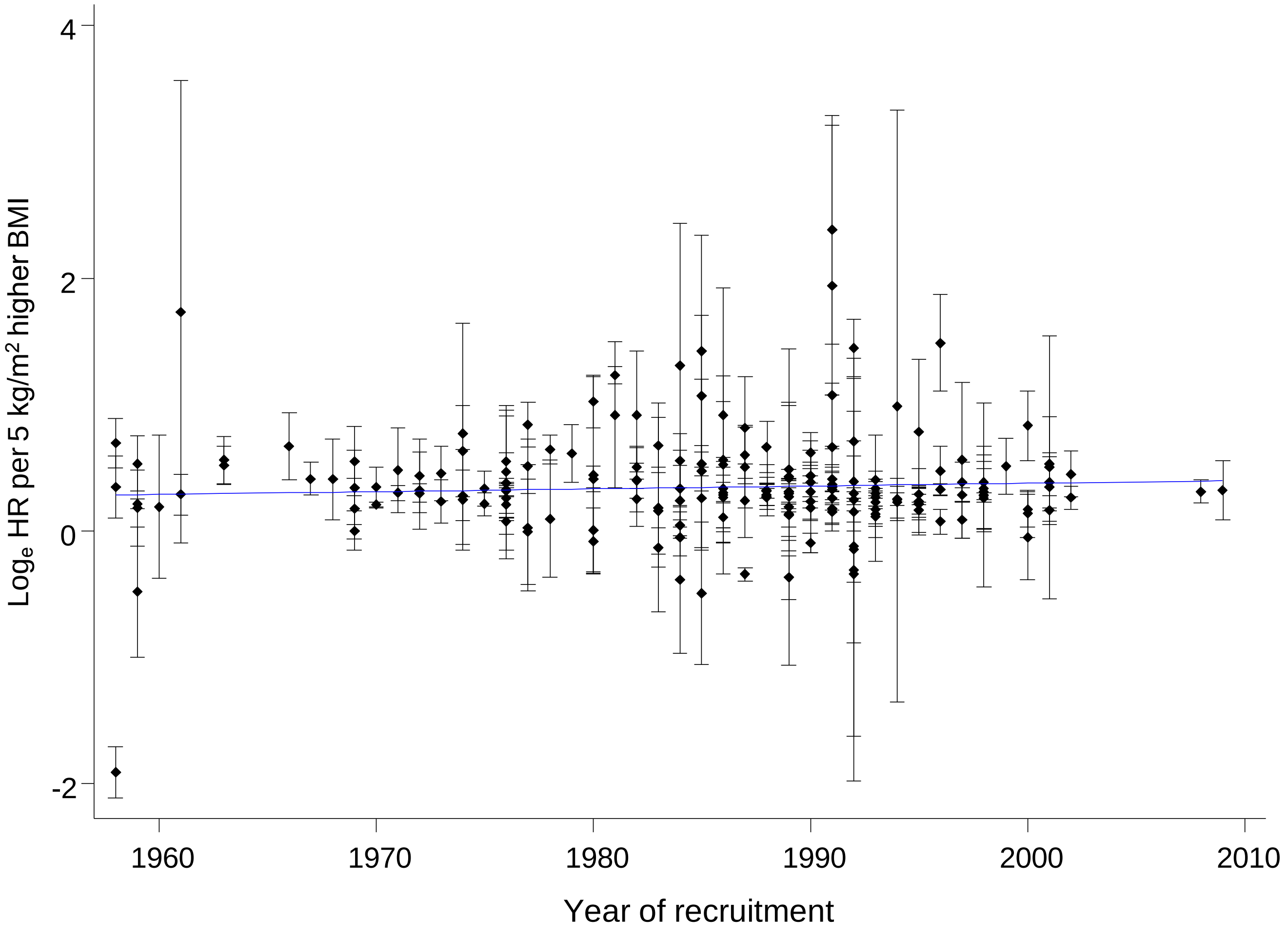


Analyses excluded the first 5 years of follow-up, restricted to never-smokers without pre-existing chronic disease.

HRs per 5 units of BMI were estimated by inverse-variance-weighted regression of the pooled log HR on BMI in the BMI range 25 to 60 $kg/m^2$.

Each point represents one study. The line has been fitted by meta-regression: P-value testing for non-zero slope = 0.491.

**eFigure 11: Log$_e$ hazard ratio for all-cause mortality per 5 kg/m$^2$ higher BMI, plotted against study size**

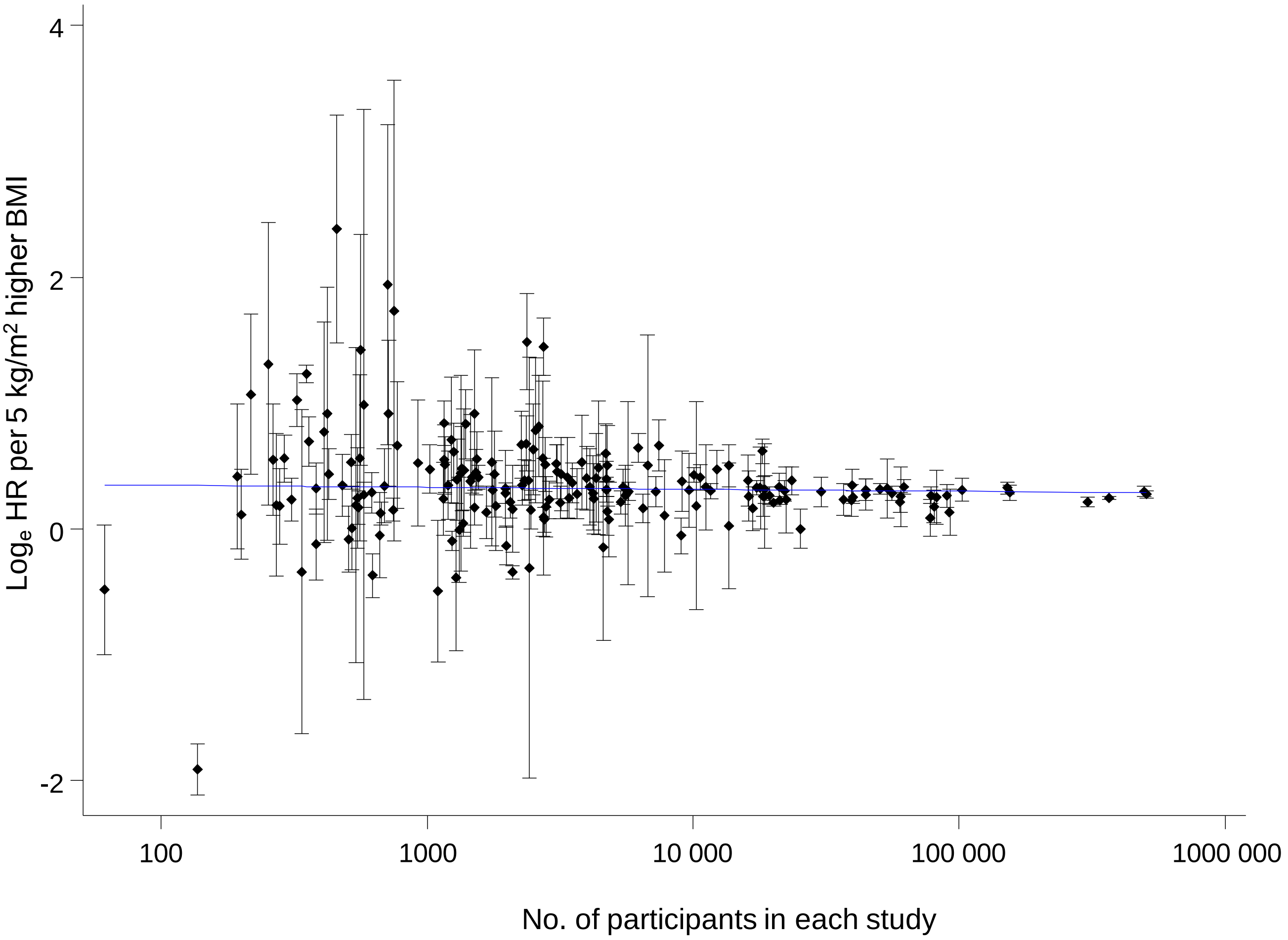


Analyses excluded the first 5 years of follow-up, and were restricted to never-smokers and people without pre-existing chronic disease.

HRs per 5 units of BMI were estimated by inverse-variance-weighted regression of the pooled log HR on BMI in the BMI range 25 to 60 kg/m$^2$.

Each point represents one study. The line has been fitted by meta-regression: P-value testing for non-zero slope = 0.615.

## eAppendix 1: Investigators in each study

**Abdominal Aortic Aneurysm Screening Program**: P Norman, K Jamrozik; **Agricultural Health Study**: L B Freeman, H Chen; **Air Force/Texas Coronary Atherosclerosis Prevention Study**: R W Tipping; **Aito Town Study**: A Okayama, H Ueshima, H Maegawa; **Akabane Study**: M Nakamura, N Aoki; **Antihypertensive and Lipid-Lowering Treatment to Prevent Heart Attack Trial**: B R Davis, L Simpson, S Pressel; **Beijing Anzhen Cohort Study**: C H Yao, Z S Wu; **Anzhen 02 Cohort Study**: Z S Wu; **Apolipoprotein Related Mortality Risk Study:** G Walldius, I Jungner; **Atherosclerosis Risk in Communities Study:** L Chambless, A Folsom, J Stevens, C Ndumele; **ATTICA Study:** D B Panagiotakos, C Pitsavos, C Chrysohoou; **Australian Diabetes, Obesity and Lifestyle Study:** D Magliano, A Peeters, J Shaw, E L M Barr; **Australian Longitudinal Study of Ageing**: M Luszcz, H Christensen; **Breast Cancer Detection Demonstration Project**: S C Moore; **Beijing Aging Study**: Z Tang; **Belgian Inter-university Research on Nutrition and Health**: G De Backer, D De Bacquer, M Kornitzer; **Bezafibrate Infarction Prevention Registry Study**: M Benderly; **British Regional Heart Study**: P H Whincup, S G Wannamethee, R Morris; **British Union Provident Association Study**: N Wald, J Morris, M Law; **British Women's Heart and Health Study**: J-P Casas, C Dale, DA Lawlor; **Bruneck Study**: S Kiechl, J Willeit, P Willeit; **Busselton Health Study**: M W Knuiman, M Divitini, H Bartholomew; **Caerphilly Prospective Study**: G Davey Smith, P Sweetnam, P Elwood, J Yarnell, J Gallacher; **California Teachers Study**: L Bernstein, H Anton-Culver; **Cancer Prevention Study I:** A V Patel, J S Hildebrand, B Carter, S M Gapstur; **Cancer Prevention Study II**: A V Patel, J S Hildebrand, S M Gapstur; **Capital Iron & Steel Company Study**: X G Wu; **Capital Iron & Steel Company Hospital Cohort**: J Zhou, X H Yu; **Cardiovascular Disease Risk Factors Two-Township Study**: W H Pan; **Cardiovascular Health Study (CHS1-CHS2)**: M Cushman, E Enright, R Kronmal; **Cardiovascular Study in the Elderly**: E Casiglia; **Consultation Bureau Project**: D Kromhout; **Centre d'Investigations Preventives et Cliniques (IPC), Paris**: A Benetos, L Guize(deceased); **Charleston Heart Study**: P J Nietert, S Sutherland, J Keil; **Chicago Heart Association Detection Project In Industry**: P Greenland; **China Hypertension Survey Epidemiology Follow-up Study**: J He; **China Prospective Study**: M Smith, G Yang, M Zhou, L Yang, G Whitlock, Z Chen; **Civil Service Workers Study**: A Tamakoshi; **CLUE II**: K Visvanathan, J Hoffman-Bolton; **Cohort of Norway (FINNMARK)**: R Selmer, A J Sögaard, L L Håheim, H E Meyer, W Nystad; **Cohort of Norway (HUBRO)**: R Selmer, A J Sögaard, L L Håheim, H E Meyer, W Nystad; **Cohort of Norway (OPPHED)**: R Selmer, A J Sögaard, L L Håheim, H E Meyer, W Nystad; **Cohort of Norway (OSLO2)**: R Selmer, A J Sögaard, L L Håheim, H E Meyer, W Nystad; **Cohort of Norway (TROMS)**: R Selmer, A J Sögaard, L L Håheim, H E Meyer, W Nystad; **Cohort of Swedish Men**: N Hakansson; **Community-based Cancer Screening Project**: C J Chen; **Copenhagen City Heart Study**: B G Nordestgaard, A Tybjaerg-Hansen, R Frikke-Schmidt, G Jensen, P Schnohr; **Data from an Epidemiological Study on the Insulin Resistance Syndrome**: B Balkau, R Roussel, F Fumeron; **Diet and Risk of Cardiovascular Disease in Spain**: M A Rubio-Herrera, J A Gutiérrez-Fuentes, J A Gómez-Gerique, A G de la Cámara; **Dubbo Study of the Elderly**: L A Simons; **East Beijing Cohort Study**: Z L Wu, L Q Chen, G L Shan; **Edinburgh Artery Study**: J F Price, S Mclachlan; **Electrical Generating Authority of Thailand Study**: P Sritara; **Epidemiologische Studie zu Chancen der Verhütung**: B Schöttker, K-U Saum, B Holleczek, H Brenner, D Rothenbacher, V Arndt; **Established Populations for the Epidemiologic Study of the Elderly Studies: East Boston** J O Taylor, **Iowa** R B Wallace, **New Haven** C L Phillips, **North Carolina** G Fillenbaum, D G Blazer; **European Prospective Investigation into Cancer and Nutrition:** B B de Mesquita, T Pischon, E Lund, A Cross, K T Khaw, N J Wareham, Y T Van Der Schouw, S Törnberg, J Carstensen; **Fangshan Cohort Study:** D F Gu, X F Duan; **Finnish Mobile Clinic Survey:** A Aromaa, P Knekt, A Reunanen; **Finrisk 1977**: J Tuomilehto, P Jousilahti, E Vartiainen, P Puska; **Finrisk 1982**: J Tuomilehto, P Jousilahti, E Vartiainen, P Puska; **Finrisk Cohort 1987**: P Jousilahti, S Männistö, M Peltonen; **Finrisk Cohort 1992**: V Salomaa, K Harald, K Borodulin, M Perola; **Finrisk Cohort 1997**: V Salomaa, A Juolevi, A Jula; **Flemish Study on Environment, Genes and Health (FLEMENGHO)**: T Kuznetsova, T Richart, J Staessen, L Thijs; **Fletcher Challenge Heart and Health Study**: M Woodward, S MacMahon, R Norton, S Ameratunga, G Whitlock, R Jackson; **Framingham Offspring Study**: R B D'Agostino Sr;**Funagata Study**: M Daimon, T Oizumi, T Kato, T Kayama; **Glucose Intolerance, Obesity and Hypertension Study**: R Dankner, A Chetrit, F Lubin; **Goteborg 1913 Study**: L Wilhelmsen; **Goteborg 1933 Study**: A Rosengren, L Wilhelmsen, L Welin ; **Goteborg 1943 Study**: A Rosengren, L Wilhelmsen,; **Göttingen Risk Incidence and Prevalence Study**: D Nagel; **Guangzhou Occupational Cohort Study**: R Dankner, A Chetrit, F Lubin, T H Lam, C Q Jiang, Y He,; **Health Effects of Arsenic Longitudinal Study**: H Ahsan; **Health in Men Study**: L Flicker, B B Yeap; **Health Professionals Follow-up Study**: WC Willett, A Flint and E B Rimm; **Helsinki Aging Study**: R S Tilvis, T E Strandberg; **Helsinki Businessmen Study**: T E Strandberg, V Salomaa, R S Tilvis; **Hertfordshire Cohort Study**: C Cooper, E Dennison, A A Sayer, M Edwards; **Hisayama Study**: Y Kiyohara, H Arima, T Ninomiya, M Lida; **Hong Kong Study**: J Woo, S C Ho; **Honolulu Heart Program**: B Rodriguez, D Sharp, J D Curb; **Hoorn Study**: C D A Stehouwer, J M Dekker, G Nijpels; **Huashan Study**: Z Hong, M S Huang, B Zhou; **Ibaraki Prefectural Health Study**: F Irie; **Ikawa, Kyowa, Noichi Study**: S Sato, H Iso, A Kitamura, Y Naito; **Iowa Women's Health Study**: J R Cerhan, D Lazovich; **Israeli Ischaemic Heart Disease Study**: U Goldbourt; **Japan Collaborative Cohort Study**: A Tamakoshi; **Japan Public Health Center–based Prospective Study 1**: M Inoue; **Japan Public Health Center–based Prospective Study 2**: S Tsugane; **Japan Railways**: M Tomita, Y Nishimoto, T Murayama; **The Kinmen Neurological Disorders Survey**: J L Fuh; **Konan Health and Nutrition Study**: H Ueshima, Y Kita, S R Choudhury; **Korean Cancer Prevention Study:** J M Samet, K J Jung, Y D Yun, S H Jee; **Korean Multi-center Cancer Cohort Study**: D Kang; **Korean Medical Insurance Corporation Study**: I Suh, S H Jee, I S Kim; **Kuopio Ischaemic Heart Disease Study**: J T Salonen, J Kauhanen, T-P Tuomainen, J Virtanen; **Kyowa Study**: H Iso, S Sato, A Kitamura, Y Naito; **Life Span Study Cohort**: E Grant; **Lipid Research Clinics Follow-up Study**: M Criqui, C Davis; **Longitudinal Aging Study Amsterdam**: M Visser, D J H Deeg, J L Poppelaars; **Lower Extremity Arterial Disease Event Reduction Trial**: T W Meade; **Malmö Prevention Project**: B Hedblad, P Nilsson, O Melander, G Engström; **Melbourne Collaborative Cohort Study**: G G Giles, D English; **Midspan Collaborative Study**: O Melander, C Hart, G Davey Smith, D Hole(deceased), C Gillis; **Midspan Family Study**: A McConnachie, N Sattar, G Watt; **The Million Women Study**: A Balkwill B J Cairns, G K

Reeves, J Green, V Beral, The Million Women Study Collaborators ( www.millionwomenstudy.org); **Minnesota Heart Health Project**: D Jacobs, H Blackburn, R Luepker; **Minnesota Heart Survey**: D Jacobs, H Blackburn, R Luepker; **Miyagi Cohort:** Y Nishino; **Miyama Cohort Study**: T Hashimoto, K Sakata; **MONICA Göteborg Study**: A Rosengren; **MONICA/KORA Augsburg Survey 1**: W Koenig, C Meisinger; **MONICA/KORA Augsburg Survey 2**: C Meisinger, W Koenig; **MONICA/KORA Augsburg Survey 3**: W Koenig, C Meisinger; **Monitoring of CVD Risk Factors Project**: W M Verschuren; **MRC Study of Older People:** A Fletcher; **Multi-Ethnic Study of Atherosclerosis**: S J Shea, K E Watson, I De Boer, A C Armstrong; **Multiple Risk Factor Intervention Trial**: L H Kuller, J Neaton, L Eberly; **Mumbai Cohort Study**: P C Gupta, M S Pednekar; **National Health and Nutrition Examination Survey I:** R F Gillum, B Howard, J Umans, J R Albertorio-Díaz, C Cox; **National Health and Nutrition Examination Survey III**: J R Albertorio-Díaz, R F Gillum; **National Health Interview Survey:** C L Jackson; **National Heart Foundation Risk Factor Prevalence Survey 1989**: T A Welborn; **Newcastle Study**: A Dobson; **NHLBI Framingham Heart Study**: D Levy, R B D'Agostino Sr, H Silbershatz; **NIH-AARP Diet and Health Study**: Y Park, R Sinha; **Noichi Study**: H Iso, S Sato, A Kitamura, Y Naito; **North Karelia Project**: V Salomaa, P Puska, E Vartiainen, T Laatikainen; **Northwick Park Heart Study I**: T Meade, K Garrow, J Cooper; **Northwick Park Heart Study II**: J A Cooper; **Norwegian Counties Study (NCS1):** R Selmer, A Tverdal; **Norwegian Counties Study (NCS2)**: R Selmer, A Tverdal; **Norwegian Counties Study (NCS3)**: R Selmer, A Tverdal; **Nova Scotia Health Survey**: J A Shaffer, K W Davidson, S Kirkland; **Nurses' Health Study I:** JoAnn E Manson, Frank Hu, and Meir J Stampfer; **Nurses' Health Study II:** H Eliassen, W C Willett, S Hankinson; **Nutrition Intervention Trials - Linxian**: Y Qiao; **NYU Women's Health Study**: A Zeleniuch-Jacquotte; **Occupational Groups (OG), Rome**: A Menotti, A Spagnolo; **Ohasama Study**: Y Imai, T Ohkubo, A Hozawa, I Tsuji, S Hisamichi; **Ohsaki National Health Insurance Cohort Study**: Y Tomata; **Osaka Study**: S Sato; **Oslo Study**: I Holme, A Tverdal, L L Haheim, I Hjermann, P Leren; **Oyabe study**: H Nakagawa, M Sakurai; **Paris Prospective Study I**: P Ducimetiere, J Empana; **Perth Risk Factors Survey:** K Jamrozik, M Hobbs, R Broadhurst; **Physicians' Health Study I & II**: J M Graziano, H D Sesso, P M Ridker, J E Buring, C Hennekens; **Prostate, Lung, Colorectal and Ovarian (PLCO) Cancer Screening Trial**: B Wilcox; **Prospective Study of Women in Gothenburg**: C Bengtsson, C Björkelund, L Lissner; **Prevention of Renal and Vascular End Stage Disease Study**: S J L Bakker, R Gansevoort, B Wolffenbuttel; **Progetto CUORE (ATENA)**: S Giampaoli, L Palmieri, D Vanuzzo, S Panico, C Donfrancesco, L Pilotto; **Progetto CUORE (EMOFRI, MATISS83, MATISS87, MATISS93, MONFRI86, MONFRI89, MONFRI94):** S Giampaoli, L Palmieri, D Vanuzzo, S Panico, C Donfrancesco, L Pilotto; **Prospective Cardiovascular Münster Study**: G Assmann, H Schulte; **Prospective Epidemiological Study of Myocardial Infarction**: G Assmann, H Schulte; **Prospective Study of Pravastatin in the Elderly at Risk**: N Sattar, J W Jukema; **Puerto Rico Heart Health Program**: C J Crespo, P Sorlie, M Garcia-Palmieri; **Quebec Cardiovascular Study**: P Lupien, N M Robitaille, B Cantin; **Rancho Bernardo Study**: H Araneta, D Kritz-Silverstein, E Barrett-Connor, M Criqui, R Langer; **Renfew and Paisley Study**: C Hart, G Davey Smith, D Hole(deceased); **Research Centre for Prevention and Health (Glostrup Population Studies):** T Jørgensen, T Thomsen, J Jespersen; **Reykjavik Study**: V Gudnason, T Aspelund, B Thorsson; **Risk Factors and Life Expectancy Pooling Project (ATS_SAR)**: M Trevisan; **Risk Factors and Life Expectancy Pooling Project (DISCO)**: M Trevisan; **Risk Factors and Life Expectancy Pooling Project (GREPCO):** M Trevisan; **Risk Factors and Life Expectancy Pooling Project (GUBBIO)**: M Trevisan; **Risk Factors and Life Expectancy Pooling Project (MICOL)**: M Trevisan; **Risk Factors and Life Expectancy Pooling Project (MONICA)**: M Trevisan; **Risk Factors and Life Expectancy Pooling Project (NFR)**: M Trevisan; **Risk Factors and Life Expectancy Pooling Project (OB43)**: M Trevisan; **Risk Factors and Life Expectancy Pooling Project (RF2)**: M Trevisan; **The Rotterdam Study**: O H Franco, K Dhana, M Kavousi, A Hofman, J Kiefte-de Jong; **The Rotterdam Study II**: O H Franco, T Muka, H W Tiemeier, F van Rooij; **The Rotterdam Study III**: O H Franco, A Hofman, J D Schoufour, J C Kiefte-de Jong; **Saitama Cohort Study**: K Nakachi, K Imai; **Scottish Heart Health Study**: H Tunstall-Pedoe, M Woodward; **Seoul Male Cancer Cohort**: Y-O Ahn; **Seven Cities Cohorts Study:** X H Fang, S C Li, Q D Yang; **Seven Countries Croatia**: R Buzina; **Seven Countries Finland**: A Nissinen, H Tolonen; **Seven Countries Greece**: C Aravanis, A Dontas, A Kafatos; **Seven Countries Italy**: A Menotti; **Seven Countries Japan**: H Adachi, H Toshima, T Imaizumi; **Seven Countries Netherlands**: D Kromhout, J M Geleijnse; **Seven Countries Serbia**: S Nedeljkovic, M Ostojic; **Seven Countries US**: D Jacobs, H Blackburn; **Seventh-day Adventist Cohort Study-1**: W L Beeson; **Shanghai Cohort Study**: J-M Yuan, Z M Chen; **Shanghai Men's Health Study**: X-O Shu; **Shanghai Women's Health Study**: Y-T Gao; **Shibata Cohort Study**: H Tanaka, T Nakayama, N Yoshiike, T Yokoyama, C Date; **Shigaraki Town Study**: Y Kita, A Nozaki, H Ueshima; **Shirakawa Study**: H Horibe, Y Matsutani, M Kagaya; **Singapore Chinese Health Study**: W P Koh; **Singapore Thyroid and Heart Study**: K Hughes, J Lee; **Singapore National Health Survey 1992**: D Heng, S K Chew; **Six Chinese Cohorts Study**: B F Zhou, H Y Zhang; **Speedwell Study:** Y Ben-Shlomo, J Gallacher, J Yarnell, G Davey Smith; **Strong Heart Study**: B V Howard, L G Best, M Begum; **Study of Health in Pomerania**: H Völzke; **Swedish Mammography Cohort**: A Wolk; **Tanno-Soubetsu Study**: K Shimamoto, S Saitoh; **Tecumseh Study**: J Keller; **Three Prefecture Study Aichi**: H Tanaka; **Three Prefecture Study Miyagi**: I Tsuji; **45 and Up Study:** G Joshy, R J Korda, E Banks; **Thrombosis Prevention Trial**: T W Meade; **Tianjin Study**: Z Z Li, H Y Zhang; **Toyama Study**: Hidaeki Nakagawa, M Sakurai; **Trivandrum Oral Cancer Screening Trial:** K Ramadas; **Tromsø Study**: I Njølstad, K Bonaa, E Arnesen; **Turkish Adult Risk Factor Study**: A Onat, G Can, H Yuksel; **United Kingdom Heart Disease Prevention Project**: H Tunstall-Pedoe; **Uppsala Longitudinal Study of Adult Men**: J Sundström, J Ärnlöv, U Risérus; **U.S.Radiologic Technologists (URST) Cohort**: C Kitahara; **Vicenza Thrombophilia and Athrosclerosis Project**: A Tosetto, F Rodeghiero; **Vitamins and Lifestyle (VITAL) Study:** E White; **Vorarlberg Health Monitoring and Promotion Programme**: H Ulmer, H Concin, G Nagel; **West of Scotland Coronary Prevention Study**: J W Jukema, I Ford; **Whitehall I Study**: M Kivimaki, M G Marmot, E Brunner, M J Shipley, A Fletcher, D Leon; **Whitehall II Study**: E J Brunner; **Women's Lifestyle and Health Study:** E Weiderpass; **Women's Health Initiative (Observational Study)**: S Wassertheil-Smoller; **Women's Health Study**: P M Ridker; **Württemberg Construction Workers Cohort**: V Arndt, H Brenner, D Rothenbacher; **Yunnan Tin Miner Cohort Study**: S X Yao; **Zaragoza Study**: A M Ibañez.

**eAppendix 2: Pre-specified analysis plan**

**1. General methodological considerations**

*1.1 Participating consortia/cohort studies* Consortia / large studies that had agreed to collaborate in this effort are listed below.

Main exclusions: Participants with BMI<15 and BMI>60 kg/m$^2$ will be excluded. In addition, participants of age <20 years and ⩾90 years at baseline will be excluded. Primary analyses will also exclude current and former smokers, and the first 5 years of follow-up.

*1.2 Exposures and outcomes* BMI will be pre-defined in the following categories: 15 to <18.5, 18.5 to <20, 20 to <22.5, 22.5 to <25 (reference category), 25 to <27.5, 27.5 to <30, 30 to <35, 35 to <40, 40 to 60 kg/m$^2$. Furthermore, to enable direct comparison with the Flegal et al meta-analysis results, BMI will also be categorized according to standard cut-points: 15 to <18.5, 18.5 to <25 (reference category), 25 to <30, 30 to <35, 35 to <40, 40 to 60 kg/m$^2$. The primary outcome of the analysis will be all-cause mortality. Secondary outcomes will include: cause-specific mortality, including mortality due to cancer, cardiovascular disease, CHD, stroke, and respiratory disease.

*1.3 Unit of analysis* The following advantages will derive from basing this meta-analysis on cohort-level (rather than consortium-level) estimates for the pooled analyses. First, this approach should enable appropriate pooling of the log hazard ratio (HR) estimates across different studies. Second, it should facilitate reliable investigation of BMI-mortality associations under different circumstances (eg, by different geographical regions, sex, and at different ages). Third, it should enable the reliable quantification of between-study heterogeneity. As the accrual of these advantages will be important to demonstrate that the present analysis represents a clear and significant methodological improvement over the methods used by Flegal et al, this approach has been strongly encouraged. Hence, to facilitate such analyses (and to enable a uniform approach to analyses across the participating consortia/studies), the Coordinating Center will provide STATA software scripts for direct use (or adaptation as needed). For example, given the categorical nature of the primary exposure, it is essential to obtain the full covariance matrix of the log HR estimates within each cohort to present relative risk as floating absolute risks and to facilitate more appropriate pooling using multivariate meta-analysis.

*1.4 Avoidance of "double-counting" data from participating studies* As some studies to be included in the present analysis have provided data on BMI and mortality outcomes to two or more of the participating consortia, it is important to ensure that data from such studies are not "double-counted" in the present meta-analysis. This will be achieved in consultation with coordinators of the participating consortia. In such situations, the information from the consortium to which the study has provided the maximum relevant data will provide results for that cohort.

*1.5 Subgroup analysis* Associations of all-cause mortality with BMI depend not only on the associations of specific causes of death with BMI in different regions (which might differ quantitatively), but also on how relatively common each specific cause of death is in the particular region (which can differ very substantially by region over time). Because of the latter point, there is no reason to expect a priori that the association of all-cause mortality with BMI will be similar in regions that have different underlying mortality patterns. Therefore, the primary analysis will be stratified by major geographical regions (ie, Europe/North America/Australia/NZ, South Asia, and East Asia). Furthermore, previous large-scale epidemiological studies have suggested that the association between BMI and all-cause mortality varies across different age groups, and different approaches used to assess BMI (ie, directly measured vs self-reported). It is therefore important to investigate the effect of BMI on mortality risk within geographical regions according to these different individual and study-level characteristics. Therefore, the meta-analysis will assess the association between BMI categories and all-cause mortality by sex-specific age groups (ie, 35 to 49, 50 to 59, 60 to 69, 70 to 79, 80 to 89 years of age at baseline), smoking status (never smokers only), and BMI assessment methods (self-reported vs. measured).

## 2. Statistical analyses plan

1. Each cohort lists whether information on the following chronic diseases were recorded at baseline examination: (i) cardiovascular disease (ie, myocardial infarction, stroke, CABG, angioplasty, peripheral artery disease, congestive heart failure); (ii) cancer (not including non-melanoma skin cancer); (iii) respiratory diseases (eg, COPD); and (iv) neurodegenerative diseases (ie, Parkinson's disease, Alzheimer's disease). We acknowledge that not all studies have full information on these chronic diseases at baseline. However, a table providing the specific diseases recorded at baseline by each study will clarify the exclusions made in the main analyses.

2. The following cohort-level characteristics at baseline will be provided in
(i) people without chronic disease at baseline (as defined in #1) and
(ii) all people (ie, without exclusion of people with known disease at baseline) (as a secondary analysis for comparison purpose):

a. Total number of participants
b. Mean (SD) of BMI at baseline
c. Mean (SD) of age at baseline.
d. Mean (SD) of age at death.
e. Number (%) of males.
f. Number (%) of current tobacco smokers.
g. Median (IQR) of baseline survey year.
h. Median (IQR) of follow-up time.
i. Total number of deaths (incl. specifically deaths from cardiovascular disease, CHD, stroke, cancer, and respiratory disease).
j. Country and region (ie, Europe, North America, South Asia, East Asia, Australia/NZ).
k. Method of assessment of height and weight (ie, self-reported or directly measured).
l. Ascertainment methods for (if possible, underlying) cause of death (death certificate only, or death certificate supplemented by medical record).
m. Details of censoring for cases and follow-up.

3. Primary analyses will be in pre-defined BMI categories [15 to <18.5, 18.5 to <20, 20 to <22.5, 22.5 to <25 (reference category), 25 to <27.5, 27.5 to <30, 30 to <35, 35 to <40, 40 to 60 $kg/m^2$].

Parallel analyses will use standard BMI categories [15 to <18.5, 18.5 to <25 (reference category), 25 to <30, 30 to <35, 35 to <40, 40 to 60 $kg/m^2$].

Both analyses will be conducted in people without chronic disease at baseline (as defined in #1).

4. To investigate measurement error in measured and self-reported BMI, each cohort provides mean (SD) BMI values at baseline and, if available, at re-survey in studies with measured BMI, within each category of baseline BMI.

5. For each analysis in #7 and #8, studies provide information on the number of participants and number of deaths within each of these categories.

6. Analyses are based on Cox proportional hazards (PH) models estimated for each study separately, using attained age (equivalent to calendar age or age-at-risk) as the underlying time scale.

All PH models are stratified by sex and by these age groups (20 to 34, 35 to 49, 50 to 59, 60 to, 70 to 79, 80 to 89 years of age at baseline).

PH models are adjusted for smoking status (current tobacco and the number of cigarette-equivalents per day [or pack-years] if available/ex/never; or if not possible: current/other).

Studies contributing <10 deaths to a specific analysis will be excluded from that particular analysis to avoid distortions (eg, non-convergence of statistical models) that can appear in analyses involving small strata. HRs will be presented as floating absolute risks.

7. The following analyses are restricted to participants without known history of chronic disease at baseline (as defined in #1):

7a. Study-specific HRs (including standard errors and covariances) will be calculated for the association between BMI categories and all-cause mortality:
i. Stratified by sex and age groups (at baseline), without excluding any follow-up.
ii. Adjusted for smoking, and stratified by sex and age groups, without excluding any follow-up.
iii. Adjusted for smoking, and stratified by sex and age groups, excluding the first 5 years of follow-up. (Optional analyses can also adjust for confounding variables, such as education and marital status, and urban/rural resident).
iv. Stratified by sex and age groups, excluding current and former smokers.
v. Stratified by sex and age groups, excluding current and former smokers and the first 5 years of follow-up (primary analysis).

7b. The 5 analyses described in #7a will be repeated to calculate HRs between BMI categories and all-cause mortality, according to different individual and study-level characteristics:
i. HRs by subgroup of geographical region (no extra analyses required) (primary analysis).
ii. HRs by subgroup of sex and age combined (35 to 49, 50 to 59, 60 to 69, 70 to 79, 80 to 89 years of age at baseline) ie, fitting 10 separate PH models within each sex-specific age group.
iii. If possible, HRs by ethnicity (fitting separate PH models within each ethnicity group).
iv. HRs by subgroup of assessment method (no extra analyses required).

7c. The 5 analyses described in #7a will be repeated to calculate HRs for the association between BMI categories and all-cause mortality, restricting follow-up to up to 15 years.

7d. The 5 analyses described in #7a will be repeated to calculate HRs for the association of BMI categories, where available, with risk of death from
(i) cardiovascular disease,
(ii) CHD,
(iii) stroke,
(iv) cancer, and
(v) respiratory disease,
each censoring deaths from other causes.

8. The 5 analyses described in #7a will be repeated in all participants (without exclusion of people with known disease at baseline) to calculate HRs for the association between BMI categories and all-cause mortality.

9. Study-specific log HRs will be meta-analyzed by geographical regions using multivariate random-effects meta-analysis. Between-study heterogeneity will be quantified by the $I^2$ statistic. Supplementary analyses will combine log HRs using multivariate fixed-effect meta-analysis.

10. If required, consortia/studies will be contacted for further information or analyses.

**3. List of consortia / large studies that had agreed to collaborate in this effort**

Asia Cohort Consortium, Asia Pacific Cohort Studies Collaboration, Cancer Prevention Study I, Cancer Prevention Study II, China Prospective Study, Emerging Risk Factors Collaboration, European Prospective Investigation into Cancer and Nutrition (EPIC), National Cancer Institute (NCI) Cohort Consortium, Korean Cancer Prevention Study, Million Women Study, National Health Interview Survey, Nurses' Health Study II, Prospective Studies Collaboration, UK Biobank, 45 and Up

(listed in alphabetical order – updated July 2015)

## eAppendix 3: PRISMA-IPD checklist

| PRISMA-IPD Section/Topic | Checklist item | Reported on page |
|---|---|---|
| **Title** | | |
| Title | Identify the report as a systematic review and meta-analysis of individual participant data. | 1 |
| **Abstract** | | |
| Structured summary | Provide a structured summary including as applicable: | |
| | Background: state research question and main objectives, with information on participants, interventions, comparators, and outcomes. | 3 |
| | Methods: report eligibility criteria; data sources including dates of last bibliographic search or elicitation, noting that IPD were sought; methods of assessing risk of bias. | 3 |
| | Results: provide number and type of studies and participants identified and number (%) obtained; summary effect estimates for main outcomes (benefits and harms) with confidence intervals and measures of statistical heterogeneity. Describe the direction and size of summary effects in terms meaningful to those who would put findings into practice. | 3 |
| | Discussion: state main strengths and limitations of the evidence, general interpretation of the results, and any important implications. | 3 |
| **Introduction** | | |
| Rationale | Describe the rationale for the review in the context of what is already known. | 4 |
| Objectives | Provide an explicit statement of the questions being addressed with reference, as applicable, to participants, interventions, comparisons, outcomes, and study design (PICOS). Include any hypotheses that relate to particular types of participant-level subgroups. | 4 |
| **Methods** | | |
| Protocol and registration | Indicate if a protocol exists and where it can be accessed. If available, provide registration information including registration number and registry name. Provide publication details, if applicable. | 5 and eAppendix 2 |
| Eligibility criteria | Specify inclusion and exclusion criteria including those relating to participants, interventions, comparisons, outcomes, study design, and characteristics (eg, years when conducted, required minimum follow-up). Note whether these were applied at the study or individual level, ie, whether eligible participants were included (and ineligible participants excluded) from a study that included a wider population than specified by the review inclusion criteria. The rationale for criteria should be stated. | 5 and eAppendix 4 |
| Identifying studies: information sources | Describe all methods of identifying published and unpublished studies including, as applicable: which bibliographic databases were searched with dates of coverage; details of any hand searching including of conference proceedings; use of study registers and agency or company databases; contact with the original research team and experts in the field; open advertisements; and surveys. Give the date of last search or elicitation. | 5, eAppendix 4, and eFigure 1 |
| Identifying studies: search | Present the full electronic search strategy for at least 1 database, including any limits used, such that it could be repeated. | eAppendix 4 |
| Study selection processes | State the process for determining which studies were eligible for inclusion. | 5, eAppendix 2, and eAppendix 4 |
| Data collection processes | Describe how IPD were requested, collected, and managed, including any processes for querying and confirming data with investigators. If IPD were not sought from any eligible study, the reason for this should be stated (for each such study). | 5, eAppendix 2, and eAppendix 4 |
| | If applicable, describe how any studies for which IPD were not available were dealt with. This should include whether, how, and what aggregate data were sought or extracted from study reports and publications (such as extracting data independently in duplicate) and any processes for obtaining and confirming these data with investigators. | 5, 6, 7 and eFigure 1 |
| Data items | Describe how the information and variables to be collected were chosen. List and define all study-level and participant-level data that were sought, including baseline and follow-up information. If applicable, describe methods of standardizing or translating variables within the IPD data sets to ensure common scales or measurements across studies. | eAppendix 2 |
| IPD integrity | Describe what aspects of IPD were subject to data checking (such as sequence generation, data consistency and completeness, baseline imbalance) and how this was done. | eAppendix 2 |
| Risk of bias assessment in individual studies | Describe methods used to assess risk of bias in the individual studies and whether this was applied separately for each outcome. If applicable, describe how findings of IPD checking were used to inform the assessment. Report if and how risk of bias assessment was used in any data synthesis. | eAppendix 2 |
| Specification of outcomes and effect measures | State all treatment comparisons of interest. State all outcomes addressed and define them in detail. State whether they were prespecified for the review and, if applicable, whether they were primary/main or secondary/additional outcomes. Give the principal measures of effect (such as risk ratio, hazard ratio, difference in means) used for each outcome. | 5, 6, 7 and eAppendix 2 |

| | | |
|---|---|---|
| Synthesis methods | Describe the meta-analysis methods used to synthesize IPD. Specify any statistical methods and models used. | 6, 7 |
| Exploration of variation in effects | If applicable, describe any methods used to explore variation in effects by study- or participant-level characteristics (such as estimation of interactions between effect and covariates). State all participant-level characteristics that were analyzed as potential effect modifiers and whether these were prespecified. | 6, 7 |
| Risk of bias across studies | Specify any assessment of risk of bias relating to the accumulated body of evidence, including any pertaining to not obtaining IPD for particular studies, outcomes, or other variables. | NA |
| Additional analyses | Describe methods of any additional analyses, including sensitivity analyses. State which of these were prespecified. | 6, 7 and eAppendix 2 |
| **Results** | | |
| Study selection and IPD obtained | Give numbers of studies screened, assessed for eligibility, and included in the systematic review with reasons for exclusions at each stage. Indicate the number of studies and participants for which IPD were sought and for which IPD were obtained. For those studies for which IPD were not available, give the numbers of studies and participants for which aggregate data were available. Report reasons for nonavailability of IPD. Include a flow diagram. | 8, 9, eFigure 1, and eAppendix 2 |
| Study characteristics | For each study, present information on key study and participant characteristics (such as description of interventions, numbers of participants, demographic data, unavailability of outcomes, funding source, and if applicable duration of follow-up). Provide (main) citations for each study. Where applicable, also report similar study characteristics for any studies not providing IPD. | 8, 9, eTable 1 and eTable 2 |
| IPD integrity | Report any important issues identified in checking IPD or state that there were none. | 8, 9, and eAppendix 2 |
| Risk of bias within studies | Present data on risk of bias assessments. If applicable, describe whether data checking led to the up-weighting or down-weighting of these assessments. Consider how any potential bias affects the robustness of meta-analysis conclusions. | 10, eTables 17-23 and eFigures 8-10 |
| Results of individual studies | For each comparison and for each main outcome (benefit or harm), for each individual study report the number of eligible participants for which data were obtained and show simple summary data for each intervention group (including, where applicable, the number of events), effect estimates, and confidence intervals. These may be tabulated or included on a forest plot. | eFigures 8-10 |
| Results of syntheses | Present summary effects for each meta-analysis undertaken, including confidence intervals and measures of statistical heterogeneity. State whether the analysis was prespecified, report the numbers of studies and participants and, where applicable, report the number of events on which it is based. | Table 1, Figures 1-4, and Supplementary material |
| | When exploring variation in effects due to patient or study characteristics, present summary interaction estimates for each characteristic examined, including confidence intervals and measures of statistical heterogeneity. State whether the analysis was prespecified. State whether any interaction is consistent across trials. | Supplementary material |
| | Provide a description of the direction and size of effect in terms meaningful to those who would put findings into practice. | Table 1, Figures 1-4, and Supplementary material |
| Risk of bias across studies | Present results of any assessment of risk of bias relating to the accumulated body of evidence, including any pertaining to the availability and representativeness of available studies, outcomes, or other variables. | Supplementary material |
| Additional analyses | Additional analyses | Supplementary material |
| **Discussion** | | |
| Summary of evidence | Summarize the main findings, including the strength of evidence for each main outcome. | 10 |
| Strengths and limitations | Discuss any important strengths and limitations of the evidence, including the benefits of access to IPD and any limitations arising from IPD that were not available. | 10-12 |
| Conclusions | Provide a general interpretation of the findings in the context of other evidence. | 10-12 |
| Implications | Consider relevance to key groups (such as policy makers, service providers, and service users). Consider implications for future research. | 10-12 |
| **Funding** | | |
| Funding | Describe sources of funding and other support (such as supply of IPD) and the role in the systematic review of those providing such support. | 13 |

## eAppendix 4: Systematic literature search procedures

**Data Sources and Searches**

Studies published between January 1970 and January 2015 were identified, without any language restriction, through electronic searches using MEDLINE, EMBASE and Scopus, and supplemented by scanning reference lists of articles identified and discussion with investigators. The computer-based search strategy is detailed below.

**Study Selection and Data Extraction**

Prospective observational studies were included if they had reported on the association of body mass index (BMI) and all-cause mortality. Prospective cohort studies or consortia thereof were eligible for inclusion if they satisfied all of the following criteria: 1) had information on weight, height, age and sex; 2) did not select participants on the basis of having previous chronic disease; 3) recorded overall or cause-specific deaths; and 4) had accrued more than 5 years of median follow-up (studies that had less than 5 years of follow-up did not contribute to the primary analysis in this report even if they had contributed to a participating consortium). A literature search flow chart is provided below.

Data on the following characteristics were extracted independently by five investigators (EDA, EP, ES, LOK and NB) according to a pre-specified protocol: full study name, study acronym, study location, and number of participants and number of deaths. Discrepancies were resolved by discussion and by adjudication of a sixth reviewer (PW). We used the most up-to-date or comprehensive information in cases of multiple publications.

**Search strategy**

| | |
|---|---|
| Publication database: | MEDLINE |
| #1 | "Body mass index” |
| #2 | Mortality OR death |
| #3 | "Cohort Studies"[Mesh] OR cohort OR prospective OR "risk ratio" OR "relative risk" OR "hazard ratio" OR "rate ratio" OR "risk ratios" OR "relative risks" OR "hazard ratios" OR "rate ratios" |
| #4 | "Animals"[Mesh] NOT "Humans"[Mesh] |
| Search: | (#1 AND #2 AND #3) NOT #4 |
| Restrictions: | None |
| Result: | 9,507 citations |

| | |
|---|---|
| Publication database: | EMBASE |
| #1 | (“body mass index”).af |
| #2 | (mortality OR deaths).af |
| #3 | (cohort OR prospective OR "risk ratio" OR "relative risk" OR "hazard ratio" OR "rate ratio" OR "risk ratios" OR "relative risks" OR "hazard ratios" OR "rate ratios").af |
| Search: | (#1 AND #2 AND #3) |
| Restrictions: | None |
| Result: | 7,230 citations |

| | |
|---|---|
| Publication database: | Scopus |
| #1 | TITLE-ABS-KEY (“body mass index”) |
| #2 | TITLE-ABS-KEY (mortality OR deaths) |
| #3 | TITLE-ABS-KEY(cohort OR prospective OR "risk ratio" OR "relative risk" OR "hazard ratio" OR "rate ratio" OR "risk ratios" OR "relative risks" OR "hazard ratios" OR "rate ratios") |
| Search: | (#1 AND #2 AND #3) |
| Restrictions: | None |
| Result: | 7,053 citations |

**Literature search flow chart**

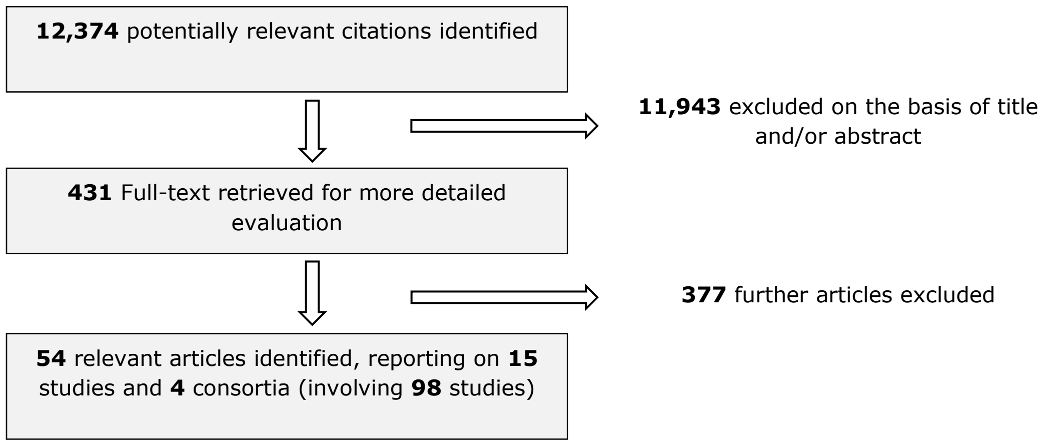

## eAppendix 5: References


1. Ulmer H, Kelleher C, Diem G, Concin H. Long-term tracking of cardiovascular risk factors among men and women in a large population-based health system. *European heart journal.* 2003;24(11):1004-1013.
2. De Henauw S, De Bacquer D, Fonteyne W, Stam M, Kornitzer M, De Backer G. Trends in the prevalence, detection, treatment and control of arterial hypertension in the Belgian adult population. *Journal of hypertension.* 1998;16(3):277-284.
3. Zhang H, Thijs L, Kuznetsova T, Fagard RH, Li X, Staessen JA. Progression to hypertension in the non-hypertensive participants in the Flemish Study on Environment, Genes and Health Outcomes. *Journal of hypertension.* 2006;24(9):1719-1727.
4. Keys A. Coronary heart disease in seven countries. 1970. *Nutrition (Burbank, Los Angeles County, Calif.).* 1997;13(3):250-252; discussion 249, 253.
5. Appleyard M. *The Copenhagen City Heart Study: Østerbroundersøgelsen: a book of tables with data from the first examination (1976-78) and a five year follow-up (1981-83).* Copenhagen City Heart Study Group. Medical Department B, Rigshospitalet, University Hospital of Copenhagen; 1988.
6. Schroll M, Jørgensen T, Ingerslev J. The Glostrup Population Studies, 1964-1992. *Danish medical bulletin.* 1992;39(3):204-207.
7. Reunanen A, Aromaa A, Pyorala K, Punsar S, Maatela J, Knekt P. The Social Insurance Institution's Coronary Heart Disease Study. *Acta Med Scand.* 1983;673(Suppl):1-120.
8. Pajunen P, Vartiainen E, Männistö S, Jousilahti P, Laatikainen T, Peltonen M. Intra-individual changes in body weight in population-based cohorts during four decades: the Finnish FINRISK study. *The European Journal of Public Health.* 2012;22(1):107-112.
9. Hakala S, Tilvis R, Strandberg T. Blood pressure and mortality in an older population. *Eur Heart J.* 1997;18:1019-1023.
10. Strandberg TE, Salomaa VV, Vanhanen HT, Naukkarinen VA, Sarna S, Miettinen TA. Mortality in participants and non-participants of a multifactorial prevention study of cardiovascular diseases: a 28 year follow up of the Helsinki Businessmen Study. *British heart journal.* 1995;74(4):449-454.
11. Myk L, Laakso M, Penttilä I, Pyörälä K. Asymptomatic hyperglycemia and cardiovascular risk factors in the elderly. *Atherosclerosis.* 1991;88(2):153-161.
12. Nissinen A, Tuomilehto J, Elo J, Salonen JT, Puska P. Implementation of a hypertension control program in the county of North Karelia, Finland. *Public Health Reports.* 1981;96(6):503.
13. Thomas F, Pannier B, Benetos A, Vischer UM. The impact of the metabolic syndrome–but not of hypertension–on all-cause mortality disappears in the elderly. *Journal of hypertension.* 2011;29(4):663-668.
14. Balkau B, Lange C, Fezeu L, et al. Predicting diabetes: clinical, biological, and genetic approaches data from the epidemiological study on the insulin resistance syndrome (DESIR). *Diabetes care.* 2008;31(10):2056-2061.
15. Fontbonne AM, Eschwège EM. Insulin and cardiovascular disease: Paris prospective study. *Diabetes care.* 1991;14(6):461-469.
16. Wagner A, Simon C, Evans A, et al. Physical Activity and Coronary Event Incidence in Northern Ireland and France The Prospective Epidemiological Study of Myocardial Infarction (PRIME). *Circulation.* 2002;105(19):2247-2252.
17. Löw M, Stegmaier C, Ziegler H, Rothenbacher D, Brenner H. Epidemiological investigations of the chances of preventing, recognizing early and optimally treating chronic diseases in an elderly population (ESTHER study). *Deutsche medizinische Wochenschrift (1946).* 2004;129(49):2643-2647.
18. Cremer P, Elster H, Labrot B, Kruse B, Muche R, Seidel D. Incidence rates of fatal and nonfatal myocardial infarction in relation to the lipoprotein profile: first prospective results from the Göttingen Risk, Incidence and Prevalence Study (GRIPS). *Klin Wochenschr.* 1988;66(Suppl XI):42-49.
19. Wilhelmsen L, Johansson S, Rosengren A, Wallin I, Dotevall A, Lappas G. Risk factors for cardiovascular disease during the period 1985–1995 in Göteborg, Sweden. The GOT-MONICA Project. *Journal of internal medicine.* 1997;242(3):199-211.
20. Assmann G, Cullen P, Heinrich J, Schulte H. Hemostatic variables in the prediction of coronary risk: results of the 8 year follow-up of healthy men in the Munster Heart Study (PROCAM). Prospective Cardiovascular Munster Study. *Israel journal of medical sciences.* 1996;32(6):364-370.
21. John U, Hensel E, Lüdemann J, et al. Study of Health In Pomerania (SHIP): a health examination survey in an east German region: objectives and design. *Sozial-und Präventivmedizin.* 2001;46(3):186-194.
22. Claessen H, Arndt V, Drath C, Brenner H. Overweight, obesity and risk of work disability: a cohort study of construction workers in Germany. *Occupational and environmental medicine.* 2009;66(6):402-409.
23. Panagiotakos DB, Pitsavos C, Chrysohoou C, et al. Epidemiology of overweight and obesity in a Greek adult population: the ATTICA Study. *Obesity Research.* 2004;12(12):1914-1920.
24. Sigurdsson E, Thorgeirsson G, Sigvaldason H, Sigfusson N. Prevalence of coronary heart disease in Icelandic men 1968–1986. *European heart journal.* 1993;14(5):584-591.
25. BIP Study Group. Secondary Prevention by Raising HDL Cholesterol and Reducing Triglycerides in Patients With Coronary Artery Disease The Bezafibrate Infarction Prevention (BIP) Study. *Circulation.* 2000;102(1):21-27.
26. Tanne D, Yaari S, Goldbourt U. High-Density Lipoprotein Cholesterol and Risk of Ischemic Stroke Mortality A 21-Year Follow-up of 8586 Men From the Israeli Ischemic Heart Disease Study. *Stroke.* 1997;28(1):83-87.
27. Modan M, Halkin H, Almog S, et al. Hyperinsulinemia. A link between hypertension obesity and glucose intolerance. *Journal of clinical investigation.* 1985;75(3):809.
28. Willeit J, Kiechl S. Prevalence and risk factors of asymptomatic extracranial carotid artery atherosclerosis. A population-based study. *Arteriosclerosis, Thrombosis, and Vascular Biology.* 1993;13(5):661-668.

29. Casiglia E, Spolaore P, Mormino P, et al. The CASTEL project (CArdiovascular STudy in the ELderly): protocol, study design, and preliminary results of the initial survey. *Cardiologia (Rome, Italy).* 1991;36(7):569-576.
30. Menotti A, Spagnolo A, Dima F, Meli P, Scanga M. The prediction of coronary heart disease in different population samples. *European journal of epidemiology.* 1992;8(4):521-526.
31. Fornari C, Donfrancesco C, Riva MA, et al. Social status and cardiovascular disease: a Mediterranean case. Results from the Italian Progetto CUORE cohort study. *BMC public health.* 2010;10(1):574.
32. Trevisan M, Schisterman E, Mennotti A, Farchi G, Conti S. Drinking Pattern and Mortality:: The Italian Risk Factor and Life Expectancy Pooling Project. *Annals of Epidemiology.* 2001;11(5):312-319.
33. Rodeghiero F, Tosetto A. The epidemiology of inherited thrombophilia: the VITA Project. Vicenza Thrombophilia and Atherosclerosis Project. *Thrombosis and haemostasis.* 1997;78(1):636-640.
34. Visscher T, Kromhout D, Seidell J. Long-term and recent time trends in the prevalence of obesity among Dutch men and women. *International journal of obesity and related metabolic disorders: journal of the International Association for the Study of Obesity.* 2002;26(9):1218-1224.
35. Mooy JM, Grootenhuis PA, de Vries H, et al. Prevalence and determinants of glucose intolerance in a Dutch Caucasian population: the Hoorn Study. *Diabetes care.* 1995;18(9):1270-1273.
36. Deeg D, Comijs H, Thomése G, Visser M. [The Longitudinal Ageing Study Amsterdam: a survey of 17 years of research into changes in daily functioning]. *Tijdschrift voor gerontologie en geriatrie.* 2009;40(6):217-227.
37. Klungel OH, de Boer A, Paes AH, Seidell JC, Bakker A. Cardiovascular diseases and risk factors in a population-based study in The Netherlands: agreement between questionnaire information and medical records. *The Netherlands journal of medicine.* 1999;55(4):177-183.
38. Hillege HL, Fidler V, Diercks GF, et al. Urinary albumin excretion predicts cardiovascular and noncardiovascular mortality in general population. *Circulation.* 2002;106(14):1777-1782.
39. Hofman A, Murad SD, van Duijn CM, et al. The Rotterdam Study: 2014 objectives and design update. *European journal of epidemiology.* 2013;28(11):889-926.
40. Rabanal KS, Lindman AS, Selmer RM, Aamodt G. Ethnic differences in risk factors and total risk of cardiovascular disease based on the Norwegian CONOR study. *European journal of preventive cardiology.* 2013;20(6):1013-1021.
41. Lindman AS, Veierød M, Tverdal A, Pedersen J, Selmer R. Nonfasting triglycerides and risk of cardiovascular death in men and women from the Norwegian Counties Study. *European journal of epidemiology.* 2010;25(11):789-798.
42. Holme I. Long-term survival in pre-specified groups at risk in the Oslo Study, 1972–1973. *Scandinavian journal of public health.* 2015:1403494814558157.
43. Jacobsen BK, Eggen AE, Mathiesen EB, Wilsgaard T, Njølstad I. Cohort profile: the Tromsø study. *International journal of epidemiology.* 2011:dyr049.
44. Fowkes F, Housley E, Cawood E, Macintyre C, Ruckley C, Prescott R. Edinburgh Artery Study: prevalence of asymptomatic and symptomatic peripheral arterial disease in the general population. *International journal of epidemiology.* 1991;20(2):384-392.
45. Shepherd J, Blauw GJ, Murphy MB, et al. The design of a prospective study of pravastatin in the elderly at risk (PROSPER). *The American journal of cardiology.* 1999;84(10):1192-1197.
46. Ballesteros-Pomar M, Rubio-Herrera M, Gutiérrez-Fuentes J, et al. Dietary habits and cardiovascular risk in the Spanish population: the DRECE study (I). *Annals of nutrition and metabolism.* 2000;44(3):108-114.
47. Lobo A, Saz P, Marcos G, Día J-L, De-la-Cámara C. The prevalence of dementia and depression in the elderly community in a southern european population: the Zaragoza study. *Archives of General Psychiatry.* 1995;52(6):497-506.
48. Walldius G, Jungner I, Kolar W, Holme I, Steiner E. High cholesterol and triglyceride values in Swedish males and females: increased risk of fatal myocardial infarction. First report from the AMORIS (Apolipoprotein related MOrtality RISk) study. *Blood pressure. Supplement.* 1991;4:35-42.
49. Larsson SC, Giovannucci E, Wolk A. Diabetes and colorectal cancer incidence in the cohort of Swedish men. *Diabetes Care.* 2005;28(7):1805-1807.
50. Rosengren A, Eriksson H, Larsson B, et al. Secular changes in cardiovascular risk factors over 30 years in Swedish men aged 50: the study of men born in 1913, 1923, 1933 and 1943. *Journal of internal medicine.* 2000;247(1):111-118.
51. Berglund G, Eriksson K, Israelsson B, et al. Cardiovascular risk groups and mortality in an urban Swedish male population: the Malmö Preventive Project. *Journal of internal medicine.* 1996;239(6):489-497.
52. Blomstrand A, Blomstrand C, Ariai N, Bengtsson C, Björkelund C. Stroke incidence and association with risk factors in women: a 32-year follow-up of the Prospective Population Study of Women in Gothenburg. *BMJ open.* 2014;4(10):e005173.
53. Wolk A, Larsson SC, Johansson J-E, Ekman P. Long-term fatty fish consumption and renal cell carcinoma incidence in women. *Jama.* 2006;296(11):1371-1376.
54. Lind L, Vessby B, Sundström J. The apolipoprotein B/AI ratio and the metabolic syndrome independently predict risk for myocardial infarction in middle-aged men. *Arteriosclerosis, thrombosis, and vascular biology.* 2006;26(2):406-410.
55. Kumle M, Weiderpass E, Braaten T, Persson I, Adami H-O, Lund E. Use of oral contraceptives and breast cancer risk the Norwegian-Swedish women's lifestyle and Health Cohort Study. *Cancer Epidemiology Biomarkers & Prevention.* 2002;11(11):1375-1381.
56. Onat A, Şenocak MŞ. Relative risk of factors for coronary heart disease in population with low cholesterol levels. *International journal of cardiology.* 1994;43(1):51-60.
57. Kapetanakis VV, Rudnicka AR, Wathern AK, et al. Adiposity in Early, Middle and Later Adult Life and Cardiometabolic Risk Markers in Later Life; Findings from the British Regional Heart Study. *PloS one.* 2014;9(12):e114289.
58. Kalina MA. B.U.P.A. (British United Provident Association). *Lancet (London, England).* 1972;1(7740):44-45.

59. Lawlor D, Bedford C, Taylor M, Ebrahim S. Geographical variation in cardiovascular disease, risk factors, and their control in older women: British Women's Heart and Health Study. *Journal of Epidemiology and Community Health.* 2003;57(2):134-140.
60. The Caerphilly and Speedwell Collaborative Group. Caerphilly and Speedwell collaborative heart disease studies. *Journal of epidemiology and community health.* 1984;38(3):259-262.
61. Syddall H, Sayer AA, Dennison E, Martin H, Barker D, Cooper C. Cohort profile: the Hertfordshire cohort study. *International journal of epidemiology.* 2005;34(6):1234-1242.
62. Meade TW. Design and intermediate results of the Lower Extremity Arterial Disease Event Reduction (LEADER)* trial of bezafibrate in men with lower extremity arterial disease [ISRCTN41194621]. *Trials.* 2001;2(4):195.
63. Upton MN, McConnachie A, McSharry C, et al. Intergenerational 20 year trends in the prevalence of asthma and hay fever in adults: the Midspan family study surveys of parents and offspring. *Bmj.* 2000;321(7253):88-92.
64. Million Women Study Collaborative Group. The Million Women Study: design and characteristics of the study population. *Breast Cancer Research.* 1999;1(1):73.
65. Fletcher A, Jones D, Bulpitt C, Tulloch A. The MRC trial of assessment and management of older people in the community: objectives, design and interventions [ISRCTN23494848]. *BMC health services research.* 2002;2(1):21.
66. Hart CL, Smith GD. Alcohol consumption and mortality and hospital admissions in men from the Midspan Collaborative cohort study. *Addiction.* 2008;103(12):1979-1986.
67. Meade TW, Stirling Y. The Northwick Park Heart Studies: contrasts between the two factor VII assays used. J Thromb Haemost. 2012;10(3):482-4.
68. Hawthorne V, Watt G, Hart C, Hole D, Smith GD, Gillis C. Cardiorespiratory disease in men and women in urban Scotland: baseline characteristics of the Renfrew/Paisley (Midspan) Study population. *Scottish Medical Journal.* 1995;40(4):102-107.
69. Tunstall-Pedoe H, Woodward M, Tavendale R, A'Brook R, McCluskey MK. Comparison of the prediction by 27 different factors of coronary heart disease and death in men and women of the Scottish Heart Health Study: cohort study. *Bmj.* 1997;315(7110):722-729.
70. Rudnicka A, MT-ISA S, Meade T. Associations of plasma fibrinogen and factor VII clotting activity with coronary heart disease and stroke: prospective cohort study from the screening phase of the Thrombosis Prevention Trial. *Journal of Thrombosis and Haemostasis.* 2006;4(11):2405-2410.
71. Sudlow C, Gallacher J, Allen N, et al. UK biobank: an open access resource for identifying the causes of a wide range of complex diseases of middle and old age. *PLoS medicine.* 2015;12(3):e1001779.
72. Rose G, Tunstall-Pedoe H, Heller R. UK heart disease prevention project: incidence and mortality results. *The Lancet.* 1983;321(8333):1062-1066.
73. Liao J. Long-term follow-up of the West of Scotland Coronary Prevention Study. *Current atherosclerosis reports.* 2008;10(4):283.
74. Marmot MG, Shipley MJ, Rose G. Inequalities in death—specific explanations of a general pattern? *The Lancet.* 1984;323(8384):1003-1006.
75. Hinnouho G-M, Czernichow S, Dugravot A, et al. Metabolically healthy obesity and the risk of cardiovascular disease and type 2 diabetes: the Whitehall II cohort study. *European heart journal.* 2015;36(9):551-559.
76. Riboli E, Hunt K, Slimani N, et al. European Prospective Investigation into Cancer and Nutrition (EPIC): study populations and data collection. *Public health nutrition.* 2002;5(6b):1113-1124.
77. Davidson KW, Schwartz JE, Kirkland SA, et al. Relation of inflammation to depression and incident coronary heart disease (from the Canadian Nova Scotia Health Survey [NSHS95] Prospective Population Study). *The American journal of cardiology.* 2009;103(6):755-761.
78. DaGenais GR, Robitaille NM, Lupien PJ, et al. First coronary heart disease event rates in relation to major risk factors: Québec Cardiovascular Study. *The Canadian journal of cardiology.* 1990;6(7):274-280.
79. Alavanja MC, Sandler DP, McMaster SB, et al. The Agricultural Health Study. *Environmental health perspectives.* 1996;104(4):362.
80. Downs JR, Clearfield M, Tyroler HA, et al. Air Force/Texas Coronary Atherosclerosis Prevention Study (AFCAPS/TEXCAPS): additional perspectives on tolerability of long-term treatment with lovastatin. *The American journal of cardiology.* 2001;87(9):1074-1079.
81. Schatzkin A, Subar AF, Thompson FE, et al. Design and Serendipity in Establishing a Large Cohort with Wide Dietary Intake Distributions The National Institutes of Health–American Association of Retired Persons Diet and Health Study. *American journal of epidemiology.* 2001;154(12):1119-1125.
82. Szklo M, Barnes R, Folsom A, et al. The Atherosclerosis Risk in Communities (ARIC) Study: design and objectives. The ARIC investigators. *American Journal of Epidemiology.* 1989;129(4):687-702.
83. Mai V, Flood A, Peters U, Lacey JV, Schairer C, Schatzkin A. Dietary fibre and risk of colorectal cancer in the Breast Cancer Detection Demonstration Project (BCDDP) follow-up cohort. *International journal of epidemiology.* 2003;32(2):234-239.
84. Torio CM, Klassen AC, Curriero FC, Caballero B, Helzlsouer K. The modifying effect of social class on the relationship between body mass index and breast cancer incidence. *American journal of public health.* 2010;100(1):146.
85. Link LB, Canchola AJ, Bernstein L, et al. Dietary patterns and breast cancer risk in the California Teachers Study cohort. *The American journal of clinical nutrition.* 2013;98(6):1524-1532.
86. Garfinkel L, Heath C. Cancer Prevention Study II: the American Cancer Society prospective study. *Stat Bull.* 1992;73:21-29.
87. Suchy-Dicey AM, Wallace ER, Elkind MS, et al. Blood pressure variability and the risk of all-cause mortality, incident myocardial infarction, and incident stroke in the cardiovascular health study. *American journal of hypertension.* 2013:hpt092.

88. Boyle E. Biological patterns in hypertension by race, sex, body weight, and skin color. *Jama.* 1970;213(10):1637-1643.
89. Miura K, Daviglus ML, Dyer AR, et al. Relationship of blood pressure to 25-year mortality due to coronary heart disease, cardiovascular diseases, and all causes in young adult men: the Chicago Heart Association Detection Project in Industry. *Archives of Internal Medicine.* 2001;161(12):1501-1508.
90. Cornoni-Huntley J, Ostfeld AM, Taylor JO, et al. Established populations for epidemiologic studies of the elderly: study design and methodology. *Aging (Milan, Italy).* 1993;5(1):27-37.
91. Rutter MK, Meigs JB, Sullivan LM, D'Agostino RB, Wilson PW. C-reactive protein, the metabolic syndrome, and prediction of cardiovascular events in the Framingham Offspring Study. *Circulation.* 2004;110(4):380-385.
92. Pai JK, Mukamal KJ, Rimm EB. Long-term alcohol consumption in relation to all-cause and cardiovascular mortality among survivors of myocardial infarction: the Health Professionals Follow-up Study. *European heart journal.* 2012;33(13):1598-1605.
93. Kagan A, Harris BR, Winkelstein W, et al. Epidemiologic studies of coronary heart disease and stroke in Japanese men living in Japan, Hawaii and California: demographic, physical, dietary and biochemical characteristics. *Journal of chronic diseases.* 1974;27(7):345-364.
94. Folsom AR, Kushi LH, Anderson KE, et al. Associations of general and abdominal obesity with multiple health outcomes in older women: the Iowa Women's Health Study. *Arch Intern Med.* 2000;160(14):2117-2128.
95. Friedman LA, Morrison JA, Daniels SR, McCarthy WF, Sprecher DL. Sensitivity and specificity of pediatric lipid determinations for adult lipid status: findings from the Princeton Lipid Research Clinics Prevalence Program Follow-up Study. *Pediatrics.* 2006;118(1):165-172.
96. Luepker RV, Murray DM, Jacobs Jr DR, et al. Community education for cardiovascular disease prevention: risk factor changes in the Minnesota Heart Health Program. *American journal of public health.* 1994;84(9):1383-1393.
97. Luepker RV, Jacobs DR, Gillum RF, Folsom AR, Prineas RJ, Blackburn H. Population risk of cardiovascular disease: the Minnesota Heart Survey. *Journal of chronic diseases.* 1985;38(8):671-682.
98. Bild DE, Bluemke DA, Burke GL, et al. Multi-ethnic study of atherosclerosis: objectives and design. *American journal of epidemiology.* 2002;156(9):871-881.
99. Stamler J, Neaton JD, Cohen JD, et al. Multiple risk factor intervention trial revisited: a new perspective based on nonfatal and fatal composite endpoints, coronary and cardiovascular, during the trial. *Journal of the American Heart Association.* 2012;1(5):e003640.
100. Sacco R, Wolf PA, Kannel W, McNamara P. Survival and recurrence following stroke. The Framingham study. *Stroke.* 1982;13(3):290-295.
101. Kato I, Akhmedkhanov A, Koenig K, Toniolo PG, Shore RE, Riboli E. Prospective study of diet and female colorectal cancer: the New York University Women's Health Study. 1997.
102. Idler EL, Russell LB, Davis D. Survival, functional limitations, and self-rated health in the NHANES I epidemiologic follow-up study, 1992. *American journal of epidemiology.* 2000;152(9):874-883.
103. Gillum R, Obisesan TO. Physical activity, cognitive function, and mortality in a US national cohort. *Annals of epidemiology.* 2010;20(4):251-257.
104. Pleis JR, Lucas JW, Ward BW. Summary health statistics for U.S. adults: National Health Interview Survey, 2008. *Vital and health statistics. Series 10, Data from the National Health Survey.* 2009(242):1-157.
105. Zhang C, Rexrode KM, van Dam RM, Li TY, Hu FB. Abdominal obesity and the risk of all-cause, cardiovascular, and cancer mortality sixteen years of follow-up in US women. *Circulation.* 2008;117(13):1658-1667.
106. Belanger CF, Hennekens CH, Rosner B, Speizer FE. The nurses' health study. *AJN The American Journal of Nursing.* 1978;78(6):1039-1040.
107. Andriole GL, Crawford ED, Grubb RL, et al. Prostate cancer screening in the randomized Prostate, Lung, Colorectal, and Ovarian Cancer Screening Trial: mortality results after 13 years of follow-up. *Journal of the National Cancer Institute.* 2012.
108. Hennekens C. Final report on the aspirin component of the ongoing Physicians Health Study. *New England Journal of Medicine.* 1989;321(3):129-135.
109. Garcia-Palmieri M, Feliberti M, Costas Jr R, et al. An epidemiological study on coronary heart disease in Puerto Rico: The Puerto Rico Heart Health Program. *Boletin de la Asociacion Medica de Puerto Rico.* 1969;61(6):174-179.
110. Barrett-Connor E. Why Women Have Less Heart Disease Than Men and How Diabetes Modifies Women's Usual Cardiac Protection: A 40-Year Rancho Bernardo Cohort Study. *Global heart.* 2013;8(2):95-104.
111. Wynder EL, Lemon FR, Bross IJ. Cancer and coronary artery disease among seventh-day adventists. *Cancer.* 1959;12(5):1016-1028.
112. Lee ET, Welty TK, Fabsitz R, et al. The Strong Heart Study A study of cardiovascular disease in American Indians: design and methods. *American journal of epidemiology.* 1990;132(6):1141-1155.
113. Napier JA, Metzner H, Johnson BC. Limitations of morbidity and mortality data obtained from family histories--a report from the Tecumseh community health study. *American journal of public health.* 1972;62(1):30-35.
114. Boice Jr JD, Mandel JS, Doody MM, Yoder RC, McGowan R. A health survey of radiologic technologists. *Cancer.* 1992;69(2):586-598.
115. Greenlee H, White E, Patterson RE, Kristal AR. Supplement use among cancer survivors in the Vitamins and Lifestyle (VITAL) study cohort. *Journal of Alternative & Complementary Medicine.* 2004;10(4):660-666.
116. Hsia J, Aragaki A, Bloch M, LaCroix AZ, Wallace R, Investigators W. Predictors of angina pectoris versus myocardial infarction from the Women's Health Initiative Observational Study. *The American journal of cardiology.* 2004;93(6):673-678.
117. Rexrode KM, Lee I-M, Cook NR, Hennekens CH, Buring JE. Baseline characteristics of participants in the Women's Health Study. *Journal of women's health & gender-based medicine.* 2000;9(1):19-27.

118. Einhorn PT, Davis BR, Massie BM, et al. The Antihypertensive and Lipid Lowering Treatment to Prevent Heart Attack Trial (ALLHAT) heart failure validation study: diagnosis and prognosis. *American heart journal.* 2007;153(1):42-53.
119. Banks E, Redman S, Jorm L, et al. Cohort profile: the 45 and up study. *International journal of epidemiology.* 2008;37(5):941-947.
120. Collaborative Aneurysm Screening Study Group. A comparative study of the prevalence of abdominal aortic aneurysms in the United Kingdom, Denmark, and Australia. *Journal of medical screening.* 2001;8(1):46-50.
121. Dunstan DW, Zimmet PZ, Welborn TA, et al. The Australian diabetes, obesity and lifestyle study (AusDiab)—methods and response rates. *Diabetes research and clinical practice.* 2002;57(2):119-129.
122. Andrews G, Cheok F, Carr S. The Australian longitudinal study of ageing. *Australian Journal on Ageing.* 1989;8(2):31-35.
123. Zhu K, Hunter M, James A, Lim EM, Walsh JP. Associations between body mass index, lean and fat body mass and bone mineral density in middle-aged Australians: The Busselton Healthy Ageing Study. *Bone.* 2015;74:146-152.
124. Simons LA, Simons J, Friedlander Y, McCallum J. Usefulness of fasting plasma glucose to predict mortality or coronary heart disease in persons≥ 60 years of age without diabetes mellitus or in those with undiagnosed diabetes mellitus (from The Dubbo Study). *The American journal of cardiology.* 2008;102(7):831-834.
125. Norman PE, Flicker L, Almeida OP, Hankey GJ, Hyde Z, Jamrozik K. Cohort profile: the health in men study (HIMS). *International Journal of Epidemiology.* 2009;38(1):48-52.
126. Bennett SA, Magnus P. Trends in cardiovascular risk factors in Australia. Results from the National Heart Foundation's Risk Factor Prevalence Study, 1980-1989. *The Medical Journal of Australia.* 1994;161(9):519-527.
127. Huxley R, Barzi F, Stolk R, et al. Ethnic comparisons of obesity in the Asia-Pacific region: protocol for a collaborative overview of cross-sectional studies. *Obesity reviews: an official journal of the International Association for the Study of Obesity.* 2005;6(3):193-198.
128. Norman PE, Jamrozik K, Lawrence-Brown MM, et al. Population based randomised controlled trial on impact of screening on mortality from abdominal aortic aneurysm. *Bmj.* 2004;329(7477):1259.
129. Giles G, English D, Riboli E, Lambert R. The Melbourne Collaborative Cohort Study. Paper presented at: Nutrition and lifestyle: opportunities for cancer prevention. European Conference on Nutrition and Cancer held in Lyon, France on 21-24 June, 2003.2002.
130. Jiang J, Tang Z, Meng XJ, Futatsuka M. Demographic determinants for change in activities of daily living: a cohort study of the elderly people in Beijing. *Journal of Epidemiology.* 2002;12(3):280-286.
131. Wu X, Gu D, Wu Y, et al. [An evaluation on effectiveness of worksite-based intervention for cardiovascular disease during 1974-1998 in capital iron and steel company of Beijing]. *Zhonghua yu fang yi xue za zhi [Chinese journal of preventive medicine].* 2003;37(2):93-97.
132. Wu X, Duan X, Gu D, Hao J, Tao S, Fan D. Prevalence of hypertension and its trends in Chinese populations. *International journal of cardiology.* 1995;52(1):39-44.
133. Yang L, Yang G, Zhou M, et al. Body mass index and mortality from lung cancer in smokers and nonsmokers: a nationally representative prospective study of 220,000 men in China. *International journal of cancer.* 2009;125(9):2136-2143.
134. Wu N, Tang X, Wu Y, et al. Cohort profile: the Fangshan Cohort Study of cardiovascularepidemiology in Beijing, China. *Journal of epidemiology.* 2014;24(1):84.
135. Blot WJ, Li J-Y, Taylor PR, et al. Nutrition intervention trials in Linxian, China: supplementation with specific vitamin/mineral combinations, cancer incidence, and disease-specific mortality in the general population. *Journal of the national cancer institute.* 1993;85(18):1483-1491.
136. Tao L, Wang R, Gao Y-T, Yuan J-M. Impact of postdiagnosis smoking on long-term survival of cancer patients: the Shanghai Cohort Study. *Cancer epidemiology biomarkers & prevention.* 2013;22(12):2404-2411.
137. Shu X-O, Li H, Yang G, et al. Cohort Profile: The Shanghai Men's Health Study. *International journal of epidemiology.* 2015:dyv013.
138. Zheng W, Chow W-H, Yang G, et al. The Shanghai Women's Health Study: rationale, study design, and baseline characteristics. *American journal of epidemiology.* 2005;162(11):1123-1131.
139. Geng G. Serial epidemiological study of hypertension in Tianjin. *Zhonghua liu xing bing xue za zhi= Zhonghua liuxingbingxue zazhi.* 1989;10(2):70-73.
140. Xiang-Zhen X, Lubin JH, Jun-Yao L, et al. A cohort study in southern China of tin miners exposed to radon and radon decay products. *Health physics.* 1993;64(2):120-131.
141. Zhang W, Jiang C, Lam T, et al. A prospective cohort study on the effect of occupational dust exposure and smoking on mortality. *Chinese journal of industrial hygiene and occupational diseases.* 2002;20(5):365-368.
142. Ho SC, Woo J, Sham A, Chan SG, Ashley L. A 3-year follow-up study of social, lifestyle and health predictors of cognitive impairment in a Chinese older cohort. *International Journal of Epidemiology.* 2001;30(6):1389-1396.
143. Tamakoshi A, Ohno Y, Yamada T, et al. Depressive mood and suicide among middle-aged workers: findings from a prospective cohort study in Nagoya, Japan. *Journal of epidemiology.* 2000;10(3):173-178.
144. Tominaga M, Eguchi H, Manaka H, Igarashi K, Kato T, Sekikawa A. Impaired glucose tolerance is a risk factor for cardiovascular disease, but not impaired fasting glucose. The Funagata Diabetes Study. *Diabetes care.* 1999;22(6):920-924.
145. Hata J, Ninomiya T, Hirakawa Y, et al. Secular trends in cardiovascular disease and its risk factors in Japanese: half century data from the Hisayama Study (1961-2009). *Circulation.* 2013:CIRCULATIONAHA. 113.002424.
146. Matsuo T, Sairenchi T, Iso H, et al. Age-and gender-specific BMI in terms of the lowest mortality in Japanese general population. *Obesity.* 2008;16(10):2348-2355.
147. Imano H, Iso H, Sato S, et al. Determinants of platelet aggregation in 50–70-year-old men from three Japanese communities. *Atherosclerosis.* 2002;165(2):327-334.

148. Tamakoshi A, Yoshimura T, Inaba Y, et al. Profile of the JACC Study. *Journal of epidemiology.* 2005;15 (Supplement_I):S4-S8.
149. Tsugane S, Sobue T. Baseline survey of JPHC study design and participation rate. *Journal of epidemiology.* 2001;11(6sup):24-29.
150. Tomita M, Mizuno S, Yamanaka H, et al. Does hyperuricemia aeffect mortality? A prospective cohort study of Japanese male workers. *Journal of epidemiology.* 2000;10(6):403-409.
151. Kodama K, Ozasa K, Katayama H, Shore RE, Okubo T. Radiation effects on cancer risks in the life span study cohort. *Radiation protection dosimetry.* 2012:ncs180.
152. Tsuji I, Nishino Y, Tsubono Y, et al. Follow-up and mortality profiles in the Miyagi Cohort Study. *Journal of Epidemiology.* 2004;14(Supplement_I):S2-S6.
153. Kasamatsu T, Morioka S, Hashimoto T, Kinoshita H, Yamada H, Tamaki T. Epidemiological study on the bone mineral density of inhabitants in Miyama Village, Wakayama Prefecture (Part I) Background of study population and sampling method. *Journal of Bone and Mineral Metabolism.* 1991;9(1):50-55.
154. Ohkubo T, Imai Y, Tsuji I, et al. Home blood pressure measurement has a stronger predictive power for mortality than does screening blood pressure measurement: a population-based observation in Ohasama, Japan. *Journal of hypertension.* 1998;16(7):971-975.
155. Tsuji I, Nishino Y, Ohkubo T, et al. A prospective cohort study on national health insurance beneficiaries in Ohsaki, Miyagi Prefecture, Japan: study design, profiles of the subjects and medical cost during the first year. *Journal of epidemiology.* 1998;8(5):258-263.
156. Sato S, Nakamura M, Iida M, et al. Plasma fibrinogen and coronary heart disease in urban Japanese. *American journal of epidemiology.* 2000;152(5):420-423.
157. Soyama Y, Miura K, Morikawa Y, et al. High-Density Lipoprotein Cholesterol and Risk of Stroke in Japanese Men and Women The Oyabe Study. *Stroke.* 2003;34(4):863-868.
158. Imai K, Nakachi K. Cross sectional study of effects of drinking green tea on cardiovascular and liver diseases. *Bmj.* 1995;310(6981):693-696.
159. Nakayama T, Date C, Yokoyama T, Yoshiike N, Yamaguchi M, Tanaka H. A 15.5-year follow-up study of stroke in a Japanese provincial city The Shibata Study. *Stroke.* 1997;28(1):45-52.
160. Nozaki A, Hirao K, Sugimoto C, et al. A comparative study of the health conditions of elderly people living alone, elderly couples and the bedridden elderly at home in a rural area of Shiga Prefecture: special reference to morbidity rate and blood pressure, electrocardiograph and blood examination data. *[Nihon koshu eisei zasshi] Japanese journal of public health.* 1993;40(9):850-858.
161. Ohnishi H, Saitoh S, Shimamoto K. The Tanno and Sobetsu Study. *Nihon rinsho. Japanese journal of clinical medicine.* 2010;68(5):839-842.
162. Marugame T, Sobue T, Satoh H, et al. Lung cancer death rates by smoking status: Comparison of the Three-Prefecture Cohort study in Japan to the Cancer Prevention Study II in the USA. *Cancer science.* 2005;96(2):120-126.
163. Nakamura K, Sakurai M, Miura K, et al. Homeostasis model assessment of insulin resistance and the risk of cardiovascular events in middle-aged non-diabetic Japanese men. *Diabetologia.* 2010;53(9):1894-1902.
164. Hankin JH, Stram DO, Arakawa K, et al. Singapore Chinese Health Study: development, validation, and calibration of the quantitative food frequency questionnaire. *Nutrition and cancer.* 2001;39(2):187-195.
165. Khoo CM, Sairazi S, Taslim S, et al. Ethnicity modifies the relationships of insulin resistance, inflammation, and adiponectin with obesity in a multiethnic Asian population. *Diabetes care.* 2011;34(5):1120-1126.
166. Tan CE, Emmanuel SC, Tan B-Y, Jacob E. Prevalence of diabetes and ethnic differences in cardiovascular risk factors. The 1992 Singapore National Health Survey. *Diabetes care.* 1999;22(2):241-247.
167. Jee SH, Ohrr H, Sull JW, Yun JE, Ji M, Samet JM. Fasting serum glucose level and cancer risk in Korean men and women. *Jama.* 2005;293(2):194-202.
168. Jee SH, O'Donnell MP, Suh I, Kim IS. The relationship between modifiable health risks and future medical care expenditures: the Korea Medical Insurance Corporation (KMIC) Study. *American Journal of Health Promotion.* 2001;15(4):244-255.
169. Lee E-H, Park SK, Ko K-P, et al. Cigarette smoking and mortality in the Korean Multi-center Cancer Cohort (KMCC) Study. *Journal of Preventive Medicine and Public Health.* 2010;43(2):151-158.
170. Bae J-M, Li Z-M, Shin M-H, Kim D-H, Lee M-S, Ahn Y-O. Pulmonary tuberculosis and lung cancer risk in current smokers: The Seoul Male Cancer Cohort Study. *Journal of Korean medical science.* 2013;28(6):896-900.
171. Chuang S-Y, Bai C-H, Chen W-H, Lien L-M, Pan W-H. Fibrinogen independently predicts the development of ischemic stroke in a Taiwanese population CVDFACTS Study. *Stroke.* 2009;40(5):1578-1584.
172. Wu M-M, Chen H-C, Chen C-L, et al. A prospective study of gynecological cancer risk in relation to adiposity factors: cumulative incidence and association with plasma adipokine levels. *PloS one.* 2014;12(9).
173. Liu H-C, Wang S-J, Fuh J-L, et al. The Kinmen Neurological Disorders Survey (KINDS): A study of a chinese population. *Neuroepidemiology.* 1997;16(2):60-68.
174. Vathesatogkit P, Woodward M, Tanomsup S, et al. Cohort Profile: The electricity generating authority of Thailand study. *International journal of epidemiology.* 2012;41(2):359-365.
175. Argos M, Kalra T, Rathouz PJ, et al. Arsenic exposure from drinking water, and all-cause and chronic-disease mortalities in Bangladesh (HEALS): a prospective cohort study. *The Lancet.* 2010;376(9737):252-258.
176. Pednekar MS, Hakama M, Hebert JR, Gupta PC. Association of body mass index with all-cause and cause-specific mortality: findings from a prospective cohort study in Mumbai (Bombay), India. *International journal of epidemiology.* 2008;37(3):524-535.
177. Sankaranarayanan R, Ramadas K, Thomas G, et al. Effect of screening on oral cancer mortality in Kerala, India: a cluster-randomised controlled trial. *The Lancet.* 2005;365(9475):1927-1933.

# Figure 1: Association of BMI with all-cause mortality, by geographical region

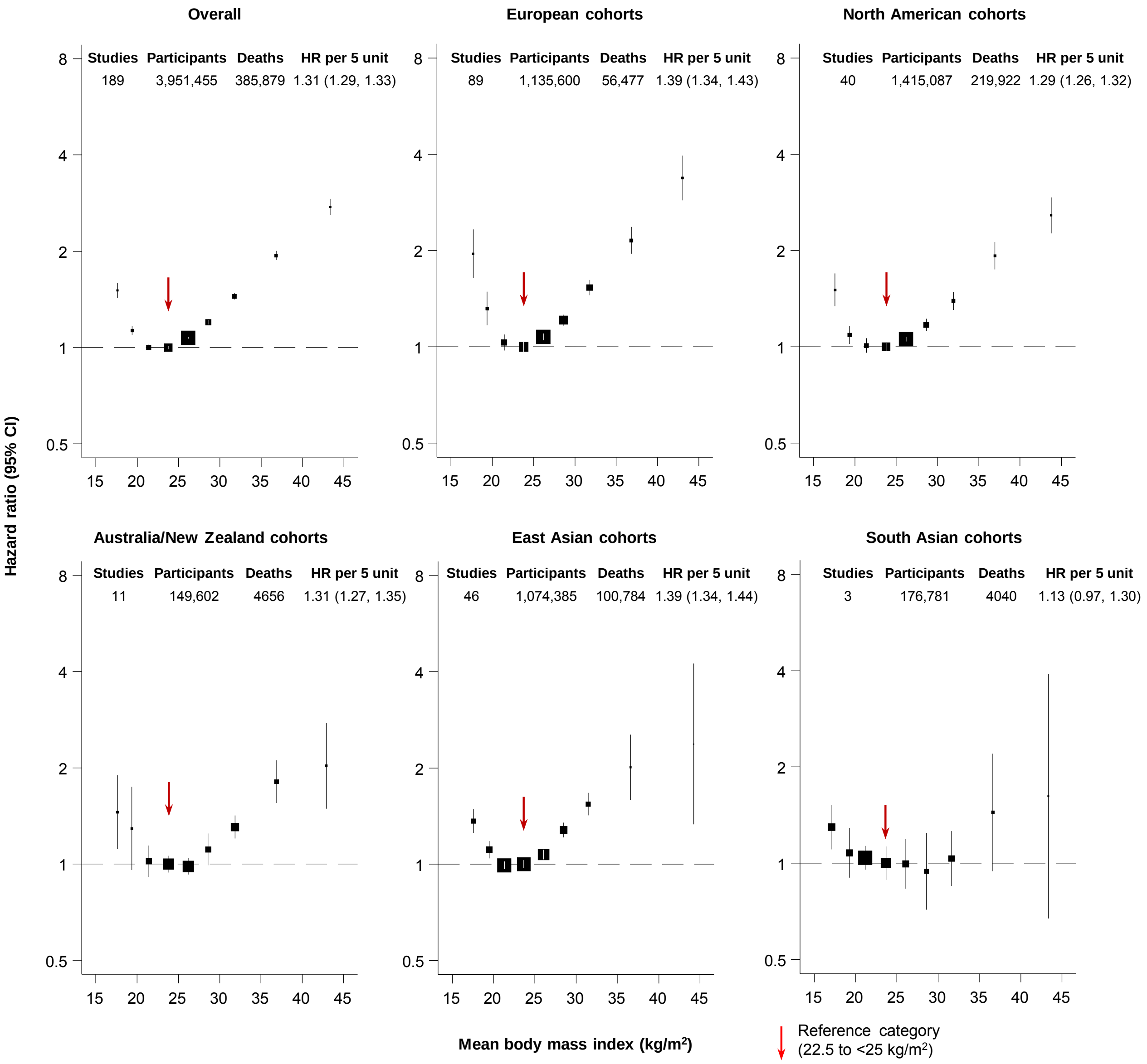


HR, hazard ratio per 5 kg/m$^2$ of BMI in the range BMI >25 kg/m$^2$ (and its 95% CI).

Analyses are restricted to never-smokers without pre-existing chronic disease, exclude first 5 years of follow-up, and include data from all geographical regions.

BMI groups: 15 to <18.5, 18.5 to <20, 20 to <22.5, 22.5 to <25, 25 to <27.5, 27.5 to <30, 30 to <35, 35 to <40, and 40 to <60 kg/m$^2$. Reference category (arrow) is 22.5 to <25 kg/m$^2$.

CIs are from floating variance estimates (reflecting independent variability within each category, including the reference category). Areas of squares are proportional to the information content.

# Figure 2: Association of BMI with all-cause mortality, by baseline age group

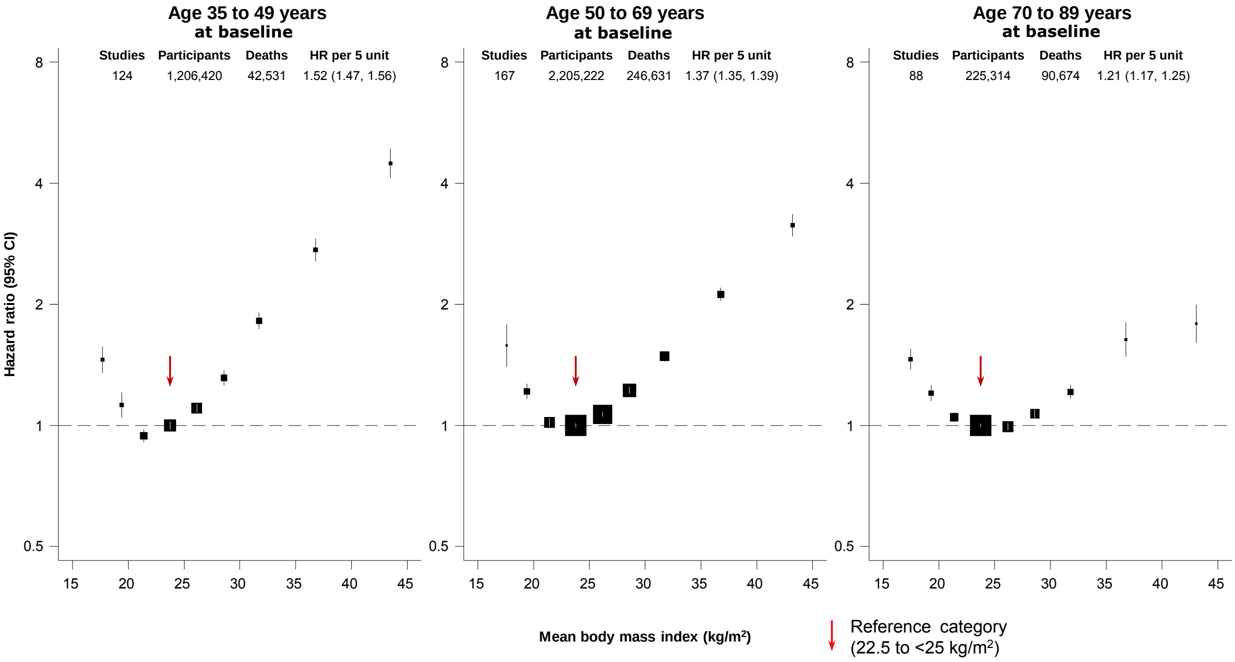


HR, hazard ratio per 5 kg/m$^2$ of BMI in the range BMI >25 kg/m$^2$ (and its 95% CI).

Analyses are restricted to never-smokers without pre-existing chronic disease, exclude first 5 years of follow-up, and include data from all geographical regions.

BMI groups: 15 to <18.5, 18.5 to <20, 20 to <22.5, 22.5 to <25, 25 to <27.5, 27.5 to <30, 30 to <35, 35 to <40, and 40 to <60 kg/m$^2$. Reference category (arrow) is 22.5 to <25 kg/m$^2$.

CIs are from floating variance estimates (reflecting independent variability within each category, including the reference category). Areas of squares are proportional to the information content.

## Figure 3: Association of BMI with all-cause mortality, by sex

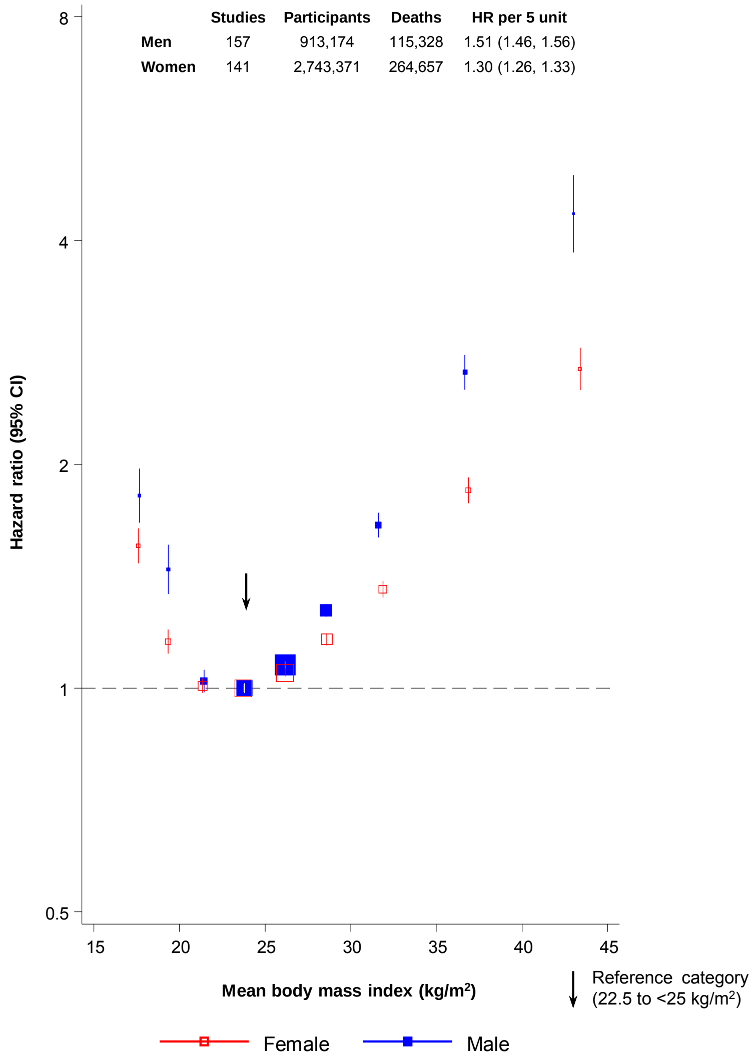


HR, hazard ratio per 5 $kg/m^2$ of BMI in the range BMI >25 $kg/m^2$ (and its 95% CI).

Analyses are restricted to never-smokers without pre-existing chronic disease, exclude first 5 years of follow-up, and include data from all geographical regions.

BMI groups: 15 to <18.5, 18.5 to <20, 20 to <22.5, 22.5 to <25, 25 to <27.5, 27.5 to <30, 30 to <35, 35 to <40, and 40 to <60 $kg/m^2$. Reference category (arrow) is 22.5 to <25 $kg/m^2$.

CIs are from floating variance estimates (reflecting independent variability within each category, including the reference category). Areas of squares are proportional to the information content.

## Figure 4: Association of BMI with mortality, by major underlying cause

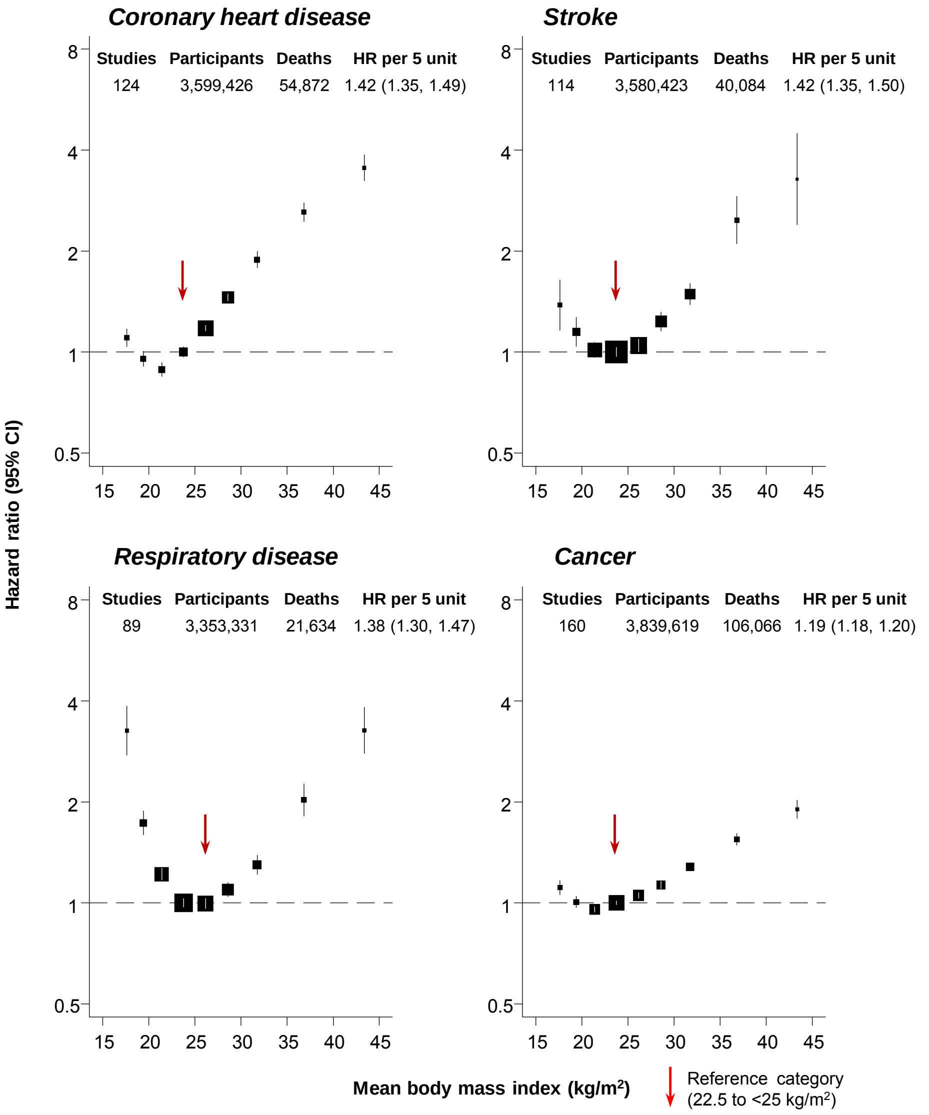


HR, hazard ratio per 5 kg/m$^2$ of BMI in the range BMI >25 kg/m$^2$ (and its 95% CI).

Analyses are restricted to never-smokers without pre-existing chronic disease, exclude first 5 years of follow-up, and include data from all geographical regions.

BMI groups: 15 to <18.5, 18.5 to <20, 20 to <22.5, 22.5 to <25, 25 to <27.5, 27.5 to <30, 30 to <35, 35 to <40, and 40 to <60 kg/m$^2$. Reference category (arrow) is 22.5 to <25 kg/m$^2$.

CIs are from floating variance estimates (reflecting independent variability within each category, including the reference category). Areas of squares are proportional to the information content.
